# The genome of the medicinal plant *Uncaria rhynchophylla* provides new insights into monoterpenoid indole alkaloid metabolism and its molecular regulatory mechanism

**DOI:** 10.1186/s43897-025-00187-7

**Published:** 2026-02-03

**Authors:** Detian Mu, Lingyun Wan, Yingying Shao, Limei Pan, Xinghui Liu, Iain W. Wilson, Zhixing Qing, Yu Zhou, Ying Lu, Yingjie He, Lina Zhu, Jine Fu, Shugen Wei, Xiaojun Ma, Chi Song, Deyou Qiu, Qi Tang

**Affiliations:** 1 College of Horticulture, National Research Center of Engineering Technology for Utilization of Botanical Functional Ingredients, Yuelushan Lab, Hunan Agricultural University, Changsha, 410128 China; 2 Guangxi Key Laboratory of High-Quality Formation and Utilization of Dao-di Herbs, National Center for TCM Inheritance and Innovation, Guangxi Botanical Garden of Medicinal Plants, Nanning, 530023 China; 3 CSIRO Agriculture and Food, Canberra, ACT 2601 Australia; 4 College of Veterinary Medicine, Hunan Agricultural University, Changsha, 410128 China; 5 Southern University of Science and Technology, Shenzhen, 518055 China; 6 Institute of Medicinal Plant Development, Chinese Academy of Medical Sciences, Peking Union Medical College, Beijing, 100193 China; 7 Wuhan Benagen Technology Company Ltd., Wuhan, 430075 China; 8 State Key Laboratory of Tree Genetics and Breeding, Research Institute of Forestry, Chinese Academy of Forestry, Beijing, 100091 China; 9 State Key Laboratory for Quality Ensurance and Sustainable Use of Dao-di Herbs, Beijing, 100700 China

**Keywords:** MIA biosynthesis, *Uncaria rhynchophylla*, Genome, Transcription factor, Secondary metabolism

## Abstract

The high-quality chromosomal-level genome assembly (627.72 Mb, contig N50 1.80 Mb) of *Uncaria rhynchophylla*, a Chinese herb used for hypertension and Alzheimer's disease. Integrated genomic and transcriptomic analyses identified 46,909 genes, including 72 *UrWRKY* transcription factors. Sixty-four candidate genes for monoterpenoid indole alkaloid (MIA) biosynthesis were screened. Functional validation confirmed UrTDC6 catalyzes tryptamine formation and UrLAMT1/UrLAMT2 catalyze loganin production. Correlation analysis linked MIA content with *UrWRKY* expression, identifying *UrWRKY37* as a key regulator. UrWRKY37 directly activates *UrTDC* and *UrSGD* transcription. Overexpression in hairy roots upregulated MIA pathway genes and significantly increased accumulation of isocorynoxeine, corynoxeine, and isorhynchophylline. This work elucidates MIA biosynthesis and its transcriptional regulation in *U. rhynchophylla*.

**Supplementary Information:**

The online version contains supplementary material available at 10.1186/s43897-025-00187-7.

## Core

The chromosomal-level genome of the medicinal plant *Uncaria rhynchophylla* was assembled, resolving critical gaps for elucidating monoterpenoid indole alkaloid (MIA) biosynthesis. Functional validation confirmed that UrTDC6 catalyzes L-tryptophan to tryptamine, while UrLAMT1 and UrLAMT2 convert loganate to loganin. Among 72 identified UrWRKY transcription factors, UrWRKY37 emerged as a pivotal regulator. Dual-luciferase and yeast one-hybrid assays demonstrated UrWRKY37 directly binds and activates promoters of *UrTDC* and *UrSGD*. UrWRKY37 boosted production of bioactive alkaloids in hairy roots by upregulating MIA pathway genes.

## Gene and accession number

The accession numbers of the genes used in this study are as follows: *UrTDC6* (OP669359), *UrLAMT1* (PV781159), *UrLAMT2* (PV81160), *UrSGD* (OP669353), *UrWRKY37* (OP669360).

## Introduction

Hypertension, a leading risk factor for cardiovascular diseases, affects over 1 billion individuals globally, with nearly 500 million cases reported in China alone (Bromfield & Muntner 2013; Yin et al. 2022). Concurrently, Alzheimer’s disease (AD), impacting 40 million people worldwide and projected to triple in prevalence by 2050, poses a growing threat to aging populations (Scheltens et al. 2021). The urgent need for therapies targeting these conditions has driven interest in *Uncaria rhynchophylla*, a medicinal herb endemic to tropical China, which produces unique monoterpenoid indole alkaloids (MIAs) such as rhynchophylline (RIN) and isorhynchophylline (IRN) (Gattuso et al. 2004; Yang et al. 2022). These compounds exhibit dual therapeutic potential for hypertension and AD (Zhou & Zhou 2010; Fu et al. 2021). However, reliance on traditional plant extraction methods and low production yields hinder their commercial viability. Therefore, methods to significantly increase the production of RIN and IRN through sustainable and environmentally friendly methods are crucial.

**Fig. 1:**
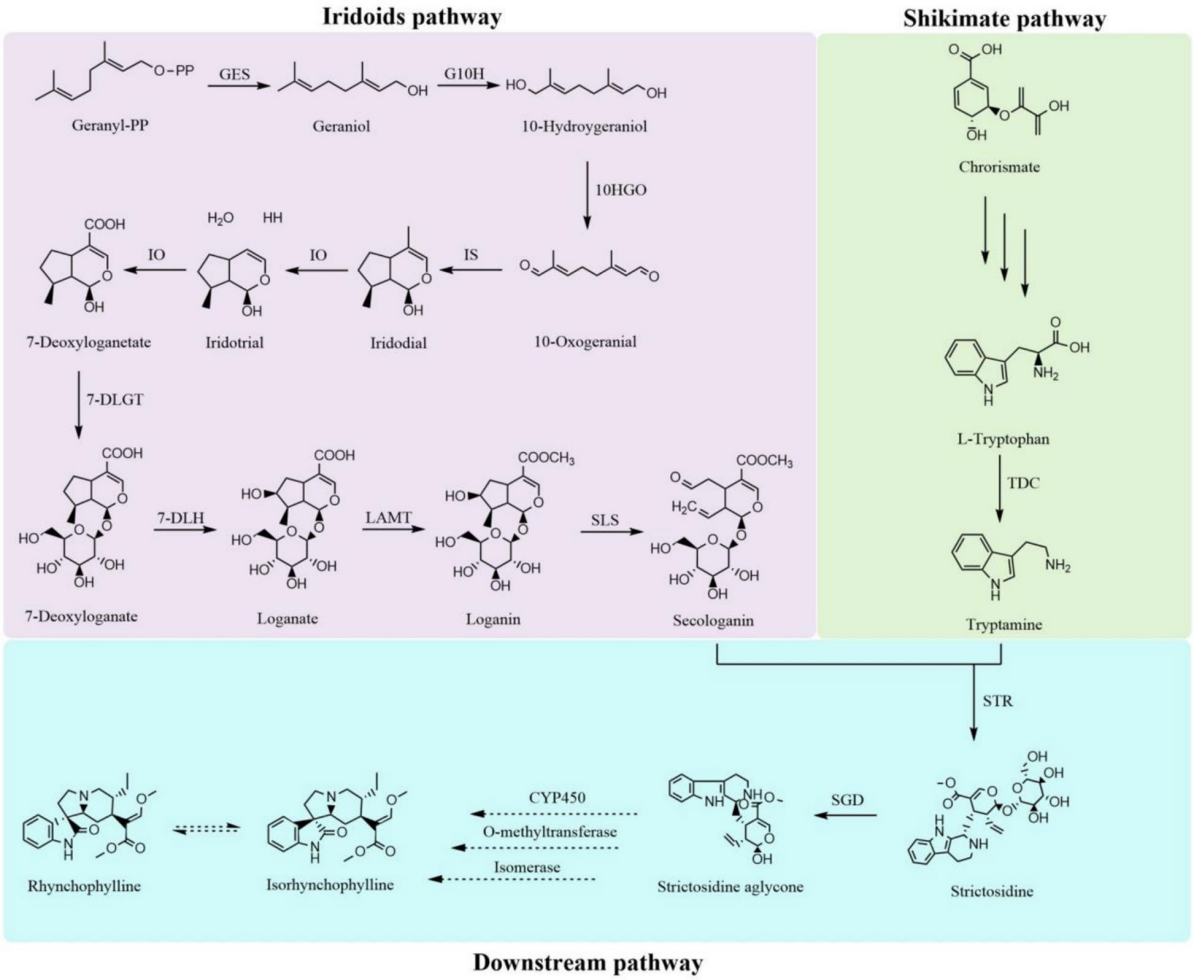
The putative biosynthesis pathway of IRN and RIN in *U. rhynchophylla*. The dashed arrows represent the speculated biosynthesis pathway

The biosynthesis of RIN and IRN in *U. rhynchophylla* initiates with strictosidine, formed via condensation of secologanin and tryptamine catalyzed by strictosidine synthase (STR) (Mu et al. 2023; Guo et al. 2014). The former provides a monoterpenoid moiety in the iridoid pathway, which involves a nine-step enzymatic reaction from geranyl diphosphate to the final product, whereas the latter provides an indole moiety in the shikimate pathway (Hallard et al. 1997; Stöckigt et al. 2008). To date, two *UrSTR* genes, UrSTR1/5, have been identified, both of which can catalyse the formation of strictosidine with tryptamine and secologanin (Kulhar and Rajakumara 2023). However, other key enzymes have not been identified in *U. rhynchophylla*. The loganate O-methyltransferase (LAMT) is a key enzyme in the iridoid pathway that catalyses the conversion of loganate into loganin (Jiang et al. 2023). Furthermore, tryptophan is converted into tryptamine, the final product of the shikimate pathway, by tryptophan decarboxylase (TDC) (Kang et al. 2021). Hence, LAMT and TDC are recognized as crucial rate-limiting enzymes in the MIA pathway. However, the regulatory, functional, and active sites of UrLAMT and UrTDC in the biosynthesis of RIN and IRN are still largely unknown in *U. rhynchophylla* (Fig. 1).

The complete genome sequence offers valuable genetic resources for investigating key enzyme-encoding genes involved in the biosynthesis of active medicinal ingredients, as well as for identifying gene families (Liu et al. 2020; Zeng et al. 2024). Transcription factors (TFs), significant gene families within plants, regulate plant growth, development, hormonal responsiveness, and most notably, the pathways of secondary metabolites (Duan et al. 2022; Wu et al., 2023; Zhao et al. 2024). Some TFs, such as those in the *bHLH* and *GATA* gene families, have been identified and shown to regulate RIN and IRN production in *U. rhynchophylla* (Shao et al. 2024a; Wang et al., 2022). UrGATA7 and UrGATA8 are implicated in modulating the expression of six essential enzyme genes, leading to the contents of IRN, RIN, COX, and ICOX under diverse light conditions. Recent studies have highlighted the critical role of WRKY TFs in the regulation of secondary metabolic pathways. For instance, in *Ophiorrhiza pumila*, OpWRKY2 functions as a positive regulator, directly binding to and activating *OpTDC*, thereby increasing camptothecin production (Hao et al. 2021); Shao et al. 2024b). Conversely, OpWRKY6 negatively regulates camptothecin synthesis by downregulating key enzyme genes from the iridoid and shikimate pathways (Wu et al., 2023). Additionally, the overexpression of *SmWRKY1* in *Salvia miltiorrhiza* has been shown to enhance tanshinone biosynthesis (Chen et al. 2024). However, the involvement of WRKY TFs in the regulation of IRN and RIN biosynthesis in *U. rhynchophylla* remains to be elucidated.

To address these knowledge gaps, we present a chromosomal-level genome assembly of *U. rhynchophylla* and systematically characterize *UrWRKY* gene family. By integrating metabolomic and transcriptomic analyses, we identified two *UrLAMT* genes and one *UrTDC* gene. Both UrLAMT proteins exhibited catalytic activity in the conversion of loganate to loganin, and tryptophan was converted to tryptamine under the enzymatic action of UrTDC. Furthermore, we demonstrate that UrWRKY37 can directly activate *UrTDC* and *UrSGD* expression, significantly boosting RIN and IRN accumulation in transgenic hairy roots. Our study presents two distinct approaches to metabolic engineering for enhancing IRN and RIN production, with a focus on biosynthetic genes and transcription factors.

## Results

### Identification of tissue-specific accumulation of MIAs in *U. rhynchophylla* via target metabolome analysis

**Fig. 2:**
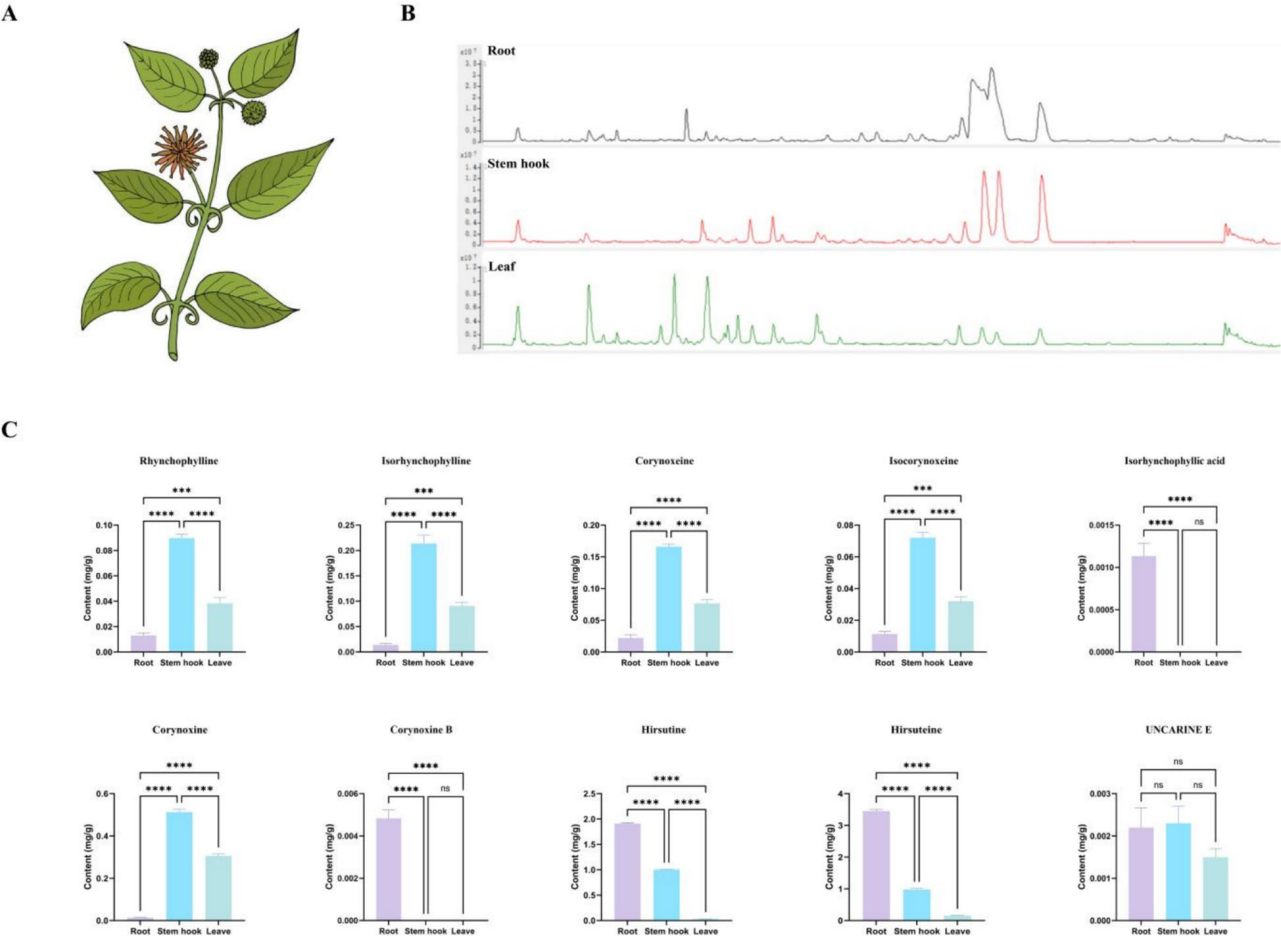
The accumulation patterns of MIAs in three different tissues of *U. rhynchophylla*. **A** Different tissues of *U. rhynchophylla* plants. **B** Total ion chromatogram of different tissues in *U. rhynchophylla*. **C** Tissue-specific accumulation of ten target MIAs. *** represents *p* < 0.001, **** represents *p* < 0.0001, ns represent no difference

To elucidate the MIA accumulation pattern in *U. rhynchophylla*, leaf, root, and stem hook tissues were collected separately (Fig. 2A). Ten typical MIAs were quantified by matching the mass spectra of standard substances. The results revealed significant variation in the accumulation of MIAs in different *U. rhynchophylla* tissues. Most bioactive ingredients, such as RIN, IRN, COX, ICOX, corynoxine, and uncarine E, accumulated at higher levels in the stem hooks than in the roots and leaves (Fig. 2C). Only a few MIAs, such as hirsuteine and hirsutine, showed greater accumulation in roots than in other tissues. Interestingly, isorhynchophyllic acid and corynoxine B were detected only in the roots.

### Genome sequencing and assembly

**Fig. 3:**
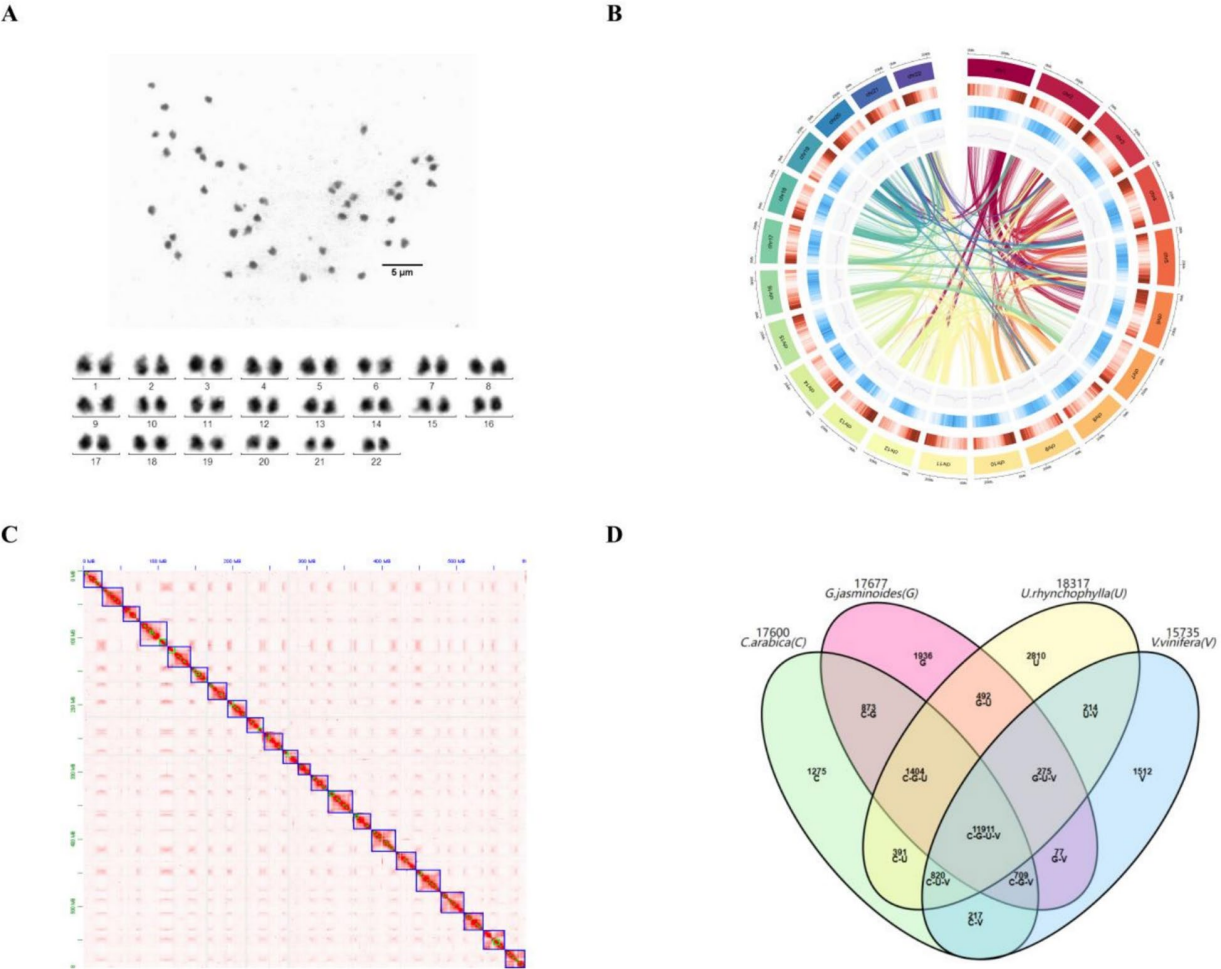
Chromosomes, Hi-C map, and genome circle plot of assembly for *U. rhynchophylla*. **A** The karyotype displays 22 chromosomes in *U. rhynchophylla*. **B** Graphic of the genome assembly and sequencing analysis of *U. rhynchophylla* (from outside to inside: 1. pseudochromosome number; 2. gene density; 3. repeat density; 4. miRNA density; 5. rRNA density; 6. snRNA density; 7. tRNA density). **C** A Hi-C contact map illustrating the genomic interactions. Anchoring contigs on 22 chromosomes of *U. rhynchophylla*

Before genome sequencing was initiated, a genome survey of *U. rhynchophylla* was performed to estimate its genome size and heterozygosity using Illumina NovaSeq. The genome size and heterozygosity of *U. rhynchophylla* were estimated to be 621.62 Mb, with high heterozygosity (1.07%) and repetition (55.11%) based on 19-mer analysis (Fig. S1, Table S1, S2). Three different sequencing technologies, Oxford Nanopore Technologies (ONT), Illumina NovaSeq, and Hi-C sequencing, were used to obtain whole-genome information. The assembled genome size of *U. rhynchophylla* was found to be 627.70 Mb, with a contig N50 of 1.83 Mb, an average length of 0.97 Mb, and a maximum length of 10.17 Mb (Table S3). Hi-C sequencing technology was used to aid in assembling contigs at the chromosome level (Doncheva et al. 2019). According to karyotype analysis (Fig. 3A), *U. rhynchophylla* is a diploid plant with 22 chromosomes (2n = 2x = 44 = 18 m + 26Sm (2SAT)). Hi-C facilitated the anchoring of a total of 43.68% of the *U. rhynchophylla* genome on the 22 chromosomes, and the short reads were aligned to the genome, with a mapping rate of 94.46% (Fig. 3B, C; Table S4). BUSCO (Benchmarking Universal Single‐Copy Orthologues) was used to evaluate the integrity of the genome assembly. The results revealed that 96.1% of the BUSCOs were complete BUSCOs (Table S5). Therefore, this high-quality *U. rhynchophylla* genome provides genetic resources for the exploration of MIA biosynthetic pathways and TFs involved in transcription regulation.

A total of 46,909 coding genes were predicted, with an average gene size of 3,396 bp, an average coding sequence (CDS) length of 1,080 bp, and an average of 5.08 exons per gene (Table S6). According to seven public databases, 39,447 genes (87.04%) could be annotated with biological functions, with the InterProScan website providing annotations for the largest number of genes (81.05%) (Table S7). Approximately 39.10% of the *U. rhynchophylla* genome consisted of transposable elements (TEs) that were identified by RepeatMasker. Among the TEs, the two most common types of repetitive elements in the *U. rhynchophylla* genome were long terminal repeat (LTR) retrotransposons and long interspersed nuclear elements (LINEs), accounting for 34.35% and 2.53%, respectively (Table S8).

Gene family clustering was conducted among *U. rhynchophylla* and 17 other species. A total of 45,320 genes (96.57%) were grouped into 18,317 gene families. Among these genes, 2,020 (11.02%) were specific to *U. rhynchophylla,* with an average number of genes per family of 1.81 (Table S9, Fig. S2). The specific genes found in the *U. rhynchophylla* genome that were enriched in various gene ontology (GO) functional categories were analysed, with the most enriched terms being the general GO term GO:0016705 (oxidoreductase activity with incorporation or reduction of molecular oxygen) and the specific GO term GO:0009820 (indole alkaloid metabolic process), which may influence the levels of the primary bioactive metabolites present in this medicinal plant (Fig. S3). A phylogenetic tree was constructed based on the 142 gene families that are shared by all species as single-copy orthologous gene families. The findings revealed that *U. rhynchophylla*, *C. arabica*, and *G. jasminoides* formed a cluster, which is consistent with the fact that they both belong to the Rubiaceae family and were positioned on a branch, having diverged approximately 44.7 million years ago (Mya) (23.2–70.6 Mya) (Fig. S4). There were 11,911 common protein-encoding gene families that were identified among *G. jasminoides*, *U. rhynchophylla*, *V. vinifera*, and *C. arabica*. Compared with these other species, *U. rhynchophylla* had the greatest number of unique genes (Fig. 3D). The results of the gene family clustering analysis revealed that 2044 gene families in *U. rhynchophylla* were expanded, and that 937 gene families were contracted, representing greater numbers than those in the majority of the species we compared it to (Fig. S5). The Ks values, which represent the rates of synonymous substitution per gene, are distributed among collinear paralogous genes and were used to identify whole-genome duplication (WGD) events in *U. rhynchophylla*, *G. jasminoides*, *C. arabica*, and *V. vinifera*. The results revealed an ancient WGD event in *U. rhynchophylla* (Fig. S6).

**Fig. 4:**
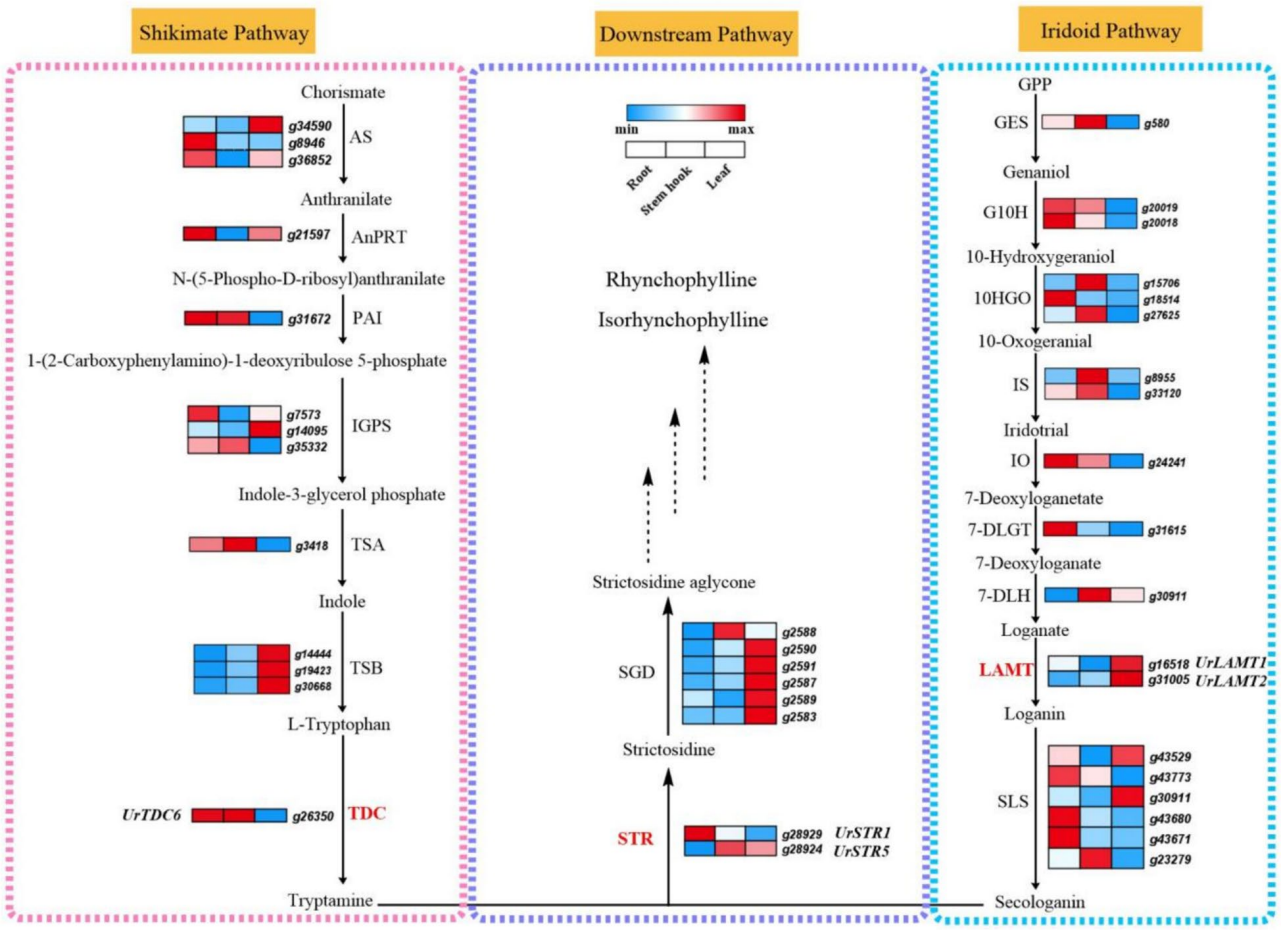
The expression patterns of candidate genes in RIN and IRN biosynthesis pathway. Enzymes with catalytic functions reported in this study or previously are marked in red

The high-quality chromosome-level *U. rhynchophylla* genome provides a good platform for screening candidate key enzyme-encoding genes in the MIA biosynthetic pathways and related transcription factors. A total of 64 candidate genes encoding 32 enzymes in the RIN and IRN upstream biosynthetic pathways were identified from the genome databases (Table S10) (Kadota et al., 2020; Mu et al. 2023). To further investigate the expression patterns of these candidate genes, three different tissues (roots, stem hooks and leaves) of *U. rhynchophylla* were selected for transcriptome sequencing. The gene expression data for *U. rhynchophylla* were subsequently annotated and analysed using the *U. rhynchophylla* genome assembly database from this study. The correlations between key enzyme-encoding genes involved in the RIN upstream biosynthetic pathway and the accumulation patterns of RIN and IRN in the three tissues were analysed (Fig. 4). In addition, 219 *cytochrome P450* (CYP450) genes, which might encode enzymes that participate in RIN and IRN biosynthesis, were identified in the *U. rhynchophylla* genome (Table S11).

### UrTDC6 catalyses the conversion of L-tryptophan to tryptamine in *U. rhynchophylla*

Tryptophan decarboxylase (TDC) is a key rate-limiting enzyme in the biosynthesis of MIAs. It can catalyse the conversion of L-tryptophan to tryptamine by the cofactor pyridoxal-5′-phosphate (PLP) (Qu et al. 2019). Therefore, it is crucial to verify whether it has a catalytic function in *U. rhynchophylla*. Six *UrTDC* genes are present in the *U. rhynchophylla* genome. Among these genes, two *UrTDC* genes were not full-length, and among the remaining four genes, only *UrTDC6* had detectable expression levels based on transcriptome database analysis. *UrTDC6* expression was highest in the stem hooks, followed by the roots (Fig. 4), which was consistent with the qRT‒PCR results (Fig. S7).

**Fig. 5:**
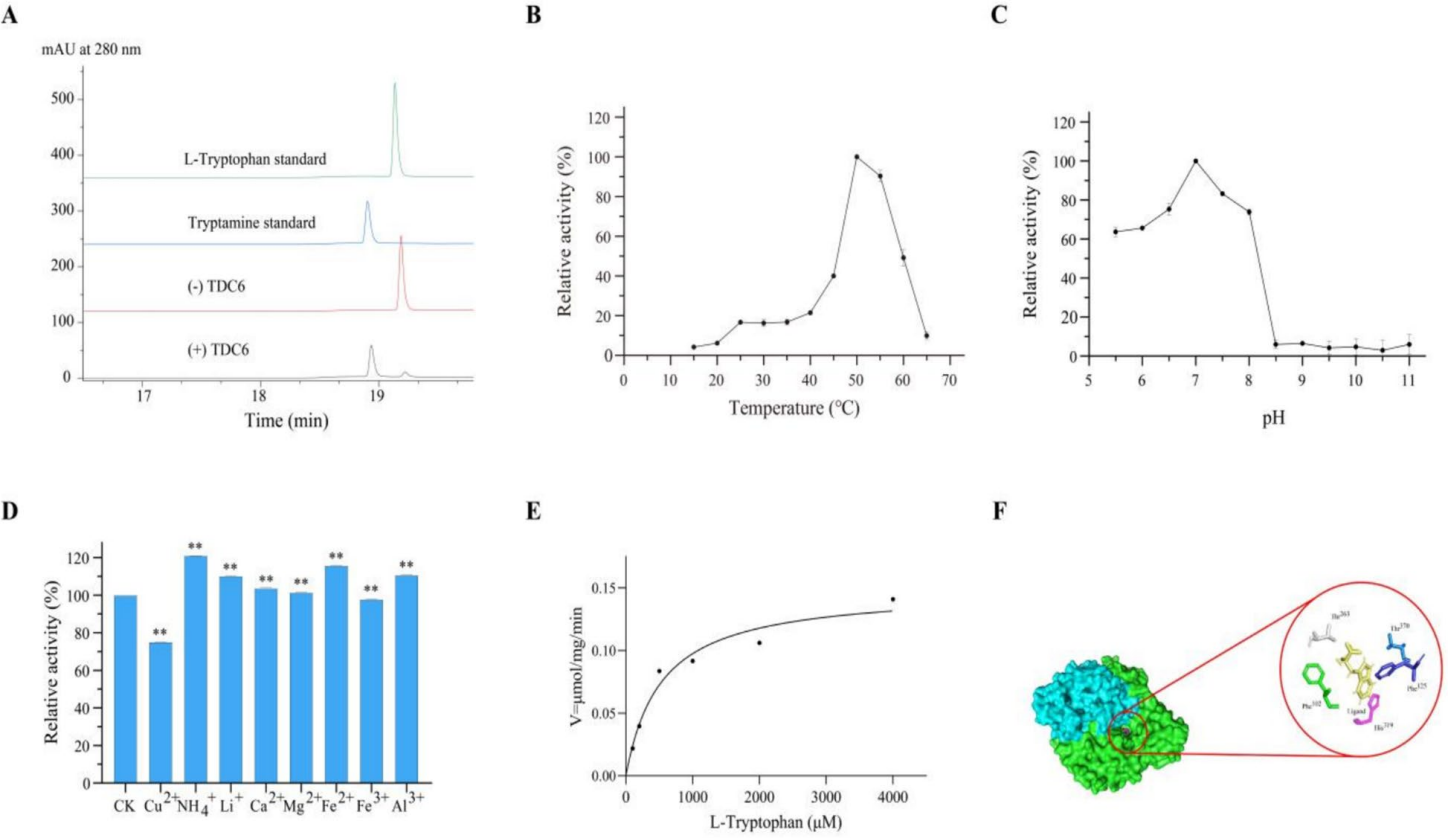
The enzyme activity assay of UrTDC6. **A** HPLC detection showed that the enzymatic reaction yielded the presence of tryptamine in the reaction mixture catalyzed by UrTDC6. **B** The impact of temperature (15–65 °C) on the catalytic reaction was assessed, revealing that the activity towards tryptamine was maximized at 50 °C. **C** A range of buffers spanning pH from 5.5 to 11.0 was employed to investigate the impact of pH on the enzymatic activity of the purified recombinant UrTDC6. The enzymatic activity towards tryptamine reached its maximum at pH 7.0. **D** The impact of metal ions on the activity of the UrTDC6 enzyme. ** represents *p* < 0.01. **E** Enzyme kinetics of UrTDC6 were determined using the Michaelis–Menten equation. **F** Docking results for L-tryptophan of UrTDC6

The results of the bioinformatics analysis revealed that the *UrTDC6* sequence was 1530 bp in length and encoded 509 amino acids, with a predicted molecular weight of 57.02 kDa. The theoretical pI, instability index and grand average of hydropathicity (GRAVY) were computed to be 5.68, 49.41 and 0.041, respectively. Multiple sequence alignment revealed that UrTDC6 belongs to the *TDC* gene family, which shares a highly conserved DDC/GAD/HDC/TyrDC pyridoxal 5’-phosphate attachment site with other TDCs (Fig. S8). Phylogenetic tree analysis revealed that TDCs can be classified into two groups (Fig. S9). UrTDC6 had high similarity (more than 75%) with TDCs from other species, and *Millettia speciosa* MsTDC had the highest level of homology with UrTDC6 (88.45%). With a sequence identity of 72.15%, *C. roseus* CsTDC was used as a template to model the three-dimensional structure of UrTDC6. The protein structure of UrTDC6 consists of two homologous subunits assembled into a homodimer, each containing 509 amino acid residues. The predicted 3-D structure of UrTDC6 was used for molecular docking with L-tryptophan as a substrate. The results showed that the catalytic activity pocket of UrTDC6 consists of two subunits, denoted by green and blue. The binding energy was calculated to be −7.696 kcal/mol. In addition, the substrate L-tryptophan is surrounded by five residues (Thr-263, Thr-370, Phe-125, His-319, and Phe-102) that are within its hydrogen bonding distance (Fig. 5F). Therefore, it was hypothesized that UrTDC6 may have catalytic activity to decarboxylate L-tryptophan to form tryptamine.

To characterize the catalytic function of the UrTDC6 protein, it was overexpressed in *E. coli*, followed by separation and purification using Ni–NTA affinity chromatography. Sodium dodecyl sulfate‒polyacrylamide gel electrophoresis (SDS‒PAGE) was conducted to determine the expression and purification of UrTDC6 (Fig. S10). According to the HPLC results, the enzymatic reaction yielded the presence of tryptamine in the reaction mixture, which was catalysed by UrTDC6. In contrast, tryptamine was not detected in the boiled control group (Fig. 5A). The highest relative activity of the protein was observed at 50 °C, whereas at lower temperatures, the protein activity was very weak. When the temperature was 15 °C, only 4.2% of the purified recombinant protein remained active (Fig. 5B). The optimal pH for UrTDC6 was pH 7.0 in phosphate-buffered saline (PBS). Recombinant protein activity decreased rapidly with increasing pH and was almost 0 at pH 8.5 (Fig. 5C). In addition, the recombinant protein was strongly inhibited by the metal ion Cu^2+^, whereas NH_4_^+^, Li^+^, Fe^2+^, and Al^3+^ promoted this process (Fig. 5D). These findings revealed that the activity of the UrTDC6 protein is affected by the presence of metal ions. Finally, to obtain the kinetic constants of the UrTDC6 protein, a series of L-tryptophan substrate concentration gradients of were established for the enzymatic reaction under the optimal reaction conditions described above. Kinetic analysis of UrTDC6 showed that the values for the *K*_m_, *k*_cat_, and catalytic efficiency were 0.53 mM, 0.14 s^−1^, and 271.96 s^−1.^M^−1^, respectively (Fig. 5E).

### Both UrLAMT1 and UrLAMT2 catalyse the conversion of loganate to loganin in *U. rhynchophylla*

Recent reports have indicated that loganate can be converted into loganin under LAMT catalysis (Liu et al. 2012). Two *UrLAMT* genes were identified from the *U. rhynchophylla* genome, and *UrLAMT1* and *UrLAMT2* were expressed in three different tissues of *U. rhynchophylla*. The expression levels of both *UrLAMT1* and *UrLAMT2* were greater in leaves than in the other tissues, which aligns with the qRT‒PCR results (Fig. S11).

**Fig. 6:**
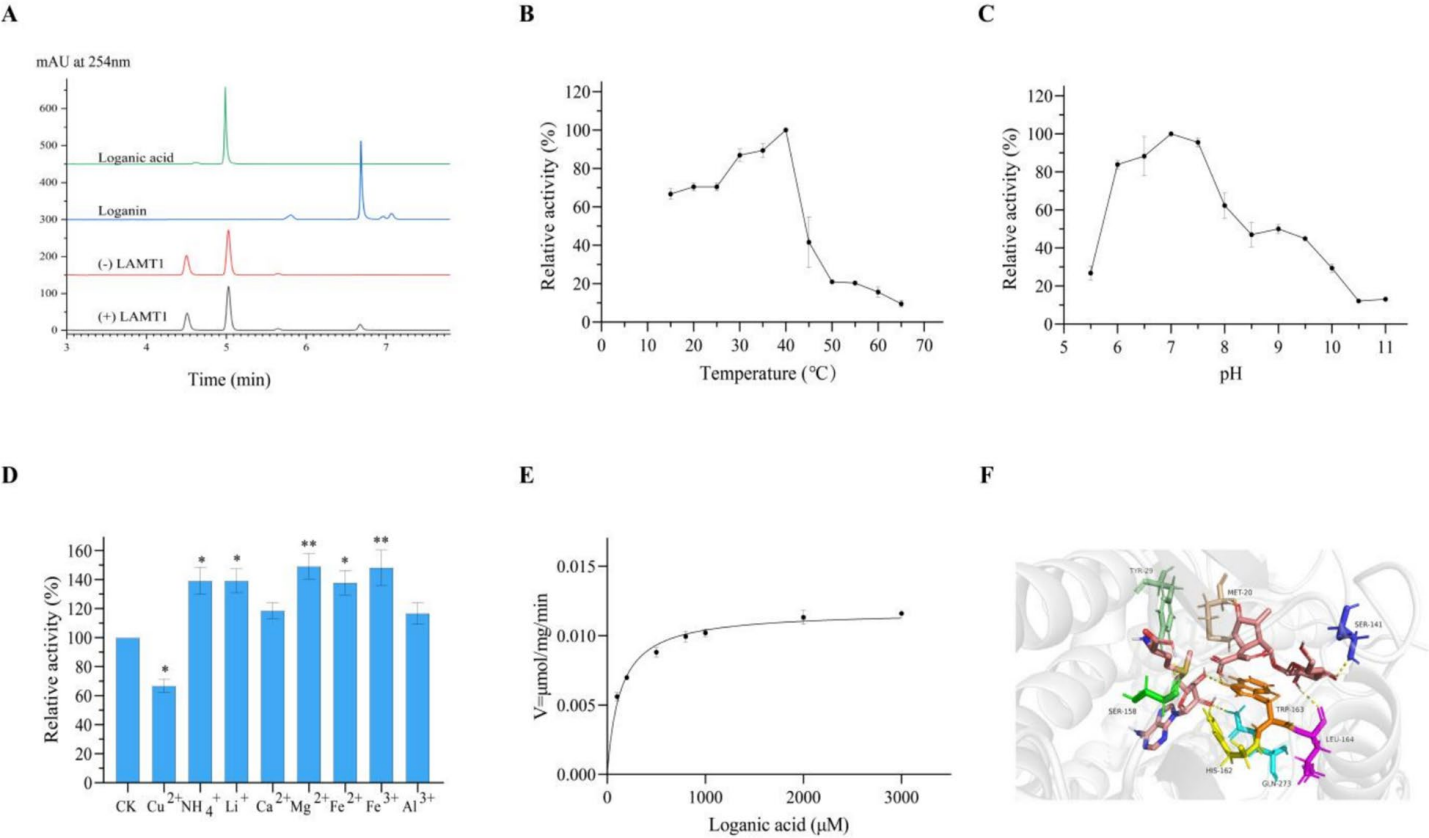
The enzyme activity assay of UrLAMT1. **A** HPLC detection showed that the enzymatic reaction yielded the presence of Loganin in the reaction mixture catalyzed by UrLAMT1. **B** The impact of temperature (15–65 °C) on the catalytic reaction was assessed, revealing that the activity towards tryptamine was maximized at 40 °C.** C** A range of buffers spanning pH from 5.5 to 11.0 was employed to investigate the impact of pH on the enzymatic activity of the purified recombinant UrLAMT1. The enzymatic activity towards tryptamine reached its maximum at pH 7.0. **D** The impact of metal ions on the activity of the UrLAMT1 enzyme. * represents *p* < 0.05; ** represents *p* < 0.01. **E** Enzyme kinetics of UrLAMT1 were determined using the Michaelis–Menten equation. **F** Docking results for Loganate of UrLAMT1

**Fig. 7:**
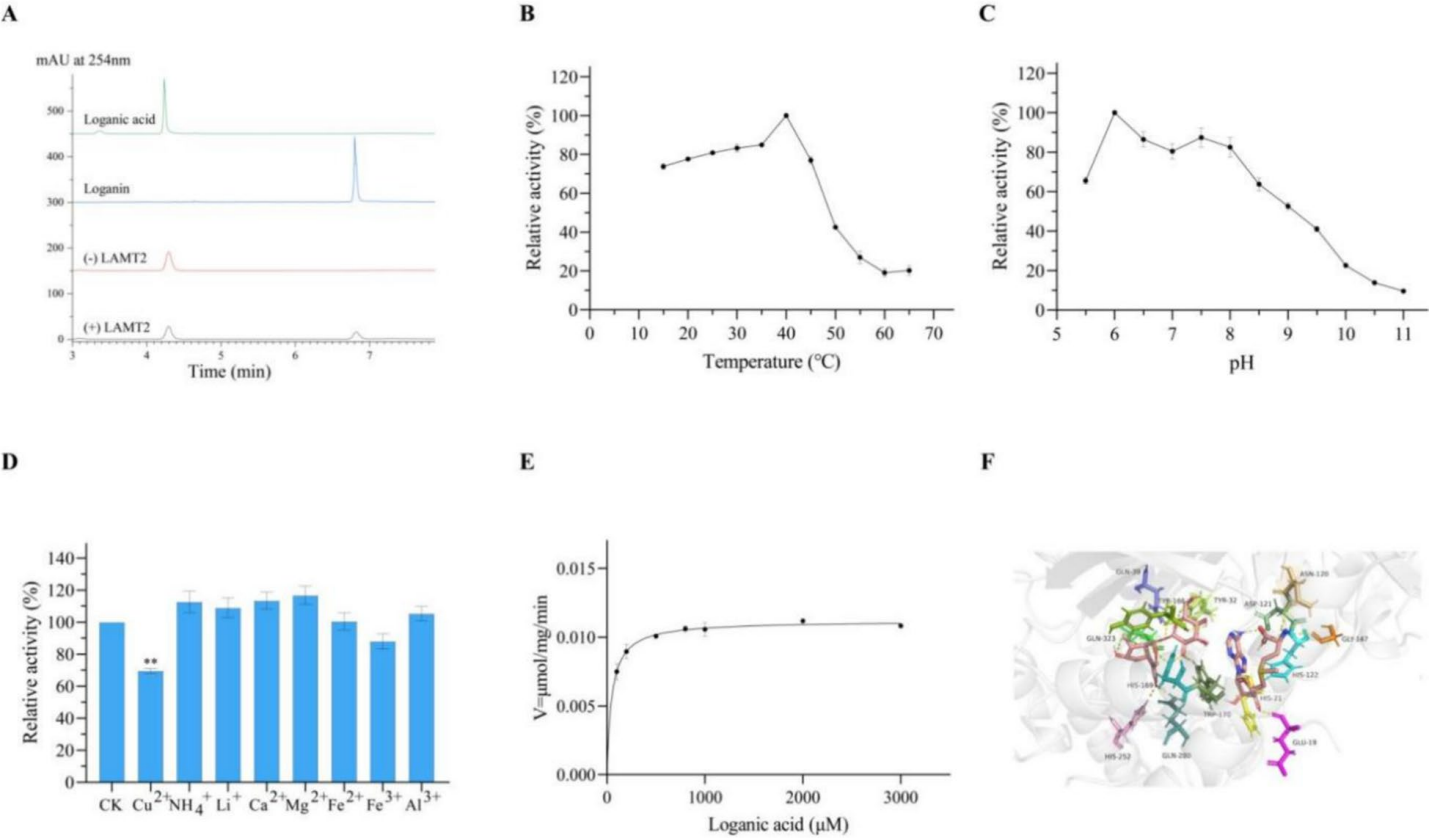
The enzyme activity assay of UrLAMT2. **A** HPLC detection showed that the enzymatic reaction yielded the presence of Loganin in the reaction mixture catalyzed by UrLAMT2. **B** The impact of temperature (15–65 °C) on the catalytic reaction was assessed, revealing that the activity towards tryptamine was maximized at 40 °C.** C** A range of buffers spanning pH from 5.5 to 11.0 was employed to investigate the impact of pH on the enzymatic activity of the purified recombinant UrLAMT2. The enzymatic activity towards tryptamine reached its maximum at pH 6.0. **D** The impact of metal ions on the activity of the UrLAMT2 enzyme. ** represents *p* < 0.01. **E** Enzyme kinetics of UrLAMT2 were determined using the Michaelis–Menten equation. **F** Docking results for Loganate of UrLAMT2

The sequence of *UrLAMT1* is 1116 bp and encodes 371 amino acids, with a predicted molecular weight of 41.97 kDa. The theoretical pI, instability index and GRAVY were predicted to be 6.08, 39.73 and −0.30, respectively. The sequence of *UrLAMT2* is 1137 bp and encodes 378 amino acids, with a predicted molecular weight of 42.61 kDa. The theoretical pI, instability index and GRAVY were predicted to be 5.76, 37.30 and −0.358, respectively. The results of multiple sequence alignment of UrLAMT1 and UrLAMT2 with LAMT proteins from other species revealed that UrLAMT1 and UrLAMT2 all had a loganate binding site, indicating that they might convert loganate to loganin (Fig. S12). According to molecular docking experiments, UrLAMT1 residue Trp-163 was likely responsible for engaging and orienting the carboxylate group of loganate to facilitate methyl transfer. The cavity of UrLAMT1 contains multiple polar residues (Met‐20, Ser‐141, and Leu-164) positioned within the hydrogen‐bonding range of the substrate loganate (Fig. 6F). In addition, residue Gln-280 is responsible for engaging and orienting the carboxylate group of loganate to facilitate methyl transfer. The cavity of UrLAMT2 also contains multiple polar residues (Tyr32, Gln39, Tyr166, His169, Trp170, His252, Gln280, and Gln323) (Fig. 7F).

Enzymatic assays revealed that both UrLAMT1 and UrLAMT2 can catalyse the conversion of loganate to loganin (Fig. 6A, Fig. 7A). Recombinant UrLAMT1 with a TF‐tag and UrLAMT2 with a His-tag were expressed and purified under optimized conditions (Fig. S13). For both UrLAMT1 and UrLAMT2, the optimal reaction temperature was 40 °C. For UrLAMT1, the optimal pH for the reaction was 7.0, whereas for UrLAMT2, the most ideal pH for the loganate catalytic reaction was 6.0 (Fig. 6B, C; Fig. 7B, C). In addition, the recombinant UrLAMT1 and UrLAMT2 proteins were strongly inhibited by the metal ion Cu^2+^, whereas NH_4_^+^ and Li^+^ promoted activity (Fig. 6D, Fig. 7D). LAMT2 demonstrates superior substrate affinity (*K*_m_ = 49.77 μM vs. LAMT1’s 131 μM), making it more efficient in low-substrate environments. Despite LAMT1’s ~ 2.2-fold faster turnover number (*k*_cat_ = 0.0176 s^−1^ vs. 0.0081 s^−1^), LAMT2 achieves higher overall catalytic efficiency (162.67 vs. 134.62 s^−1.^M^−1^) due to its strong binding capability. Thus, LAMT2 prioritizes substrate conservation, while LAMT1 favors rapid turnover under abundant substrate conditions.

### Identification of *UrWRKY* gene family members in the *U. rhynchophylla* genome

Next, we sought to further study the transcriptional regulation of RIN biosynthesis in *U. rhynchophylla*. Many TF families, such as bHLH, MYB, WRKY, NAC, HD-ZIP, TCP, and GATA (Dataset2), have been identified in the *U. rhynchophylla* genome. Some WRKY TFs function in the regulation of secondary metabolic pathways. Seventy-two *UrWRKY* genes were identified in the *U. rhynchophylla* genome. Based on their positions on the *U. rhynchophylla* chromosomes, these genes were named *UrWRKY1*-*72*. To characterize these UrWRKYs further, the amino acid lengths, molecular weights (MWs), theoretical pIs, instability indices, and subcellular localizations were analysed (Table S12). The prediction of subcellular localization indicated that the vast majority of UrWRKYs were located in the nucleus, whereas UrWRKY3 was predicted to be found in the chloroplast. For these 72 UrWRKY proteins, UrWRKY55 had the greatest amino acid length (776 aa), and UrWRKY46 had the shortest (153 aa); the proteins varied from 17.23 kDa (UrWRKY46) to 85.11 kDa (UrWRKY55), with an overall average of 43.27 kDa. The pIs ranged from 4.96 (UrWRKY46) to 9.63 (UrWRKY25). The majority of the UrWRKY proteins were considered unstable, with the exceptions of UrWRKY3, UrWRKY8, and UrWRKY36.

### Phylogenetic relationships, categorization, and multiple sequence alignment

**Fig. 8:**
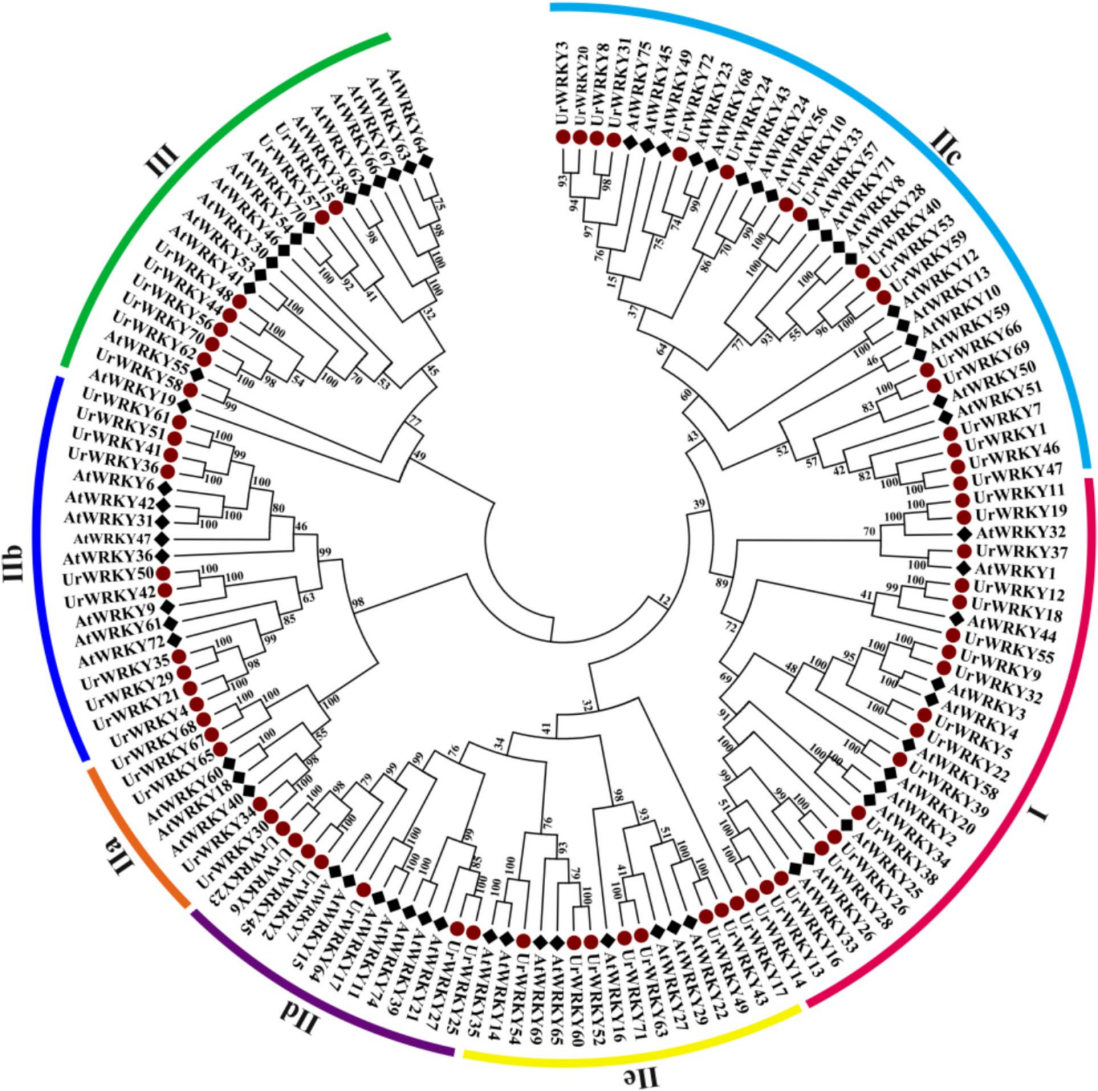
The phylogenetic tree of WRKY TFs from *A. thaliana* and *U. rhynchophylla*. The phylogenetic tree was constructed using MEGA 7.0 software with neighbor-joining method and 1000 bootstrap replicates. The WRKY TFs from different groups are marked with different colors

To analyse the potential evolutionary relationships of the UrWRKYs, a phylogenetic tree for the 72 UrWRKY proteins and 70 AtWRKY proteins was constructed. As shown in Fig. 8, the 72 UrWRKY proteins were classified into three major groups (Group I, Group II, and Group III) on the basis of the features of AtWRKY (Kang et al. 2021). Seventeen UrWRKYs were classified into Group I, and 47 UrWRKYs belonged to Group II and were further distributed into five subgroups: IIa (*n* = 5), IIb (*n* = 10), IIc (*n* = 17), IId (*n* = 7), and IIe (*n* = 8). The remaining 8 UrWRKYs were classified into Group III.

Multiple sequence alignments were used to identify the structural characteristics of the UrWRKY proteins using MEGA 7.0 software. As shown in Figure S14, the multiple sequence alignments indicated that the 17 UrWRKYs (17/72, 23.6%) in Group I contained two complete conserved domains, including the highly conserved WRKYGQK sequence and C_2_H_2_ zinc-finger motifs. The 47 (47/72, 65.2%) UrWRKYs that belonged to Group II contained one complete conserved domain (WRKYGQK) and one zinc-finger motif; however, 6 UrWRKYs belonging to Group IIc had variant conserved domains, such as the WRKYGKK sequence.

### Analysis of conserved motifs, gene structure, and *cis*-acting elements

To better understand the similarity and diversity of the motifs among the different UrWRKY proteins, eight motifs ranging from 20 to 50 aa were identified in the UrWRKY protein sequences (Fig. S15A). As shown in Fig. S15B, C, the UrWRKY family members that had similar motifs were classified into the same groups. Motifs 1 and 2 were found in all 72 UrWRKY proteins, corresponding to the conserved WRKY domain. Motifs 3, 4, and 8 were unique to Group I. Motifs 6 and 7 were found mainly in Groups IIa and IIb. Motif 5 was found in Group I, Group IIb, and Group IIc. This implies that proteins belonging to the same group have similar motifs and therefore may perform similar functions. To further study whether the diversity of gene structure might have promoted the evolution of the *WRKY* gene family, gene structure analysis of the *UrWRKY* genes was performed. The number of exons in the *UrWRKY* genes varied from two to ten. Most *UrWRKY* genes (44.4%) had three exons with two introns (Fig. S15D), eighteen members had five exons, eight had six exons, and seven had four exons. *UrWRKY* genes with similar structures were classified together, as the Group IId, IIe and Group III *UrWRKY* genes mostly contained three exons and two introns. The Group I and Group IIb IIa genes contained 3–7 exons. The Group IIc genes contained 2–10 exons, whereas the Group IId, IIe, and Group III genes contained 3 exons, with the only exception being *UrWRKY56*, with 2 exons. *UrWRKY6* and *UrWRKY23* contained four exons.

To further elucidate the potential regulation of WRKY TFs in *U. rhynchophylla*, the *cis*-acting promoter elements were verified via PlantCARE software using the 2.0 kb region upstream of the gene. The results revealed that there was more than one *cis*-acting element in the promoter regions of the *UrWRKY* genes. Motifs related to growth and development (834), phytohormone response (445), and abiotic and biotic stress (284) were found in the promoter regions of the *UrWRKY* genes (Fig. S15E). Many phytohormone-responsive elements, such as MeJA (124), abscisic acid (AbA) (48), salicylic acid (SA) (54), auxin (16), and gibberellin (GA) (13), were found in the promoter regions of the *UrWRKY* genes. Moreover, elements related to abiotic and biotic responses, e.g., anaerobic induction (129), drought (60), low temperature (36), and wound induction (7), were detected. Furthermore, 12 Sp1 elements, 13 lamp elements, and 17 circadian elements were also found and are potentially related to plant growth and development. These results imply that the *UrWRKY* genes are involved in many diverse plant functions.

### Chromosomal location, gene duplication and collinearity analysis

To further extend our analysis of the evolution of the *UrWRKY* gene family members, the chromosomal locations of the *UrWRKY* genes were determined. A total of 72 *UrWRKY* genes were found to map unevenly to the 22 chromosomes of *U. rhynchophylla* (Fig. S16A). Chromosome 18 had the greatest number of *UrWRKY* genes (6 genes), followed by chromosomes 2, 4, 17, 20, and 21 (5 genes), with no genes on chromosome 19. Gene duplication event analysis was performed to identify possible expansions of the *UrWRKY* gene family. A total of 50 gene pairs among 49 *WRKY* genes were regarded as segmental duplications and were distributed on different chromosomes, especially those located between chromosomes 2 and 15. Only one pair, *UrWRKY57* and *UrWRKY58*, was identified as a tandem duplication gene pair located on chromosome 18 (Fig. S16A). These results indicate that segmental and tandem publications in these regions might have contributed to the expansion of the *UrWRKY* gene family.

To gain insight into the origin and evolutionary history of the *UrWRKY* gene family, three collinear graphs of *U. rhynchophylla* were constructed with three representative species, one monocotyledon (*O. sativa*) and two dicotyledons (*A. thaliana* and *C. canephora*) (Fig. S16B, C, D). A total of 60 *UrWRKY* genes exhibited collinear relationships with those in *C. canephora*, followed by *A. thaliana* (50) and *O. sativa* (28) (Table S13). There were 94, 93, and 46 orthologous gene pairs with these other three species, respectively. A total of 27 *UrWRKY* gene pairs were identified in these other species, indicating that these gene pairs are likely to have formed earlier than the formation of common ancestral species and may play a key role in regulating the evolution of the *UrWRKY* genes.

### Selection of the candidate gene *UrWRKY37*

**Fig. 9:**
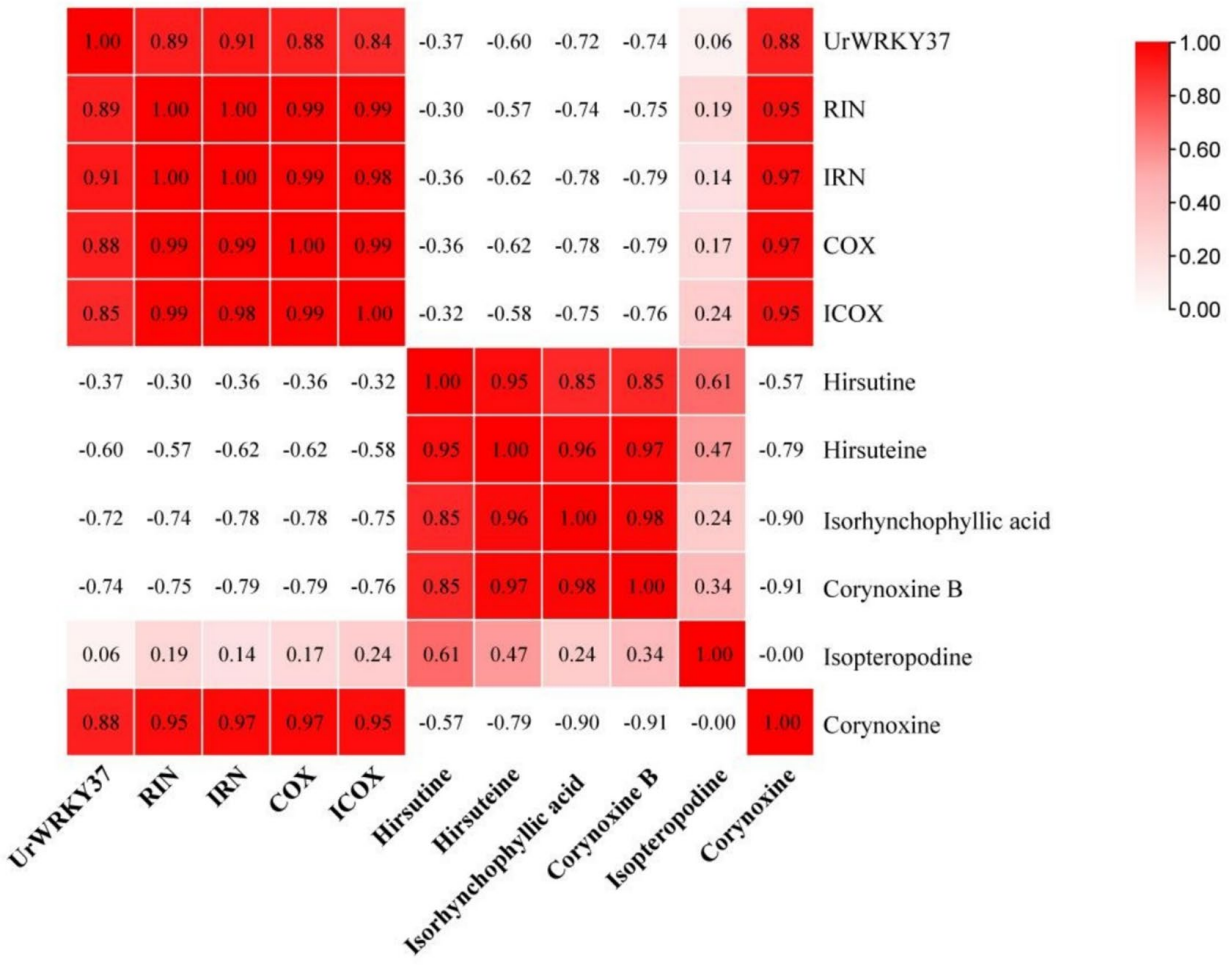
Correlation between the content of MIAs and expression level of *UrWRKY37*. The darker the color, the higher the correlation coefficient

Previous studies have shown that the expression of *UrWRKY37* is upregulated after treatment with MeJA and that the expression patterns of this gene correspond to those of *UrTDC* and *UrSGD* (Gattuso et al. 2004). UrWRKY37 was also found to have the highest homology with CrWRKY1 (GenBank: ADT82685.1) in the *UrWRKY* gene family, and the overexpression of CrWRKY1 resulted in the upregulation of key enzyme-encoding genes in the MIA pathways and an increase in the accumulation of effective ingredients (Chen et al. 2022). Moreover, the expression level of WRKY37 was significantly positively correlated with the content of most MIAs (Fig. 9), indicating that WRKY37 is likely to regulate the RIN biosynthetic pathway. Therefore, we speculated that UrWRKY37 might have similar functions, and UrWRKY37 was therefore selected for further in-depth analysis.

#### In vitro validation of UrWRKY37

The *UrWRKY37* open reading frame (ORF) is 1689 bp in length and encodes 562 amino acids. Protein sequence comparison revealed that UrWRKY37 shares 66.73%, 56.77%, 56.00%, and 55.34% identity with CaWRKY1 (XP_027062250.1), NtWRKY1 (AAD16138.1), SvWRKY1 (XP_049359053.1), and StWRKY1 (XP_006352011.1), respectively. According to the phylogenetic tree analysis, UrWRKY37 clustered with AtWRKY1, which belongs to Group I (Fig. 8) and contains two conserved WRKYGQK domains and C_2_H_2_ zinc-finger motifs (Fig. S14).

**Fig. 10:**
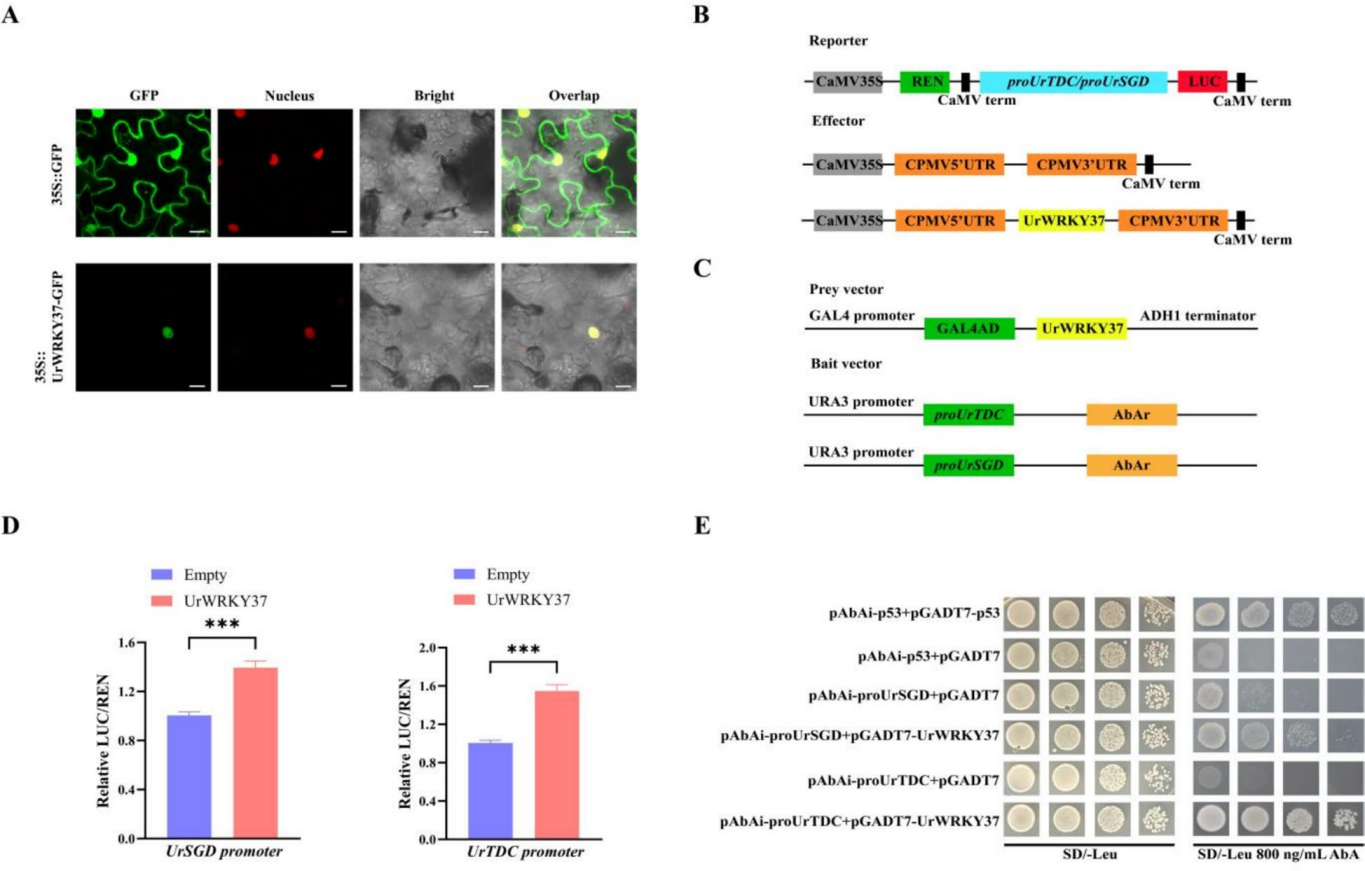
The in-vitro validation of UrWRKY37. **A** The subcellular localization assay of UrWRKY37 in 21 days *N. benthamiana* leaves cell. 35S::GFP and 35S::UrWRKY37-GFP were transiently expressed in tobacco leaves. The GFP fuorescence was observed via confocal microscopy. Scale bars = 10 μm. **B**, **C** Schematic diagram of recombinant plasmids used in the Dual-LUC assay and Y1H assay. **D** Activation of *UrTDC* and *UrSGD* promoter by UrWRKY37 in Dual-LUC assays. *** represents* p* < 0.001. E. Yeast one-hybrid assays of the interactions between the UrWRKY37 and the *UrTDC* and *UrSGD* promoters

The subcellular localization of UrWRKY37 was verified by co-transforming the pBI121-UrWRKY37-EGFP fusion plasmid and the nuclear marker mCherry-RFP into *N. benthamiana* leaf cells. The pBI121-UrWRKY37-EGFP fusion protein displayed fluorescence only in the nucleus of *N. benthamiana* leaf cells and colocalized with the nuclear marker. These results revealed that UrWRKY37 is a nuclear-localized protein (Fig. 10A).

Dual-LUC assays were used to determine whether UrWRKY37 can activate *proUrTDC* and *proUrSGD*. The promoter regions of *UrTDC* and *UrSGD* were separately inserted into pGreen0800-LUC, which was used to drive the luciferase tag as reporters. In addition, UrWRKY37 was inserted into pGreen62-SK as an effector (Fig. 10B, C). UrWRKY37 activated *proUrTDC* and *proUrSGD* compared with the control according to the LUC/REN activity ratio (Fig. 10D). To further verify that UrWRKY37 can bind fully to the W-box on the promoter of *UrTDC* and *UrSGD*, Y1H assays were employed.

Six concentrations of AbA (0, 100, 200, 500, 800, and 1000 ng/mL) were added to the SD/-Ura medium to verify the self-activation of *proUrTDC* and *proUrSGD*. Finally, the lowest AbA inhibition concentration obtained through the self-activation experiment was 800 ng/mL. Next, the prey vector AD-UrWRKY37 was transformed into the Y1H Gold strain containing *proUrTDC* and *proUrSGD*. When 800 ng/mL AbA was added to the medium, the strain co-expressing BD-*proUrTDC*/*proUrSGD* and UrWRKY37 and the positive control grew normally on SD/-Leu/AbA^800^ medium, but the negative control did not grow (Fig. 10E).

#### OE-*UrWRKY37* increased alkaloid production in *U. rhynchophylla* hairy roots

**Fig. 11:**
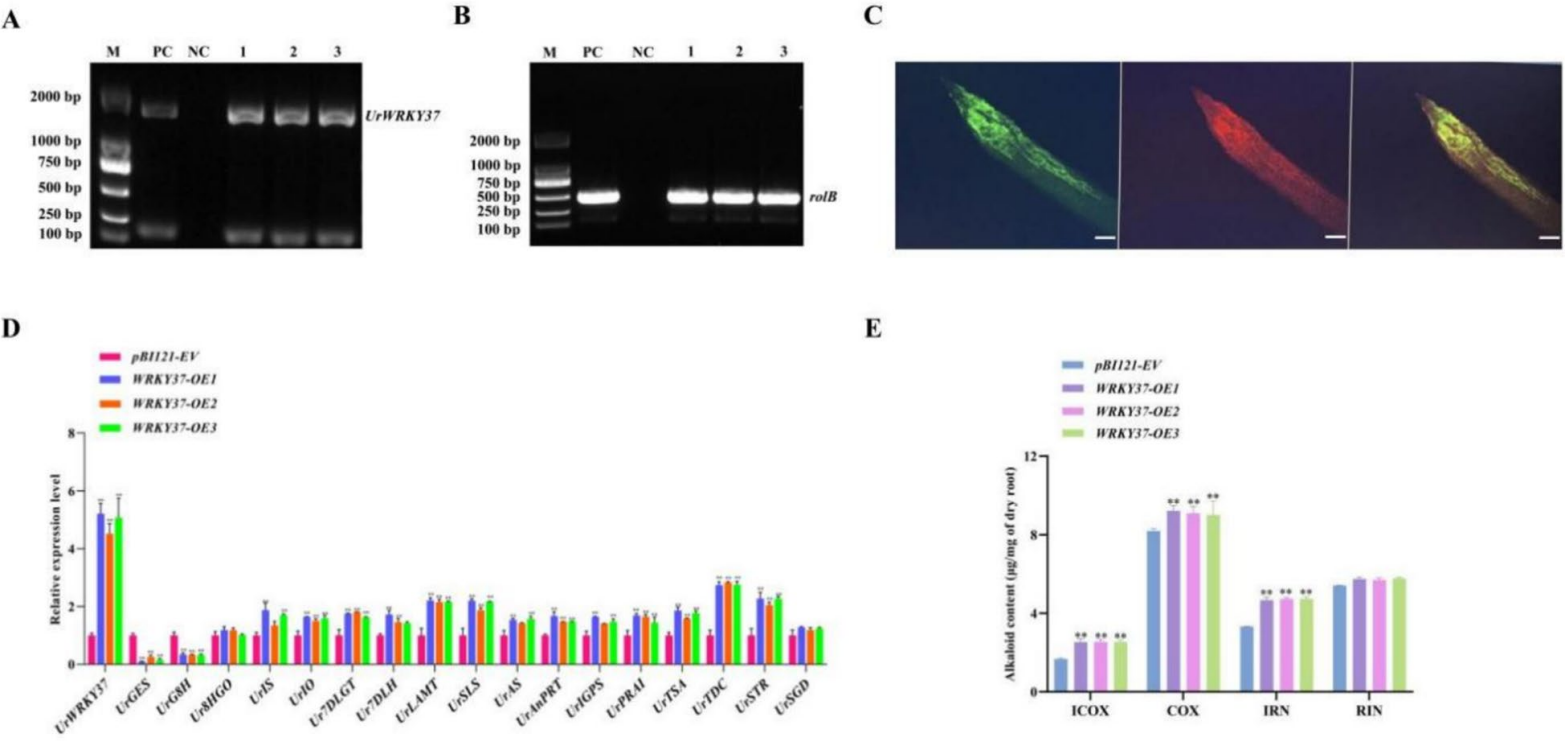
Overexpression of *UrWRKY37* in *U. rhynchophylla* hairy roots. **A**,** B** Identification of positive *UrWRKY37-OE* hairy roots by PCR. **C** Fluorescence assay of regenerated hairy roots. Green fluorescent protein (GFP). Autofluorescence (Chl). Merged images were simultaneously exhibited, bars = 100 μm. **D** The expression levels of *U. rhynchophylla* biosynthesis genes in the *UrWRKY37-OE* hairy roots. * represents *p* < 0.05; ** represents *p* < 0.01. **E** The content of four kinds of alkaloids in *U. rhynchophylla*. ** represents *p* < 0.01

*UrWRKY37* was overexpressed (OE) in transgenic hairy roots to determine whether it altered MIA biosynthesis in *U. rhynchophylla*. Positive transgenic hairy roots were examined by PCR and fluorescence microscopy (Fig. 11 A and B). The fluorescence signals of the hairy roots were observed under a fluorescence microscope (Zeiss, Germany), and a 0.5 cm long tip of the hairy root was excised. The GFP fluorescence signals were observed at 528 nm, and autofluorescence was monitored at 548 nm. All the fluorescence tests were carried out more than 3 times. The GFP signal was strongly expressed in the hairy roots of the transgenic plants. In addition, the UrWRKY37 expression level was 5.2-fold greater in the OE-*UrWRKY37* transgenic roots than in the pBI121 empty vector (EV) control transgenic roots (Fig. 11D). There was no significant difference in phenotype between the OE-*UrWRKY37* transgenic roots and control roots (Fig. S17). The HPLC results indicated elevated alkaloid contents in the OE-*UrWRKY37* transgenic hairy roots (Fig. 11E). Among the four different alkaloids detected, the control hairy roots produced 1.68 ± 0.045, 8.16 ± 0.16, 3.33 ± 0.018, and 5.41 ± 0.014 μg/mg dry roots of isocorynoxeine, corynoxeine, isorhynchophylline, and rhynchophylline, respectively. Compared with those of the control, the OE-*UrWRKY37* transgenic hairy roots presented significant increases in the levels of isocorynoxeine (2.54 ± 0.12), corynoxeine (9.1 ± 0.42), and isorhynchophylline (4.71 ± 0.10).

The relative expression levels of 17 genes in the *U. rhynchophylla* biosynthetic pathway were analysed in the hairy roots of the transgenic plants (Fig. 11D). The expression of most pathway genes was upregulated, apart from *UrGES* and *UrG8H*. Among these genes, the most significantly upregulated was the *UrTDC* gene, whose expression increased from 2.7- to 2.9-fold in the hairy roots of the transgenic plants compared with those of the control plants. Other genes whose expression increased were *UrSTR* (2.04- to 2.27-fold), *UrSLS* (1.87- to 2.2-fold), and *UrLAMT* (2.14- to 2.20-fold). The overexpression of *UrWRKY37* significantly reduced the expression of *UrGES* (4- to 11-fold) and *UrG8H* (3.0- to 3.2–fold) but did not markedly affect the expression of other genes, such as *UrSGD* and *Ur8HGO*.

## Discussion

### The first chromosomal-level *U. rhynchophylla* genome was assembled

*U. rhynchophylla* is an important plant used in traditional Chinese medicine and is a source of important medicinal alkaloids that have pharmacological significance (Suttipanta et al. 2011). The main medicinal ingredients of *U. rhynchophylla* are located in hook-bearing stems, which are processed by drying. In recent decades, nearly 129 alkaloids have been extracted and identified from *Uncaria* species (Zhang et al. 2020); among them, RIN has been found to effectively limit the signalling pathways associated with degenerative diseases, such as AD (Pan et al. 2015); IRN has been shown to have therapeutic effects on cardiovascular diseases and central nervous system disorders, such as hypertension (Kaneko et al. 2020); and corynoxine B can effectively alleviate Parkinson's disease (Li et al. 2024). Although these MIAs have many therapeutic properties, these alkaloids are present in low amounts (approximately 0.2%, w/w), which restricts their widespread use; thus, finding an economically and environmentally responsible way to increase their production is vital. Due to the lack of high-quality genome resources, it has not been possible to identify all the genes associated with MIAs. This knowledge gap hinders a comprehensive understanding of MIA biosynthetic pathways and limits the potential for green and efficient production through synthetic biology approaches (Zhu et al. 2023). With the development of third-generation high-throughput sequencing (ONT), which enables cost-efficient long-read sequencing (Chen et al., 2023), many medicinal plants now have completely assembled genomes (Cao et al. 2018; Hoff et al. 2019; Mahajan et al. 2023; Szadkowski et al. 2023). In this study, we performed chromosome karyotype analysis (2n = 2x = 44 = 18 m + 26Sm (2SAT)) in *U. rhynchophylla.* Our genome survey, revealed the *U. rhynchophylla* genome to have high heterozygosity (1.07%) and a large portion of repeat sequences (52.2%), posing significant challenges for genome assembly. However, through a combination of ONT, Illumina NovaSeq, and Hi-C sequencing, we successfully constructed an excellent genome assembly of 627.70 Mb, with a contig N50 of 1.83 Mb. It is of high quality, as demonstrated by the high gene completeness in the BUSCO analysis (96.1%) (Wang et al., 2021). Hi-C is essential for improving the sequence continuity of our assembly, which enables the mapping of genes to chromosomes (Manni et al. 2021). Nearly 94.46% of the contigs were mapped onto the 22 chromosomes, indicating a high incorporation rate in the genome assembly. Therefore, our chromosomal-level assembly of *U. rhynchophylla* fills critical gaps in the *Uncaria* genome, enabling the first comprehensive genome-wide analysis of this species.

The genomes of many Chinese medicinal plants containing MIAs, such as *G. elegans* (335.13 Mb) (Kang et al. 2021), *O. pumila* (456.9 Mb) (You et al. 2021), and *C. roseus* (561.7 Mb) (Kadota et al*.*, 2020), have been completely sequenced. The genome size of *U. rhynchophylla* is larger than those of these three species. Evolution-driven changes in the size of gene families are natural occurrences that offer selective benefits and contribute to the diversity in organization and regulation in various organisms (Zhang et al., 2019b). The number of repeated sequences in the *U. rhynchophylla* genome was greater than 51.19%, with the most prevalent type (67.1%) being TEs. This percentage is greater than that of the *G. elegans* genome (43.16%) (Kang et al. 2021). In addition, LTRs were the major TEs in the repetitive sequences, accounting for 34.35% of the TEs (Table S8). Research has previously shown that a high number of LTRs, with different ratios of LTR subgroups (*Gypsy* and *Copia*), is a contributing factor to the expansion of genome size (Xu et al. 2023). Interestingly, in our study, *Gypsy* elements accounted for 22.77% of the LTRs, whereas *Copia* elements accounted for only 8.88%. This result provides a potential explanation for the larger genome of *U. rhynchophylla*, attributed mainly to the number of *Copia* retrotransposons, than that of the other three MIA-producing plants. This situation also seems to have occurred in *Polygonum cuspidatum* (Morgante et al. 2007).

A phylogenetic tree was constructed based on 142 gene families that are shared by all species as single-copy orthologous gene families (Fig. S3). An ancient whole-genome duplication event in *Uncaria* was identified, possibly because of the high natural selection pressure to enrich DNA elements (Demuth and Hahn 2009), via comparison with three closely related Rubiaceae plants, *U. rhynchophylla*, *C. arabica*, and *G. jasminoides*. In this study, the 2020 gene families were specific to *U. rhynchophylla*; interestingly, the unique gene families were enriched in GO:0009820 (indole alkaloid metabolic process), which might influence the accumulation of bioactive ingredients in this important Chinese herb (Ludwig et al. 2021). Overall, our *U. rhynchophylla* genome provides a valuable genetic resource for promoting the analysis of biosynthetic pathways and research on molecular regulation.

### Biosynthetic pathway analysis and transcriptional regulation in *U. rhynchophylla*

Many medicinal plants and their bioactive ingredients have attracted increasing attention because of their potential to become natural drugs for treating several diseases (Li et al. 2022). Alkaloids are known to constitute one of the largest types of natural drugs (Csikós et al. 2021). In our study, the contents of ten target alkaloids in different tissues were detected, with the highest contents of five alkaloids found in the stem hook, which corresponds to the main part of the plant used medicinally (Olofinsan et al. 2023). The enzymes in the RIN and IRN biosynthetic pathways, which are encoded by multiple genes, catalyse secondary metabolites. The genes identified in the genome assembly, transcriptome, and metabolite pathways increase the resources available for studying transcriptional regulation and the functional properties of secondary metabolites.

In this study, two categories of genes encoding three key enzymes were identified from the genome data. Tryptophan can be decarboxylated to form tryptamine via catalysis by the cytosolic enzyme TDC (Zhang et al. 2024). TDC, a member of the aromatic class of L-amino acid decarboxylases (AADCs), shares similarities with PLP-dependent enzymes. To date, the enzyme TDC has been identified in many MIA-producing plants, such as *O. pumila* (Kang et al. 2021), *Rauvolfia verticillata* (Qu et al. 2019), and *C. roseus* (Wassenberg et al. 2017), and is considered a key limiting-rate enzyme in MIA biosynthetic pathways. Thus, TDC is essential for the conversion pathway of tryptamine derivative biosynthesis in plants. In this study, we first demonstrated that UrTDC6 converts tryptophan into tryptamine in *U. rhynchophylla*. The results of the enzymatic property analyses revealed that the ideal reaction pH was 7.0, and the ideal reaction temperature was 50 °C; in comparison, the values determined for the TDC of *O. sativa* were 45 °C and pH 7.5–8.5 (Noé et al. 1984) and *O. pumila* (temperature: 50 °C; pH: 8) (Kang et al. 2021), suggesting that UrTDC is an acidic protein. Moreover, the metal ion Cu^2+^ strongly inhibited UrTDC6 activity, possibly because Cu^2+^ might inhibit UrTDC6 activity by binding to PLP. Molecular autodocking results revealed that UrTDC6 has five highly conserved sites: Thr263, Thr370, Phe125, His319, and Phe102. These findings suggest that the UrTDC6 protein is a member of the AAAD plant family (Kang et al. 2007). LAMT catalyses loganin to loganate through the addition of methyl groups in medicinal plants and uses strictosidine as an intermediate, such as in *U. rhynchophylla* (Torrens-Spence et al. 2020). In this study, we first identified two UrLAMT enzymes, UrLAMT1 and UrLAMT2. UrLAMT1 and UrLAMT2 can catalyse the conversion of loganin into loganate (Figs. 5, and 6). This result is opposite to that of the LAMT of *O. pumila*. In *O. pumila*, only OpLAMT1 and not OpLAMT2 has a catalytic function (You et al. 2021). Notably, however, the *K*_m_ of UrLAMT2 was lower than that of UrLAMT1, indicating that UrLAMT2 is superior to UrLAMT1 in terms of its substrate affinity for loganin (Guo et al. 2023). This finding is in line with the accumulation of RIN and IRN, the key enzyme-encoding genes in the biosynthetic pathway that are also tissue specific, with relatively high expression levels in the stem hooks and relatively low expression in the roots. The two key enzyme-encoding genes *UrLAMT1* and *UrLAMT2* were more highly expressed in the leaves than in the roots and stem hooks, which is inconsistent with MIA accumulation. Loganate accumulated mainly in the leaves; thus, the expression of *UrLAMT1* and *UrLAMT2* was consistent with the accumulation of loganate but not that of RIN or IRN. Therefore, when screening for key enzyme-encoding genes, selecting only genes that are consistent with changes in the final product content is insufficient. At present, genetic engineering of biosynthetic enzymes can be used to investigate and increase the accumulation of MIAs in *U. rhynchophylla* and *C. roses*. For example, overexpression of *OpG10H* and *OpSTR* increased the accumulation of camptothecin in *O. pumila* (Horikoshi et al. 2022). Two *UrSTR* genes, *UrSTR1* and *UrSTR5*, were identified in *U. rhynchophylla*, with overexpression of *UrSTR1* and *UrSTR5* found to increase the content of MIAs through transgenic hairy root analysis, and RNAi downregulation of *UrSTR1* and *UrSTR5* significantly decreased the accumulation of MIAs in *U. rhychophylla* (Kulhar and Rajakumara 2023). However, no other functional genes have been found to increase the MIA content of *U. rhynchophylla* through this method. In subsequent studies, experiments using genetic engineering will be performed to explore whether UrTDC6, UrLAMT1, and UrLAMT2 can increase the content of UR-MIAs.

Transcriptional regulation leads to spatiotemporal expression of biosynthetic enzyme-encoding genes via the binding of DNA-binding domains in the protein sequences of transcription factors to *cis*-acting elements in the upstream promoter regions of target genes (Cui et al. 2015). Hormones and transgenic technologies are important methods for regulating TFs because they can control the transcription of biosynthetic enzymes, effectively altering secondary metabolite biosynthesis in plants (Ludwig et al. 2021; Patra et al., 2013). Currently, genes involved in the MIA biosynthetic pathway are known to be regulated by various transcription factors, such as bHLH, MYB, WRKY, NAC, and GATA family members. In *C. roseus*, CrbHLH3 can bind to the E-box in the *CrG10H* promoter, activating its expression in tobacco cells, and the overexpression of *CrbHLH3* in flower petals considerably increased the accumulation of loganate and upregulated the expression of genes involved in iridoid biosynthesis (Eilert et al. 1987). CrWRKY1 was found to bind to the W-box of the *CrTDC* promoter region via in vitro validation experiments, and the overexpression of *CrWRKY1* significantly upregulated the expression of *CrTDC* and increased the tryptamine concentration in transgenic hairy roots (Chen et al. 2022). In *O. pumila*, OpNAC1 can suppress the expression of *OpLAMT1* in transgenic hairy roots (You et al. 2021). Transient overexpression of *CrGATA1* in *C. roses* seedlings led to upregulated expression of genes in the vindoline pathway and the accumulation of vindoline, whereas gene silencing of *CrGATA1* in *C. roses* significantly decreased gene expression and the accumulation of vindoline (Singh et al. 2021).

**Fig. 12:**
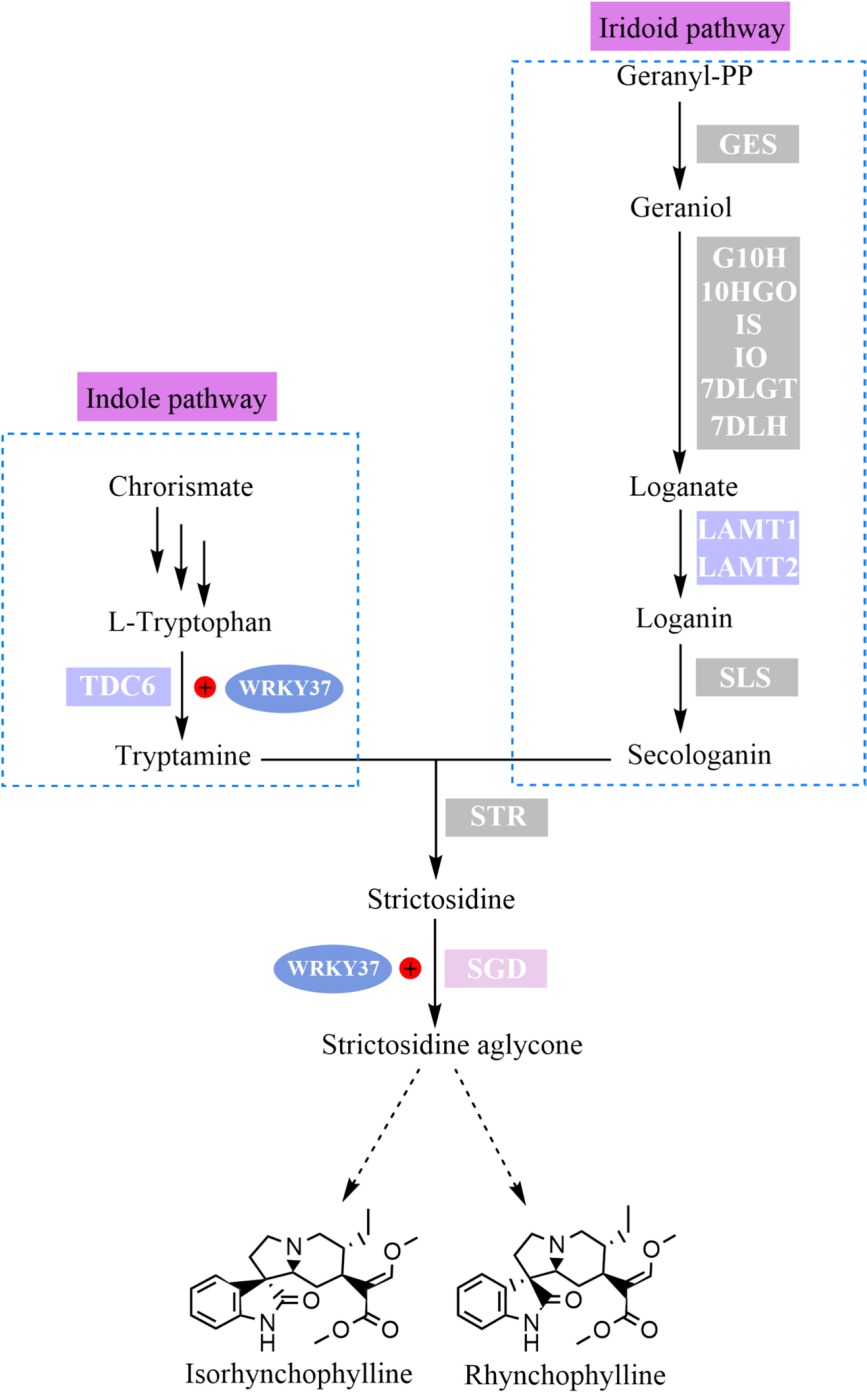
Model diagram of key enzyme genes and transcription factor UrWRKY37 regulatory network in UR-MIAs biosynthesis pathway. The pink rectangle represents the key enzyme genes interaction with transcription factors in this study. The purple rectangle represents the key enzyme genes that have had their catalyze function validated in this study. The red circle represents positive regulation

The WRKY transcription factor gene family is one of the largest TF families and is associated with the response to different biological, abiotic and hormone signalling pathways; members of this family interact with a variety of proteins to regulate their functions in different signal transduction pathways (Liu et al. 2019). Genome-wide analysis of the *WRKY* gene family has been performed in many higher plants, including *A. thaliana* (Jiang et al. 2017), *O. sativa* (Wu et al. 2005), *C. sativus* (Xu et al. 2016), and *O. pumila* (Wu et al*.*, 2023). In this study, we sought to identify the members of the *WRKY* gene family in *U. rhynchophylla*, and 72 *UrWRKY* genes were subsequently analysed by bioinformatic analysis. UrWRKYs can be classified into three major groups based on the number of WRKY domains and the type of zinc-finger motif. As shown in Fig. 8, most UrWRKYs were classified into Group II (47/72, 52.2%), indicating that Group II may have experienced gene duplications during evolution. This result is similar to that reported for *I. indigotica* (Ling et al. 2011) and *Eucommia ulmoides* (Qu et al. 2021). In addition, there are six UrWRKYs (UrWRKY1, UrWRKY7, UrWRKY46, UrWRKY47, UrWRKY66, UrWRKY69) that contain a variant conserved domain, the WRKYGKK sequence, belonging to subgroup IIc. A similar situation has been reported in many other plants, such as *O. sativa* and *M. oleifera* (Liu et al. 2021a). WRKY transcription factors bind to W-box *cis*-acting elements with the help of the WRKY domain (Zhang et al., 2019a). Some studies have shown that the variant WRKY domain might influence normal interactions with target genes; therefore, these six UrWRKY proteins are candidates for further study of their functions and binding activities. The *UrWRKY* genes that had the same conserved motifs and gene structure were classified into the same groups, such as subgroups IIa and IIb. The genes in these groups had a similar conserved motif 6, and interestingly, most genes in subgroups IIe and IId contained three exons and two introns (Fig. S16C, D). These *UrWRKY* genes with similar structures might perform similar functions. Gene duplication events, including tandem, segmental, and whole-genome duplications, are important mechanisms for the acquisition of new genes and gene family expansion in plants (Liu et al. 2021c). In *U. rhynchophylla*, 50 gene pairs among 49 WRKY genes were involved in segmental duplication, and *UrWRKY57* and *UrWRKY58* were identified as tandemly duplicated gene pairs located on chromosome 18. Interestingly, similar results were observed in *I. indigotica* (Ling et al. 2011) and *Saccharum spontaneum* (Magadum et al. 2013), which implies that segmental duplication might play a major role in the expansion of the *UrWRKY* gene family. In addition, collinear maps were constructed with three representative species (*A. thaliana*, *O. sativa*, and *C. canephora*). As shown in Table S13, there were a total of 94 orthologous gene pairs between *U. rhynchophylla* and *C. camphora* and 93 orthologous gene pairs between *U. rhynchophylla* and *A. thaliana*; however, there were only 46 gene pairs (28 genes) between *U. rhynchophylla* and *O. sativa*. This result is consistent with the relationship between *U. rhynchophyll*a and *C. canephora*, as they both belong to the Rubiaceae family, and implies that these orthologous gene pairs might have been generated after the dicot and monocot plants diverged. The tissue-specific expression of *UrWRKY37* corresponded to the contents of RIN and IRN in different tissues. We showed via an in vitro assay that UrWRKY37 can activate *proUrTDC* and *proUrSGD*. The overexpression of *UrWRKY37* in hairy roots resulted in an increase in the levels of four kinds of alkaloids. These results demonstrated that UrWRKY37 regulates genes in the MIA pathways and can increase the RIN and IRN content (Fig. 12). Therefore, UrWRKY37 is an important candidate regulatory gene that requires further examination through gene editing approaches using CRISPR Cas9.

## Materials and methods

### Plant materials

Different tissues of *U. rhynchophylla* (roots, leaves, and stem hooks) were collected from Guangxi Province (Liuzhou, China) in 2020. The leaves of *U. rhynchophylla* were used to isolate DNA for reference genome sequencing. Total RNA was extracted from various tissues of *U. rhynchophylla* for transcriptome sequencing and gene expression analysis. Plantlets of *U. rhynchophylla* cultured under sterile conditions were used for transformation and grown in a plant tissue culture laboratory at 25 °C under 16 h light/8 h dark at Hunan Agriculture University (Hunan, China). Leaves from three-month-old sterile *U. rhynchophylla* plantlets were cut into 1 cm × 1 cm sections and collected for *Agrobacterium*-mediated transformation to regenerate transgenic hairy roots. *Nicotiana benthamiana* was grown for 21 days in a greenhouse at 25 °C under 12 h light/12 h dark light conditions for subcellular localization and dual-LUC.

### Quantitative detection of secondary metabolites via liquid chromatography‒mass spectrometry in *U. rhynchophylla*

The quantitative analysis of ten target MIAs in different tissues (roots, leaves, and stem hooks) of *U. rhynchophylla* was performed via LC‒MS, and four main MIAs (IRN, RIN, COX, and ICOX) in transgenic hairy roots were analysed via high-performance liquid chromatography (HPLC). For the determination of MIA contents in different tissues, the materials were dried at 60 °C, crushed, and screened through a 60 mesh filter. Fifty millilitres of 80% methanol was added to the powder, which was then placed at 50 °C for ultrasonic extraction for 40 min. The supernatant was then filtered through a 0.22 µm organic filter membrane after centrifugation at 12,000 rpm for 15 min, and the liquid was collected in a liquid phase vial. Quantitative analysis was conducted using an Agilent 6530 LC‒MS system (Agilent Technologies, Palo Alto, CA, USA). The separation was carried out on a reversed-phase column (SB C18, 2.1 mm × 100 mm, 1.8 μm, Agilent Technologies) using a solvent system of 0.1% formic acid in water (phase A) and 0.1% formic acid in acetonitrile (phase B). The gradient elution ranged from 10 to 70% phase B over 0–20 min at a flow rate of 0.15 mL/min. The column temperature was maintained at 35 °C, and the sample injection volume was 0.2 μL. Ten standard alkaloid substances at different concentrations were used to construct standard curves for the quantitative analysis of *U. rhynchophylla* alkaloids in various tissues. To determine the contents of the four main alkaloids in the transgenic hairy roots of *U. rhynchophylla*, 0.1 g of powder was added to 500 µL of 80% methanol, followed by ultrasonic extraction for 40 min and detection as described previously ^[24]^.

### DNA extraction, sequencing, and genome assembly

The leaves of *U. rhynchophylla* were used to isolate DNA using a DNA extraction kit (Accurate Biology, China). Agarose (1%) electrophoresis was used to assess the integrity and purity of the DNA. The DNA library was prepared following the standard protocol from Illumina as previously described by Cao et al. (2018). The raw reads were filtered to clean low-quality reads (quality values less than or equal to 5, reads with adapters, and reads with more than 5% N base content) using FastQ (v.0.11.7) (https://www.bioinformatics. babraham.ac.uk/projects/fastqc/), and finally, the clean reads were used for subsequent analysis.

For Oxford Nanopore sequencing, following quality inspection, the genomic DNA was randomly fragmented using Megaruptor (Diagenode, USA), and large DNA fragments were enriched and purified using Agencourt AMPure XP beads (Beckman, USA). Subsequently, BluePippin (Sage Science, USA) was employed to cut and recover the large fragments, and then the DNA was recovered from the beads, repaired and end-modified using the NEBNext Ultra II End-repair/dA tail module. The ligation reaction was carried out using the adapter from the SQK-LSK109 ligation kit (Oxford Nanopore Technologies, USA), and the DNA library was loaded into the Flow cells and transferred to the Oxford Nanopore PromethION (Oxford Nanopore Technologies, USA) for real-time single-molecule sequencing (Ma et al. 2021).

Based on the sequencing reads, the k-mer analysis method was used to estimate the genome size and heterozygosity rates (Song et al. 2018). NECAT software was used for genome error correction and assembly, followed by two rounds of error correction using Racon (version: 1.4.11) software on the assembly results obtained from the Oxford Nanopore sequencing data. Finally, Pilon software (version: 1.23) was used to conduct two rounds of error correction on the basis of Illumina reads to generate the final assembly outcome (Hesse 2023; Ogiso-Tanaka et al. 2022; Ruan and Li 2020).

### Chromosome-level genome assembly using Hi-C

The leaves of *U. rhynchophylla* were used to extract high-quality DNA for Hi-C sequencing. Formaldehyde was used to fix the chromatin. Hi-C chromosome conformation capture was performed as previously reported (Walker et al. 2014). The Hi-C library was sequenced using 350 bp paired-end reads using Illumina HiSeq (Illumina, USA). ALLHIC software (version 0.9.12) was used to cluster the contig sequences into chromosome groups via the agglomerative hierarchical clustering method (Ramani et al. 2016).

### Genome annotation

#### Repeat sequence repeats

RepeatModeler software (version: 1.0.4) was used to construct our own repeat library. After including the repbase library, RepeatMasker (version: 4.0.5) was used to conduct the genome repeat sequence annotation.

### Gene prediction

Gene prediction was conducted using GeneMark (version: 4.57_lic) and Augustus (v.3.0.3) software (Guk et al. 2022). Next, BUSCO software (version: 4.0.6) was used to evaluate the quality of gene prediction.

### Gene functional annotation

The predicted protein sequences were compared with the TE protein library. The protein-coding sequences were compared with seven protein databases, including NR, UniProt, Pfam, GO, KEGG, Swiss-Prot, and InterProScan, using DIAMOND BlastP (version: 0.7.9; parameter: -evalue 1e^−5^) after the TE protein-encoding genes were removed (Hoff et al. 2019).

### Noncoding RNA annotation

In accordance with the structural characteristics of tRNA, tRNAscan-SE (version: 1.23) software was used to identify the tRNA sequences in the *U. rhynchophylla* genome. Next, RNAmmer (version: 1.2) was utilized for rRNA prediction (Liu et al. 2021b), and INFERNAL (version: 1.1.2) was used to identify ncRNA sequences in the *U. rhynchophylla* genome based on the Rfam database (Lagesen et al. 2007).

### Evolutionary analysis

#### Gene family clustering

The protein sequences of 17 plant species, including *Arabidopsis thaliana*, *Catharanthus roseus*, *Coffea arabica*, *Cucumis sativus*, *Eschscholzia californica*, *Gelsemium elegans*, *Gardenia jasminoides*, *Gelsemium sempervirens*, *Glycine max*, *Nicotiana tabacum*, *Mikania cordata*, *Oryza sativa*, *Lonicera japonica*, *Vitis vinifera*, *Siraitia grosvenorii*, *Papaver somniferum*, and *Solanum lycopersicum*, were aligned using BlastP (version: 2.6.0; parameters: -evalue 1e^−5^ -outfmt 6) (Nawrocki and Eddy, 2013), and finally, OrthoMCL software (v.2.0.9; parameters: percentMatchCutoff = 30, evalueExponentCutoff = 1e^−5^, expansion coefficient 1.5) was used for gene family clustering (Jacob et al. 2008).

#### Construction of the phylogenetic tree

A phylogenetic tree was constructed based on 142 single-copy gene families shared among the 17 species. Initially, multiple sequence alignment of the protein sequences in each single-copy gene family was performed using MUSCLE (version: 3.8.31), followed by alignment filtering with TrimAl (version: 1.4) (-gt 0.2), and finally, a maximum likelihood tree was constructed using RAxML (version: 8.2.10) (Capella-Gutiérrez et al. 2009; Edgar 2010; Li et al. 2003).

#### Gene family contraction and expansion

On the basis of gene family clustering, CAFE software (version 2.1; parameter:–filter) was used to analyse gene family contraction and expansion.

#### Whole-gene replication analysis

Four species (*U. rhynchophylla*, *G. jasminoides*, *C. arabica*, and *V. vinifera*) were selected for whole-gene replication analysis. First, the protein sequences of different species were subjected to BLAST analysis (version: 2.6.0 +; parameters: -value 1e^*−*5^ and -outfmt 6). The sequences were subsequently paired and analysed using MCScanX (https://github.com/wyp1125/MCScanx; parameters:-a, -e 1e^−5^, and -s^5^) to identify genome colinear blocks. The synonymous mutation frequency (Ks), nonsynonymous mutation rate (Ka), and ratio of nonsynonymous mutations to synonymous mutation rates (Ka/Ks) of the collinear gene pairs were calculated using PAML (version: 4.9) (Stamatakis 2014). Finally, the results were visualized using the ggplot2 package (version: 2.2.1) in Rv4.1.2 (www.r-project.org).

### Divergence time analysis

Based on the evolutionary tree results of 18 species, the estimation of divergence time among different species was conducted using the mcmctree module of PAML (version: 4.9) with the following parameters: nsample = 1 000 000; burn = 200 000; seqtype = 0; model = 4. The reference fossil node is *V. vinifera*, *O. sativa*: 125–150 million years ago; *A. thaliana*, *G. max*: 98–117 million years ago.

### Transcriptome sequencing of* U. rhynchophylla*

Total RNA was extracted from three different tissues (roots, leaves, and stem hooks) of *U. rhynchophylla* using the FastPure Cell/Tissue Total RNA Isolation Kit (Vazyme, China). Agarose gels (1%) were used to assess RNA integrity, and the concentration of total RNA was measured using Multiskan SkyHigh (Thermo Fisher, USA). The experimental process was carried out according to the standard protocol provided by Oxford Nanopore Technologies (ONT). Moreover, Illumina RNA-seq (Illumina NovaSeq, USA) was used to obtain RNA-seq reads. STAR software (v.2.7.0; parameters: –twopassMode None) was used to remap the RNA-seq data to the reference genome (Yang 2007). Additionally, transcript per kilobase per million mapped reads (TPM) values were used to assess gene expression in each sample using RSEM software (version: 1.2.15) (Dobin et al. 2013).

### Investigation of key enzyme-encoding genes in the MIA biosynthetic pathway

As shown in Fig. 1, KEGG pathway map00901 was employed to screen all candidate genes involved in the RIN and IRN biosynthetic pathways. The gene expression patterns in different tissues were visualized using the TBtools (v.2.096) heatmap module.

### cDNA synthesis and qRT‒PCR analysis

Total RNA (500 ng) was reverse transcribed with HiScript III All-in-one RT SuperMix Perfect for qPCR (Vazyme, China). The reaction was conducted in 96-well plates with qTOWER^3^ G (Analytik, Germany). The 20 μL reaction system included 10 μL of 2 × SYBR qPCR Master Mix (Vazyme, China), 2 μL of cDNA, 0.8 μL of each primer (forward and reverse), and 7.2 μL of DEPC-treated water. The amplification reaction procedure was as follows: 95 °C, 3 min; 95 °C, 15 s; 60 °C, 30 s; 40 cycles; 95 °C, 15 s; 60 °C, 30 s; and 95 °C, 15 s. Each qRT‒PCR analysis was performed with three technical replicates. The relative gene expression was calculated using the 2^−∆∆CT^ method (Shao et al. 2024a).

### Heterologous expression of recombinant UrTDC and UrLAMT and enzyme activity assay

The full-length coding sequences (CDS) of *UrLAMT2* (1137 bp) and *UrTDC* (1530 bp) were cloned by PCR amplification using *U. rhynchophylla* cDNA under the following cycling conditions: 95 °C for 3 min; 35 cycles of 95 °C for 30 s, 55 °C for 30 s and 72 °C for 1.5 min; 72 °C for 5 min. The resulting PCR amplification products were purified using the GeneJET Gel Extraction Kit (Thermo Fisher, USA)and then inserted into the pET28a-His vector, which was subsequently digested with Nde I and EcoR I using the *OK Clon* DNA Ligation Kit (Accurate Biology, China), after which *UrLAMT1* (1116 bp) was cloned and inserted into the pCold-TF vector. The *E. coli* strain DH5α was used to construct the recombinant plasmids, and *E. coli* BL21 (DE3) was used as a medium for heterologous expression of the recombinant UrTDC and UrLAMT proteins. Single colonies containing the recombinant plasmid were inoculated in LB liquid medium, followed by incubation of the sample at 37 °C and 200 rpm for 12 h. The culture was subsequently transferred into 400 mL of liquid LB medium supplemented with kanamycin until the OD600 reached 0.6–0.8. Next, IPTG at a final concentration of 0.1 mM was added for protein expression. The cells were collected by centrifugation at 5000 rpm for 10 min following a 20 h incubation period at 16 °C and 120 rpm. Next, the cells were resuspended in lysis buffer (45 mM Tris–HCl, 300 mM NaCl, 20 mM imidazole, and 10% glycerol, pH 7.5) and lysed by sonication on ice for 20 min.

The supernatant was collected by centrifugation at 10,000 rpm and 4 °C for 30 min. Protein purification was carried out using affinity chromatography, with the supernatant being loaded onto an equilibrated gravity column that contained Ni Focurose FF (IMAC) (Huiyan Biology, China). After the supernatant was removed, four column volumes of lysis buffer were used to wash the nickel column. The recombinant protein was then eluted with elution solution (20 mM PB, 20 mM imidazole, 500 mM NaCl, 10% glycerol, and 5 mM β-mercaptoethanol) at 25 °C. The proteins were further concentrated in an ultrafiltration tube at 4000 rpm and 4 °C. The purified proteins were detected by sodium dodecyl sulfate‒polyacrylamide gel electrophoresis (SDS‒PAGE), and the protein concentration was measured using a Micro Drop (BIO-DL, China).

In the presence of pyridoxal-5'-phosphate (PLP), UrTDC was able to catalyse the decarboxylation of L-tryptophan to form tryptamine, and in the presence of S‐adenosyl methionine (SAM), LAMT catalysed the conversion of loganate into loganin. To measure the enzyme activity of recombinant UrTDC or UrLAMT, an assay was conducted using a mixture of 100 µL of Tris–HCl buffer. The mixture contained 10 µg UrTDC or UrLAMT, 200 µm L-tryptophan or loganate, and 250 µm PLP or SAM and had a pH of 7.5. Equal masses of high-temperature inactivated protein were used as controls. The reaction mixtures were incubated at 37 °C for 1 h and then terminated by adding an equal volume of methanol.

To optimize the conditions of the UrLAMT and UrTDC protein catalytic reactions, enzymatic reactions were carried out at various temperatures (15 °C, 20 °C, 25 °C, 30 °C, 35 °C, 40 °C, 45 °C, 50 °C, 55 °C, 60 °C and 65 °C) to determine the impact of temperature on the activity of the catalytic reaction of the recombinant protein UrTDC or UrLAMT. The optimal pH conditions for enzymatic reactions were determined by assessing the activity of UrTDC or UrLAMT during incubation at various pH values (pH 5–11). Three types of buffer systems were utilized, namely, 50 mM PBS buffer (with a pH range of 5.5 to 7.0), 50 mM Tris–HCl (with a pH range of 7.5 to 9.0), and 50 mM glycine–NaOH (with a pH range of 9.5 to 11.0). All the reaction products were assayed following a 1 h incubation period. To examine the impact of various metal ions, such as Cu^2+^, NH_4_^+^, Li^+^, Ca^2+^, Mg^2+^, Fe^2+^, Fe^3+^, and Al^3+^, on the functionality of the UrTDC or UrLAMT proteins, the isolated proteins were first exposed to each metal ion (0.1 mM) in PBS buffer at pH 7.0 for 30 min on ice. Following the sequential addition of L-tryptophan and PLP or loganate and SAM to the reaction system, the mixtures were incubated under the optimal reaction conditions determined in the aforementioned studies. HPLC detection was performed as previously described (Li and Dewey 2011; You et al. 2021).

### Identification of candidate *UrWRKY* genes in *U. rhynchophylla*

HMMER 3.2 was used to identify the candidate *UrWRKY* genes with Pfam domain PF03106 (Hu et al. 2021; You et al. 2021). The CD-search (https://www.ncbi.nlm.nih.gov/Structure/cdd/wrps b.cgi) and SMART (https://smart.embl.de/) websites confirmed that all UrWRKY proteins had complete conserved WRKY domains. Then, ExPASy (https://web.expasy.org/protparam/) was used to calculate the amino acid length, molecular weight (MW), theoretical isoelectric point (pI) and instability index of all UrWRKY proteins, and WoLF PSORT (https://wolfpsort.hgc.jp/) was used to predict their subcellular localization (Guo et al. 2019).

### Multiple sequence alignment and phylogenetic tree construction

The WRKY protein sequences from *A. thaliana* were downloaded from the TAIR database (https://www.arabidopsis.org/index.jsp) as the query sequences to identify homologous UrWRKY sequences. MEGA7.0 software (ClusterW) was used to construct the phylogenetic tree between *U. rhynchophylla* and *A. thaliana* using the neighbour-joining method and 1000 bootstrap values (Yang et al. 2021). The UrWRKY protein sequences containing conserved domains were aligned using DNAMAN 7.0 software (Ye et al. 2021).

### Chromosomal locations, gene duplications and collinearity analysis

The locations of the *UrWRKY* genes on the 22 chromosomes of *U. rhynchophylla* were identified using TBtools, and the results were visualized using Circos (http://circos.ca/). BlastP and MCScanX were used to analyse the gene duplication events of the *UrWRKY* genes using the default parameters (Cheng et al. 2022). Collinearity analysis between *U. rhynchophylla* and *A. thaliana*, *O. sativa*, and *C. canephora* was performed using TBtools software.

### Conserved motifs, gene structures, and *cis*-acting elements

The MEME v5.1.1 online tool (https://meme-suite.org/meme/) was employed to predict conserved motifs for each UrWRKY TF using the following specific parameters: a maximum of 10 motifs and motif widths ranging from 20 to 50 (Wu et al*.*, 2023). The gene sequences of these genes were utilized for gene structure analysis by TBtools. The illustrations displaying the conserved motifs and gene structures were revised using Illustrator 2021 (v24.0.0.330).

TBtools software (GTF/GFF3 sequence extractor) was used to retrieve the 2.0 kb sequences preceding the translation initiation codons in *U. rhynchophylla*. The binding sites for UrWRKYs were identified through analysis of the 2000 bp promoter sequences of associated enzyme-encoding genes in the MIA biosynthetic pathway. The PlantCARE tool (http://bioinformatics.psb.ugent.be/webtools/plantcare/html/) was used to predict *cis*-acting elements within the promoter regions (Yue et al. 2019).

### Co-expression network between UrWRKYs and MIA biosynthetic genes

To determine which UrWRKYs regulate the MIA biosynthetic pathway, the gene expression profiles of the *UrWRKY* genes and key enzyme-encoding genes in the MIA biosynthetic pathway were used to establish a co-expression network using the Partial Correlation Coefficients method in IBM SPSS statistics 22 (Lescot et al. 2002). Finally, the results were visualized using Cytoscape version 3.7.0 (Montenegro 2022). The correlation between *UrWRKY37* and UR-MIAs was visualized using TBtools.

### Subcellular localization assay

To investigate the subcellular localization of UrWRKY37, the full-length *UrWRKY37* was amplified and integrated into the pBI121-GFP vector, with the restriction enzyme sites *Nde* I and *Sam* I used to generate the pBI121-UrWRKY37-GFP recombinant plasmid. H2B-RFP was used as a specific nuclear fluorescent marker. pBI121-UrWRKY37-GFP, pBI121-GFP, and H2B-RFP were partly transformed into *Agrobacterium tumefaciens* GV3101, which was subsequently injected into 21-day-old tobacco leaves after 48 h of cultivation. Fluorescence signals were observed using a fluorescence microscope (Zeiss Apotome 2, Germany).

### Yeast one-hybrid (Y1H) and dual-luciferase (dual-LUC) assays

The complete coding sequence (CDS) of *UrWRKY37* (1689 bp) was cloned and inserted into the pGADT7 vector as the prey vector AD-UrWRKY37. The promoter sequences of *UrTDC* and *UrSGD* were cloned and inserted into the pAbAi vector as bait vectors BD-*UrTDC* and BD-*UrSGD*, respectively. BD-*UrTDC* and BD-*UrSGD* were individually transformed into the yeast strain Y1H Gold, and the self-activation concentration was screened on SD medium lacking Ura (SD/-Ura). AD-UrWRKY37 was transformed into Y1H Gold containing BD-*UrTDC* or BD-*UrSGD*, and the interaction analysis was performed on SD medium lacking Leu (SD/-Leu) with or without aureobasidin A (AbA). The pGADT7 empty vector was transformed into Y1H bait strains as a negative control, and pGADT7-p53 was transformed into Y1H (pAbAi-p53) as a positive control.

The full-length coding sequence of *UrWRKY37* was amplified and inserted into the pGreenII-62SK vector as an effector via digestion with *Bam*H I and *Eco*R I. The 2000 bp promoter regions of *UrTDC* and *UrSGD* were then inserted into the pGreen0800-LUC vector as reporter via digestion with *Kpn* I and *Bam*H I and separately transformed into *A. tumefaciens* GV3101-p19 strains. The empty pGreenII-62SK vector was used as the control. The experimental methods for detection have been previously reported (Wang et al*.*, 2022). The luciferase/Renilla luciferase (LUC/REN) activity ratio represents the binding activity. All the experiments were repeated four times.

### Acquisition of UrWRKY37 transgenic hairy roots

Leaves were isolated from *U. rhynchophylla* tissue plant seedlings and cultivated on WPM solid medium supplemented with 2.0 mg/L 2,4-D and 0.5 mg/L 6-BA in the dark for 2 days. The *A. tumefaciens* strain C58C1, including the pBI121-UrWRKY37-GFP plasmid and the empty pBI121-GFP vector, was used to infect the leaves, which were then cultured on WPM solid medium in the dark for 3 days and transferred to 1/2 MS solid medium supplemented with 50 μL/L kanamycin. Positive transgenic lines were identified via PCR using the corresponding primers (rolB-F:GCTCTTGCAGTGCTAGATTT, rolB-R:GAAGGTGCAAG CTACCTCTC). Positive OE-*UrWRKY37* transgenic hairy roots were cultured in 1/2 MS liquid medium under dark conditions at 25 °C and 110 rpm for 30 days.

## Conclusion

In this study, ten target MAs were detected in three different tissues of *U. rhynchophylla*, with most MIAs found to accumulate in stem hooks rather than in leaves and roots. A high-quality chromosomal-level *U. rhynchophylla* genome was constructed. The genome size of *U. rhynchophylla* was 627.70 Mb, with a contig N50 of 1.83 Mb, and 46,909 genes were annotated. According to the current knowledge of the MIA biosynthetic pathways, a total of 64 candidate key enzyme-encoding genes were selected, and their expression patterns were assessed in three different tissues via transcriptome analysis. We verified that UrTDC6 functions to convert L-tryptophan to tryptamine and that UrLAMT1/2 can catalyse the conversion of loganate to loganin. Furthermore, 72 *UrWRKY* genes were identified in *U. rhynchophylla*. Dual-LUC and Y1H assays revealed that UrWRKY37 activated the expression of *proUrTDC* and *proUrSGD* and that overexpression of *UrWRKY37* increased the alkaloid content of *U. rhynchophylla* in transgenic hairy roots. Our study lays the foundation for analysing the MIA biosynthetic pathways and clarifying the relevant molecular regulatory mechanisms in *U. rhynchophylla*.

## Supplementary Information

Supplementary Material 1. Supplemental material 1: Fig S1. The estimation of genome size and heterozygosity of *U. rhynchophylla*using K-mer. The figure shows frequency of 19-mer depth distribution of the genome sequencing reads. Fig S2. Distribution of genes and gene families across 17 plant species. The red star represents the *U. rhynchophylla* in this study. Fig S3. The Go enrichment of genes specific to *U. rhynchophylla*. The green square represents the indole alkaloid metabolic process. Fig S4. Phylogenetic analysis and divergence time estimations among 18 plant species. The tree was constructed based on all single-copy orthologous genes using PAML software. Divergence times estimated in million years ago are indicated by the blue numbers over the nodes. Fig S5. Phylogenetic tree for *U. rhynchophylla* and 17 other plants. Expansion and contraction of gene families are denoted as numbers with plus and minus signs, respectively. Fig S6. The synonymous substitution rate (Ks) distribution plot for paralogs and orthologs of *U. rhynchophylla *with *C. arabica*, *G. jasminoides*, and *V.vinifera* as shown through colored continuous and dotted lines, respectively. Fig S7. The relative expression of *UrTDC6* in root, stem hook, and leaf. * represents*p* < 0.05; ** represents *p* < 0.01; **** represents *p*< 0.0001. Fig S8. Multiple sequences alignment of the TDC proteins from *C. arabica*, *C. eugenioides*, *O. pumila*, *N. tomentosiformis*,*L. ferocissimum*, *L. barbarum*, *C. acuminata*, and *U. rhynchophylla* using DNAMAN7.0 software. The highly conserved domain in each group is in dark blue. The red square represented the conserved domain. Fig S9. The phylogenetic tree was constructed using TDC proteins from *C. arabica*,*C. eugenioides*, *M. speciosa*, *O. pumila*, *N. tomentosiformis*, *L. ferocissimum*, *L. barbarum*, *C. acuminata*, and *U. rhynchophylla* using MEGA7.0 software. Fig S10. The SDS-PAGE of uninduced, crude, and purified UrTDC6. The square represented the target protein. Fig S11. The relative expression of *UrLAMT1* and *UrLAMT2* in three different tissues. ** represents *p* < 0.01; **** represents *p*< 0.0001. Fig S12. Multiple sequences alignment of the LAMT proteins from *C. roseus*, *O. pumila*, *U. tomentosa*, *M. speciosa*, *H. patens*, *L. japonica*, *C. florida*, *E. herrerae*, *U. rhynchophylla* using DNAMAN7.0 software. The highly conserved domain in each group is in dark blue. The red square represented the Loganate bingding site. Fig S13. The SDS-PAGE of uninduced, crude, and purified UrLAMT1 and UrLAMT2. The square represented the target protein. Fig S14. Multiple sequences alignment of the 72 UrWRKY proteins using DNAMAN7.0 software. The highly conserved domain in each group is in dark blue. The red lines represent the WRKY domains, and the blue lines indicated the C_2_H_2_ or C_2_HC zinc finger motifs. Fig S15. Conserved motifs, gene structure, and *cis*-acting elements analysis of *UrWRKY* genes. **A** Sequence logos for motif 1-8. **B** The phylogenetic tree of UrWRKY protein was constructed using MEGA7.0. **C** Motif compositions of UrWRKY proteins. Eight motifs are represented by different colored boxes with numbered 1-8. **D** Exon-intron structures analysis of *UrWRKY* genes. **E** *Cis*-acting elements analysis in the *UrWRKY* promoter region were carried out. The number of *cis*-acting elements are presented in numerical form. The greater the quantity, the darker the color. Blue, yellow, green represent three categories of *cis*-acting elements related to plant growth and development, phytohormone responsive, and abiotic and biotic stress. Fig S16. Collinearity analysis of the *UrWRKY* gene family in the *U. rhynchophylla* genome assembly. **A** Chromosomal distribution and syntenic relationships of *UrWRKY* genes. Chromosomal locations and syntenic relationships are depicted in a circular diagram. Gray lines represent all syntenic blocks in the *U. rhynchophylla* genome, while red lines indicate segmentally duplicated *UrWRKY* gene pairs. Tandemly duplicated genes are marked with red boxes. Chromosomes 1-22 are color-coded for clarity. **B**-**D** Comparative synteny of* WRKY* genes between *U. rhynchophylla* and three representative species: *A. thaliana*, *O. sativa*, and *C. camephora*. Collinear *UrWRKY* gene pairs are highlighted in red within gray syntenic blocks. Fig S17. **A** The phenotype of the *pBI121-EV* transgenic hairy roots. **B** The phenotype of the *UrWRKY37-OE* transgenic hairy roots.

Supplementary Material 2. Supplemental material 2: Table S1. Statistics of Illumina Novaseq sequencing data. Table S2. K-mer analysis data statistics. Table S3. Statistical of the assembly results of the *U. rhynchophylla* genome. Table S4. The 22 chromosomes assembled of *U. rhynchophylla* genome based on Hi-C sequencing. Table S5. The summary of BUSCO evaluation of genome assembly. Table S6. The statistics of coding gene prediction results. Table S7. The statistics of coding gene prediction results. Table S8. The statistics of repeated sequence annotation. Table S9. Comparisons of genes and gene families among plant species we investigated. Table S10. The statistics of RIN and IRN biosynthesis genes. Table S11. The statistics of *CYP450* genes in *U. rhynchophylla* genome. Table S12. The detailed information of WRKY transcription factor family in *U. rhynchophylla*. Table S13. The syntenic analysis of *WRKY* genes between *U. rhynchophylla* and *A.thaliana*,*O.sativa*, and *C. camephora*.

## Data Availability

Not applicable.

## Abbreviations

AbA: Aureobasidin A
bHLH: Basic/helix-loop-helix
bZIP: Basic leucine zipper
Dual-luc: Dual-luciferase assay
EMSA: Electrophoretic mobility shift assay
GFP: Green fuorescent protein
LUC: Firefy luciferase
LC-MS: Liquid Chromatograph Mass Spectrometer
MIAs: Monoterpenoid indole alkaloids
ORF: Open reading frame
qRT-PCR: Quantitative real-time polymerase chain reaction
REN: Renilla luciferase
TRV: Tobacco rattle virus
TF: Transcription factor
VIGS: Virus induced gene silencing
Y1H: Yeast one-hybrid
