# Supplementary material for: The genome of the medicinal plant Uncaria rhynchophylla provides new insights into monoterpenoid indole alkaloid metabolism and its molecular regulatory mechanism
Source: Mol Hortic. 2026 Feb 3;6:9. doi: 10.1186/s43897-025-00187-7 (PMC12866418; doi:10.1186/s43897-025-00187-7)
Supplement: Supplementary file 1 — Supplementary Material 1. Supplemental material 1: Fig S1. The estimation of genome size and heterozygosity of U. rhynchophyllausing K-mer. The figure shows frequency of 19-mer depth distribution of the genome sequencing reads. Fig S2. Distribution of genes and gene families across 17 plant species. The red star represents the U. rhynchophylla in this study. Fig S3. The Go enrichment of genes specific to U. rhynchophylla. The green square represents the indole alkaloid metabolic process. Fig S4. Phylogenetic analysis and divergence time estimations among 18 plant species. The tree was constructed based on all single-copy orthologous genes using PAML software. Divergence times estimated in million years ago are indicated by the blue numbers over the nodes. Fig S5. Phylogenetic tree for U. rhynchophylla and 17 other plants. Expansion and contraction of gene families are denoted as numbers with plus and minus signs, respectively. Fig S6. The synonymous substitution rate (Ks) distribution plot for paralogs and orthologs of U. rhynchophylla with C. arabica, G. jasminoides, and V.vinifera as shown through colored continuous and dotted lines, respectively. Fig S7. The relative expression of UrTDC6 in root, stem hook, and leaf. * representsp < 0.05; ** represents p < 0.01; **** represents p< 0.0001. Fig S8. Multiple sequences alignment of the TDC proteins from C. arabica, C. eugenioides, O. pumila, N. tomentosiformis,L. ferocissimum, L. barbarum, C. acuminata, and U. rhynchophylla using DNAMAN7.0 software. The highly conserved domain in each group is in dark blue. The red square represented the conserved domain. Fig S9. The phylogenetic tree was constructed using TDC proteins from C. arabica,C. eugenioides, M. speciosa, O. pumila, N. tomentosiformis, L. ferocissimum, L. barbarum, C. acuminata, and U. rhynchophylla using MEGA7.0 software. Fig S10. The SDS-PAGE of uninduced, crude, and purified UrTDC6. The square represented the target protein. Fig S11. The relative expressio [file 43897_2025_187_MOESM1_ESM.docx]

**Supplementary material 1**

**
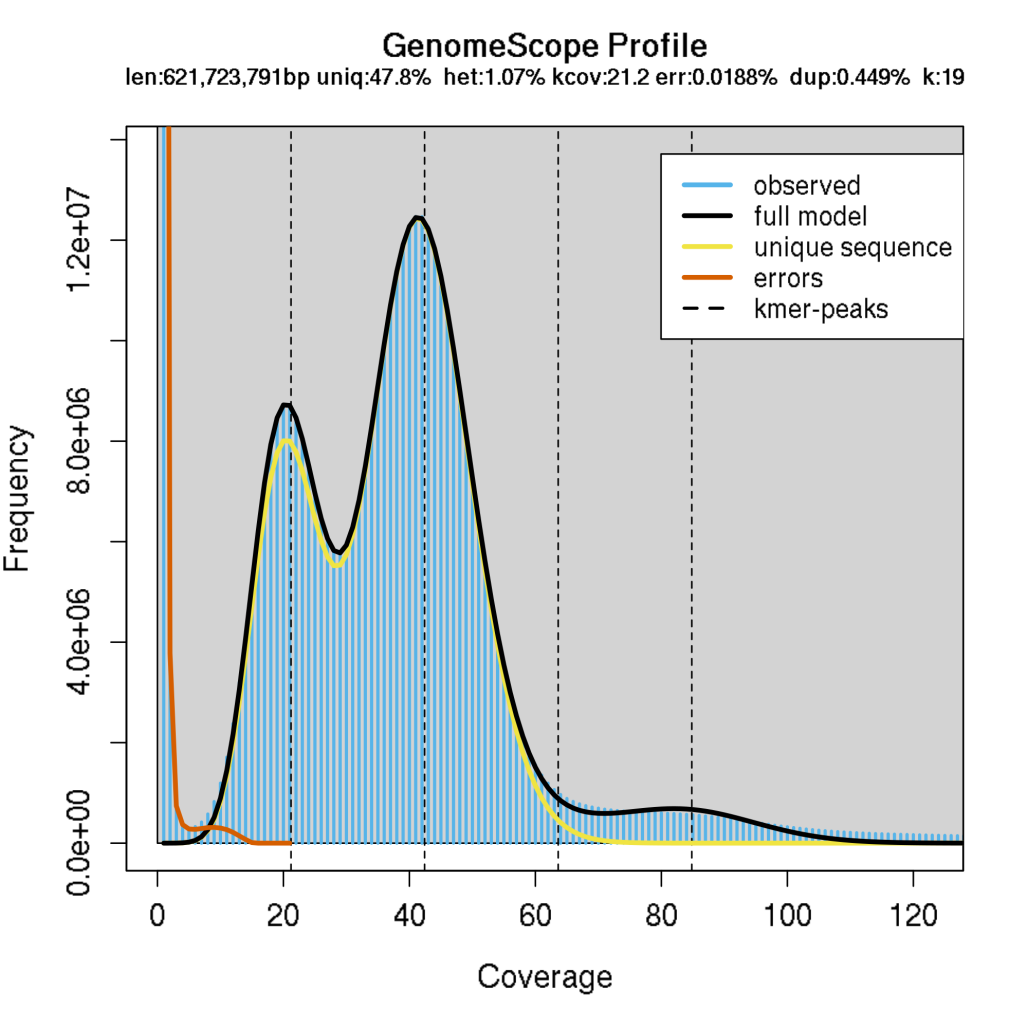
**

**Fig. S1.** The estimation of genome size and heterozygosity of *U. rhynchophylla* using K-mer. The figure shows frequency of 19-mer depth distribution of the genome sequencing reads.

**
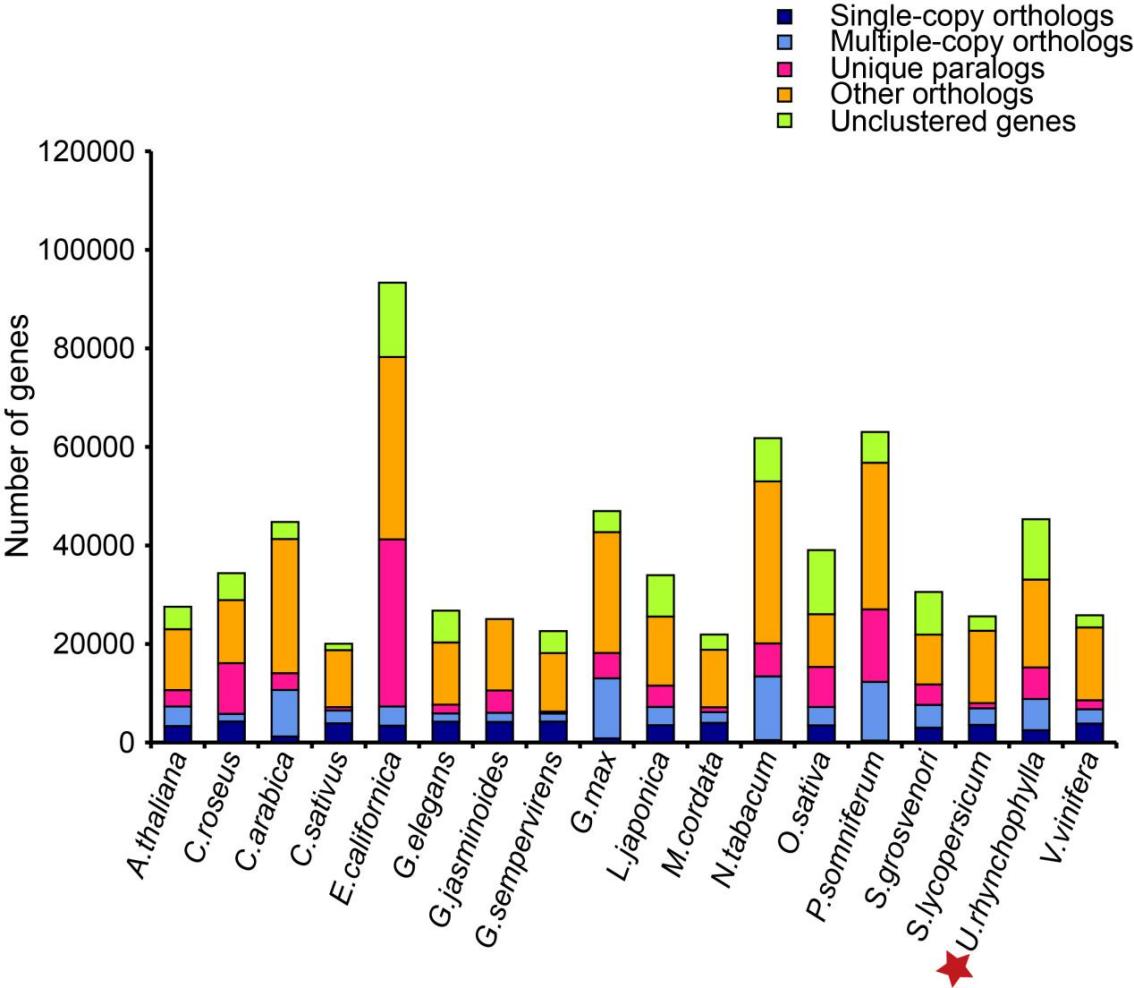
**

**Fig. S2.** Distribution of genes and gene families across 17 plant species. The red star represents the *U. rhynchophylla* in this study.

**
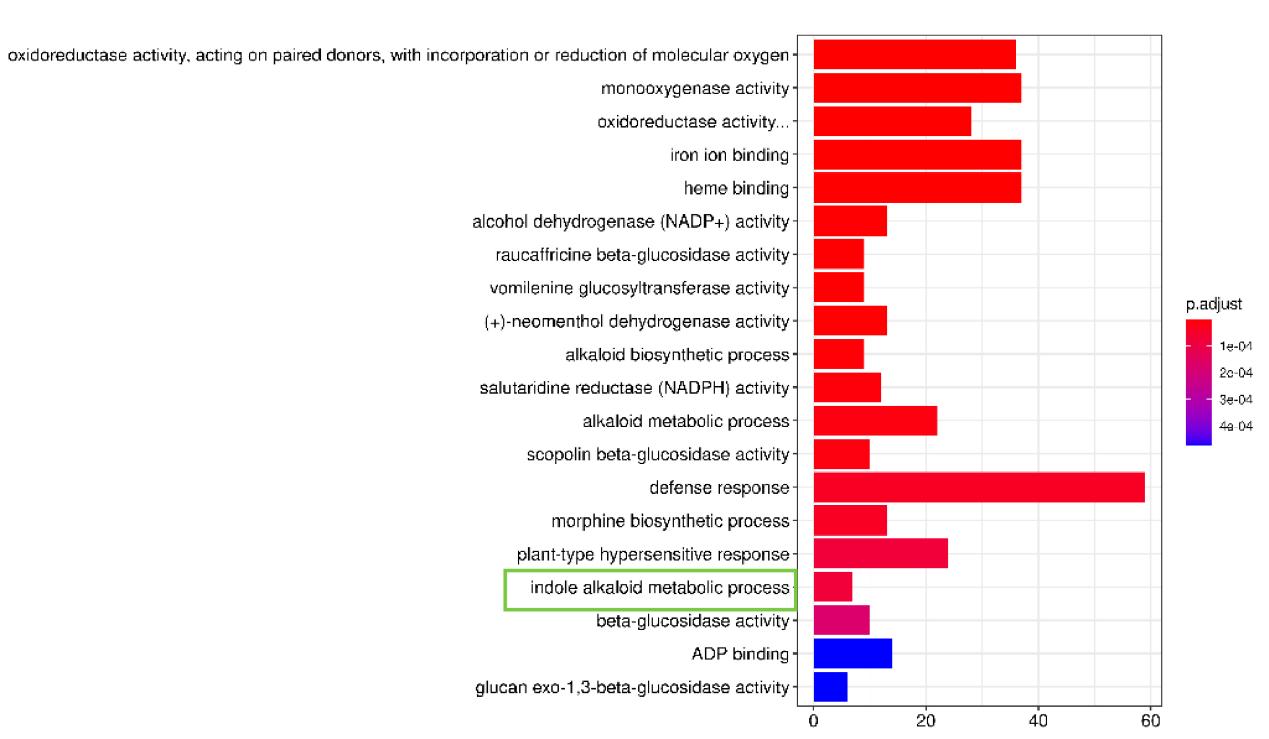
**

**Fig. S3.** The Go enrichment of genes specific to *U. rhynchophylla*. The green square represents the indole alkaloid metabolic process.

**
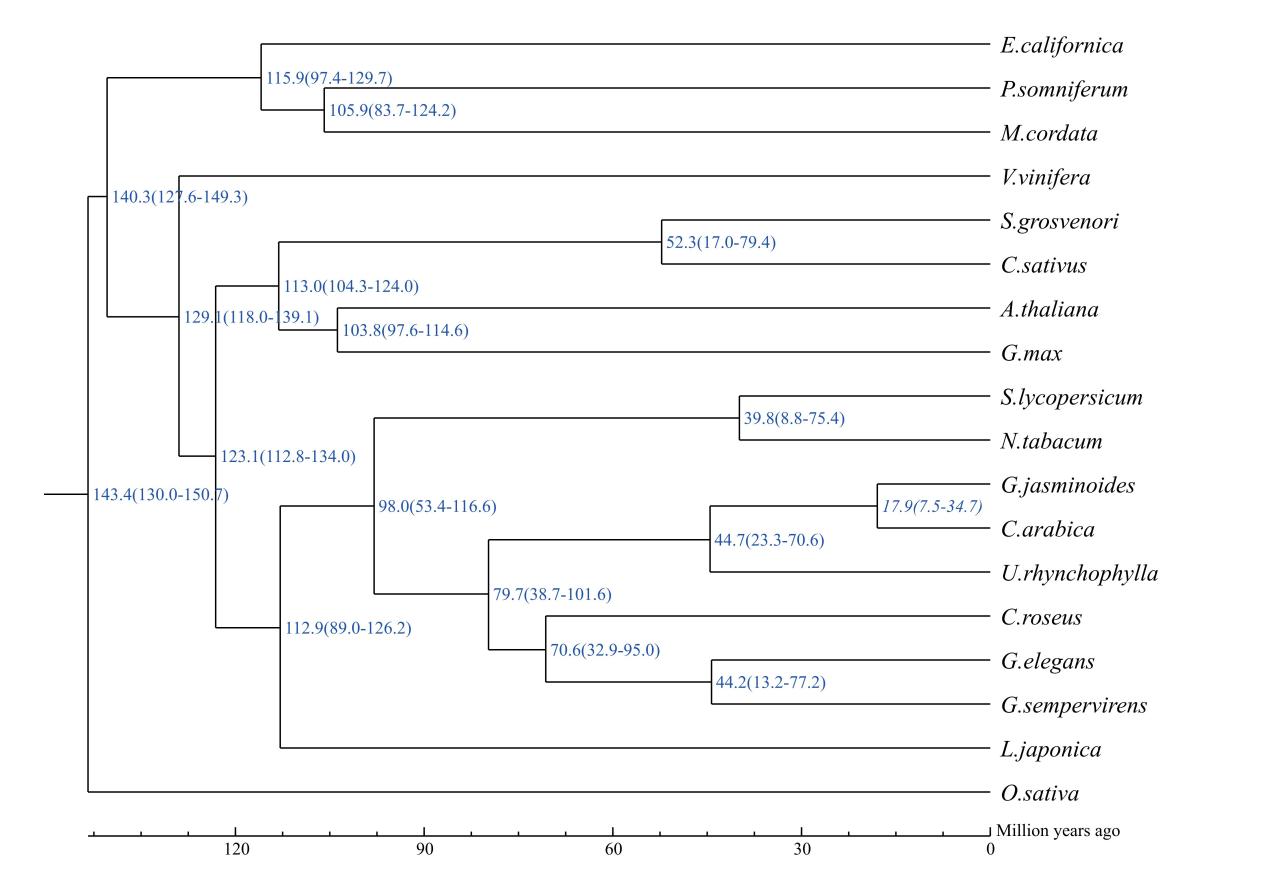
**

**Fig. S4.** Phylogenetic analysis and divergence time estimations among 18 plant species. The tree was constructed based on all single-copy orthologous genes using PAML software. Divergence times estimated in million years ago are indicated by the blue numbers over the nodes.


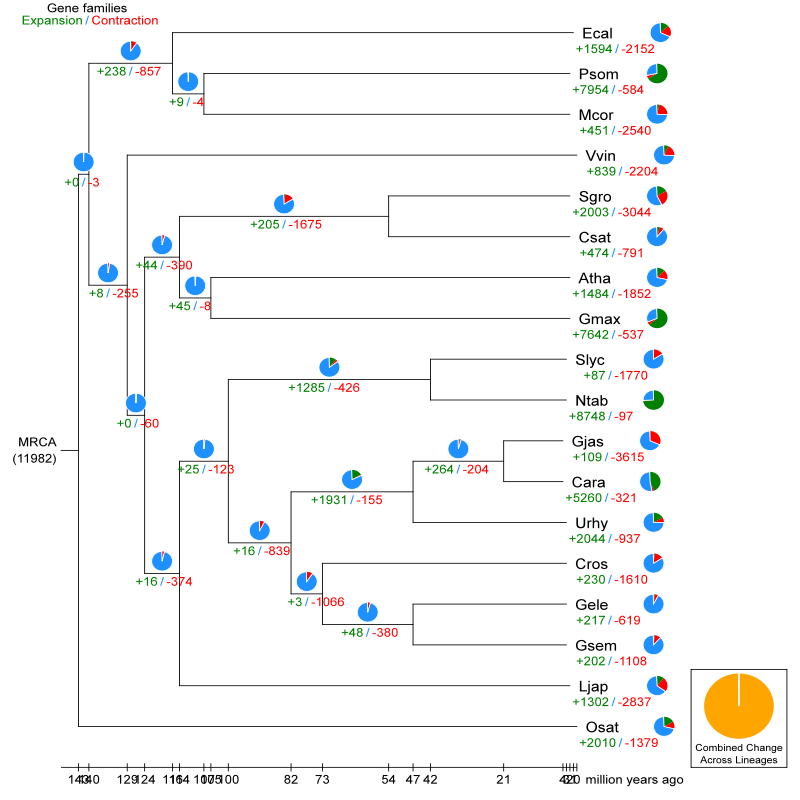


**Fig. S5.** Phylogenetic tree for *U. rhynchophylla* and 17 other plants. Expansion and contraction of gene families are denoted as numbers with plus and minus signs, respectively.


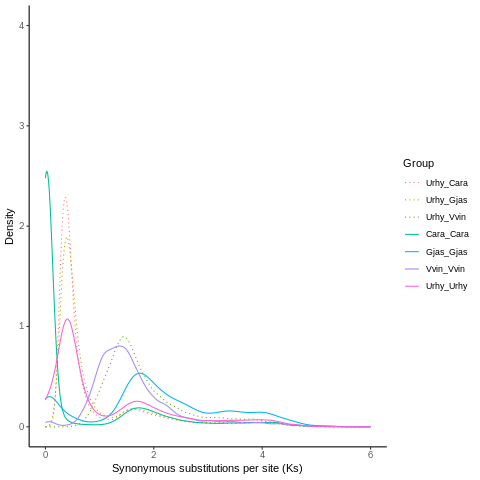


**Fig. S6.** The synonymous substitution rate (Ks) distribution plot for paralogs and orthologs of *U. rhynchophylla* with *C. arabica*, *G. jasminoides, and V.vinifera* as shown through colored continuous and dotted lines, respectively.


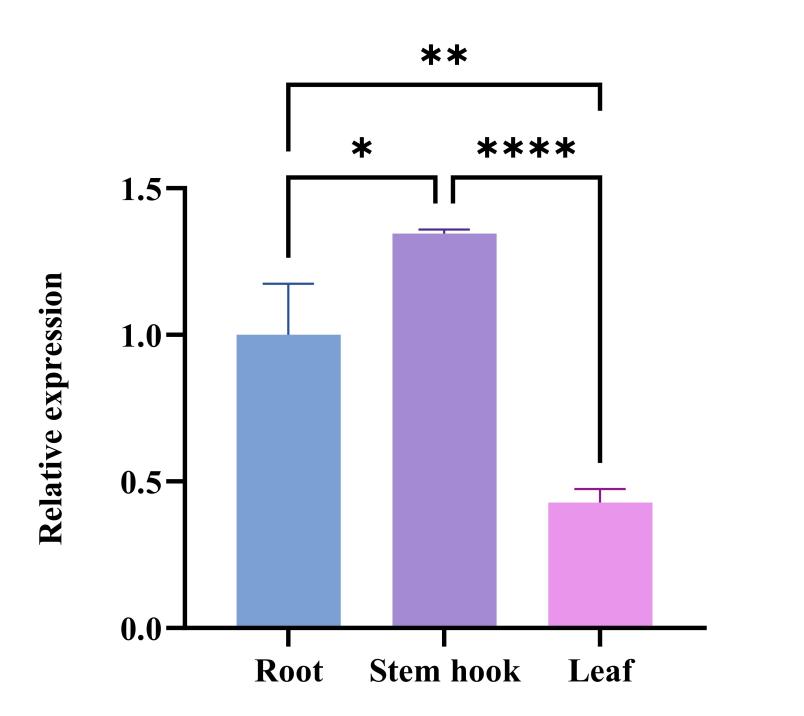


**Fig. S7.** The relative expression of *UrTDC6* in root, stem hook, and leaf. * represents *p* < 0.05; ** represents *p* < 0.01; **** represents *p* < 0.0001.


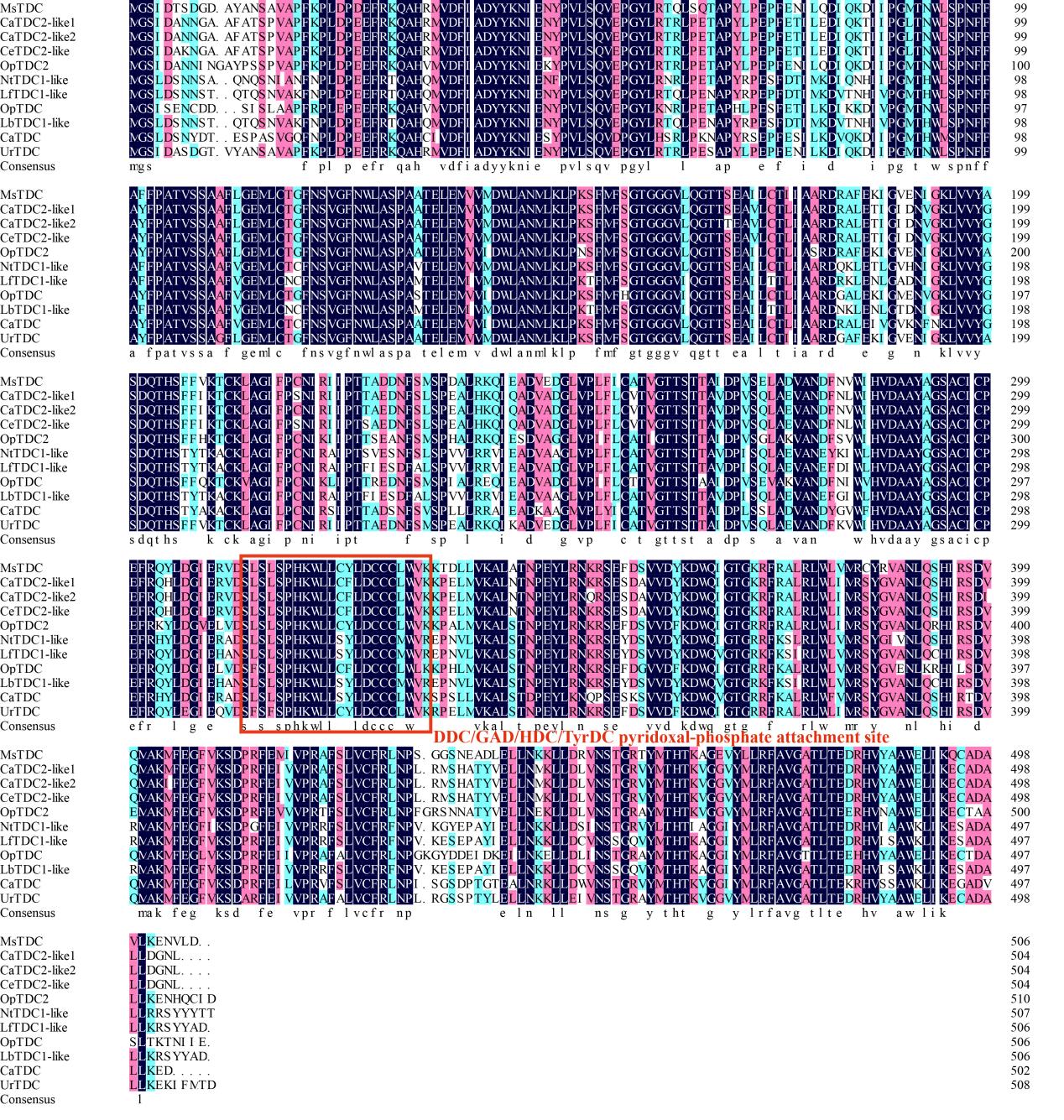


**Fig. S8.** Multiple sequences alignment of the TDC proteins from *C. arabica*, *C. eugenioides*, *O. pumila*, *N. tomentosiformis*, *L. ferocissimum*, *L. barbarum*, *C. acuminata*, and *U. rhynchophylla* using DNAMAN7.0 software. The highly conserved domain in each group is in dark blue. The red square represented the conserved domain.
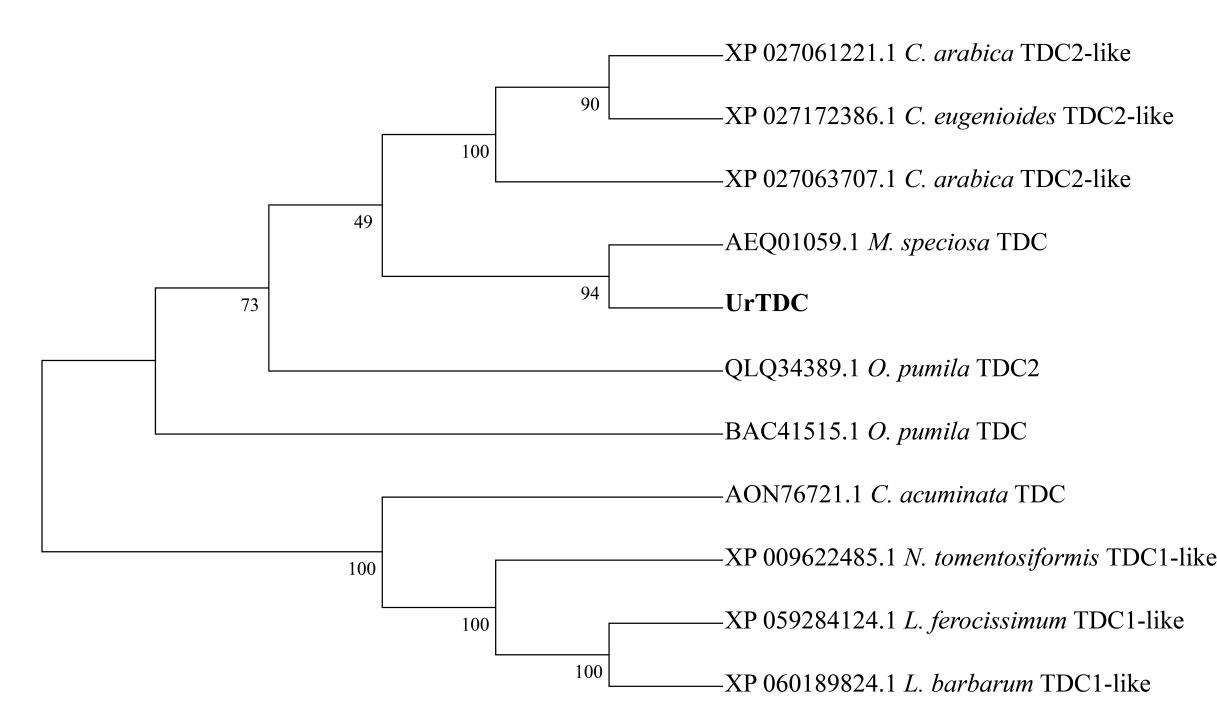


**Fig. S9.** The phylogenetic tree was constructed using TDC proteins from *C. arabica*, *C. Eugenioides*, *M. speciosa*, *O. pumila*, *N. tomentosiformis*, *L. ferocissimum*, *L.barbarum*, *C. acuminata,* and *U. rhynchophylla* using MEGA7.0 software.


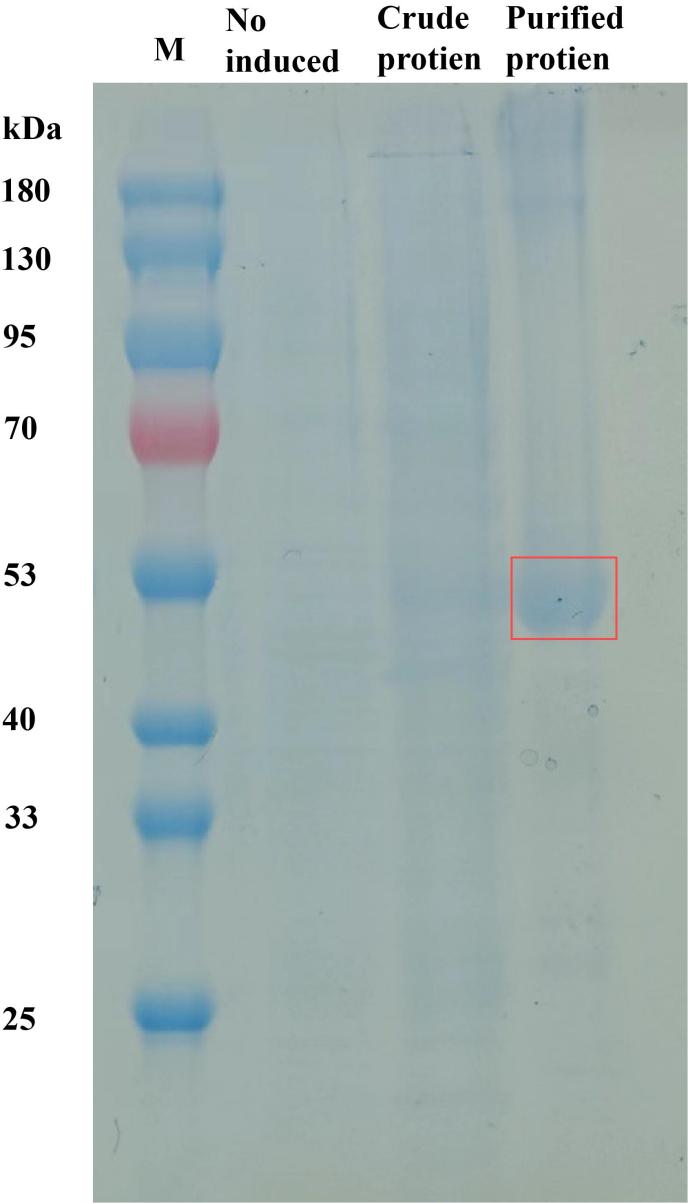


**Fig. S10.** The SDS-PAGE of uninduced, crude, and purified UrTDC6. The square represented the target protein.


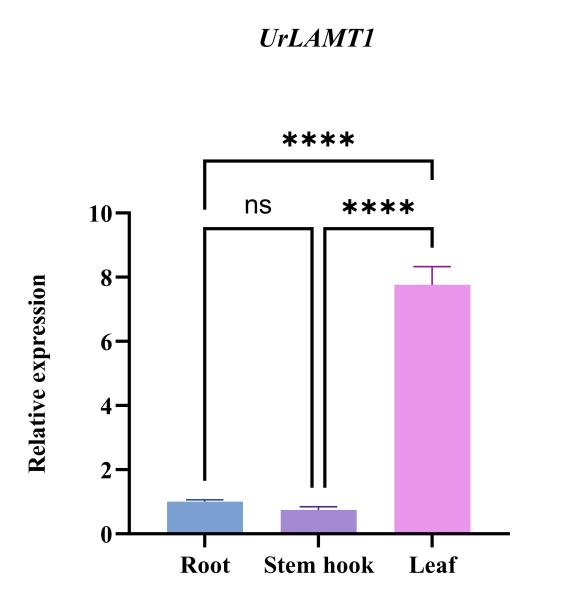
**
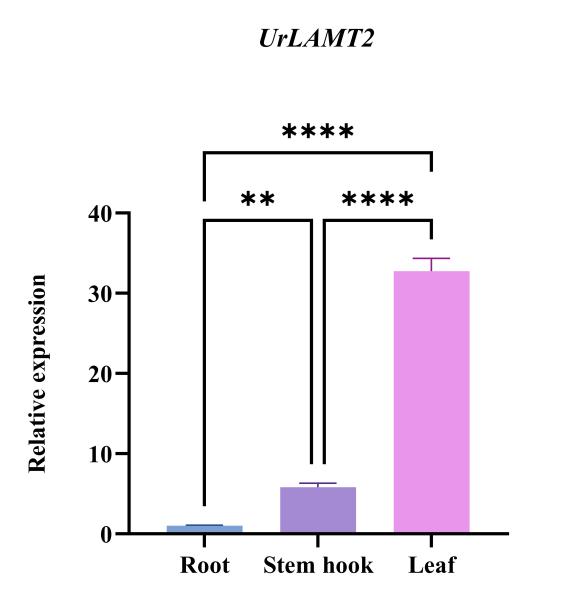
**

**Fig. S11.** The relative expression of *UrLAMT1* and *UrLAMT2* in three different tissues. ** represents *p* < 0.01; **** represents *p* < 0.0001.


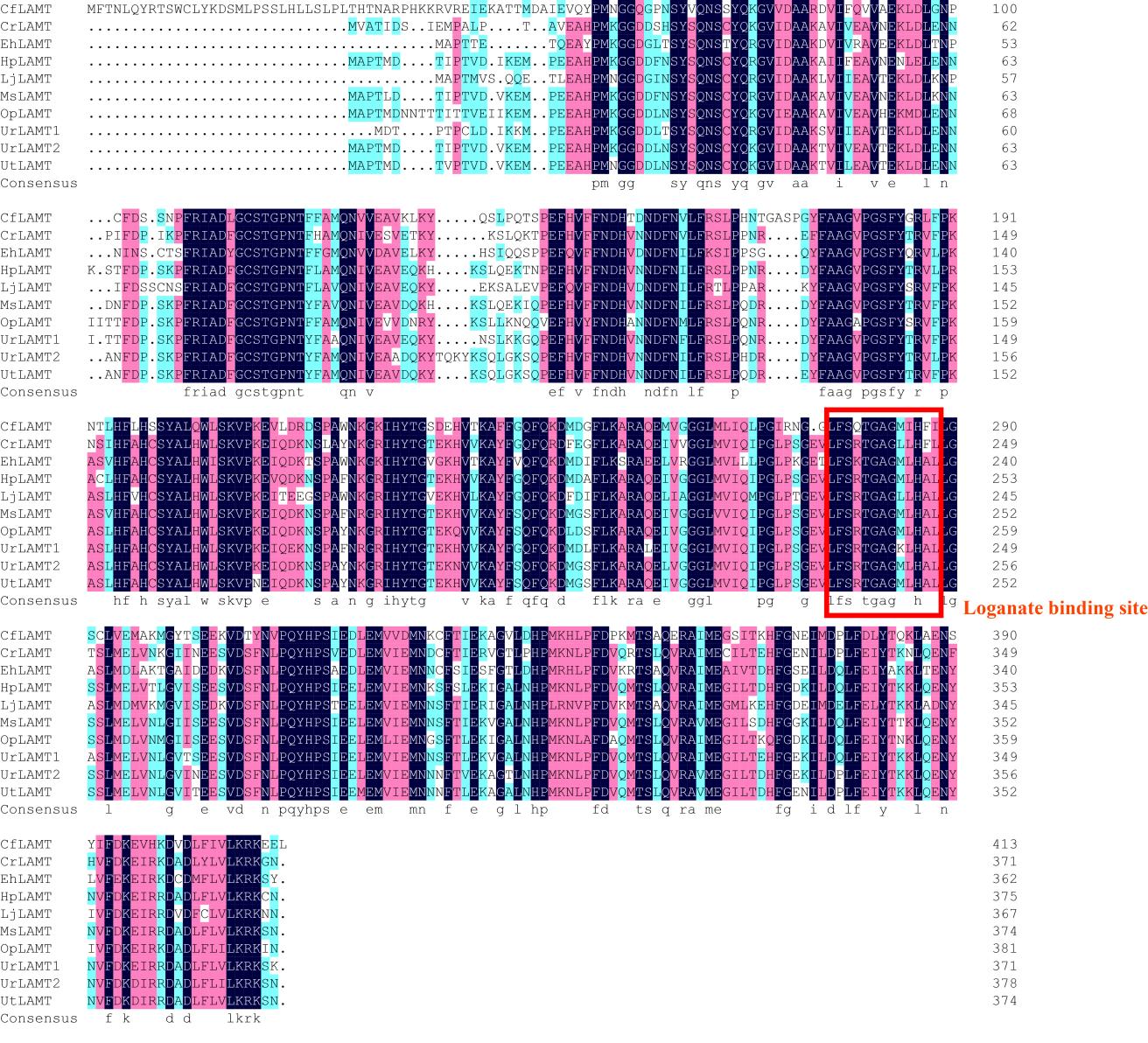


**Fig. S12.** Multiple sequences alignment of the LAMT proteins from *C. roseus*, *O. pumila*, *U. tomentosa*, *M. speciosa*, *H. patens*, *L. japonica*, *C. florida*, *E. herrerae*, *U. rhynchophylla* using DNAMAN7.0 software. The highly conserved domain in each group is in dark blue. The red square represented the Loganate bingding site.


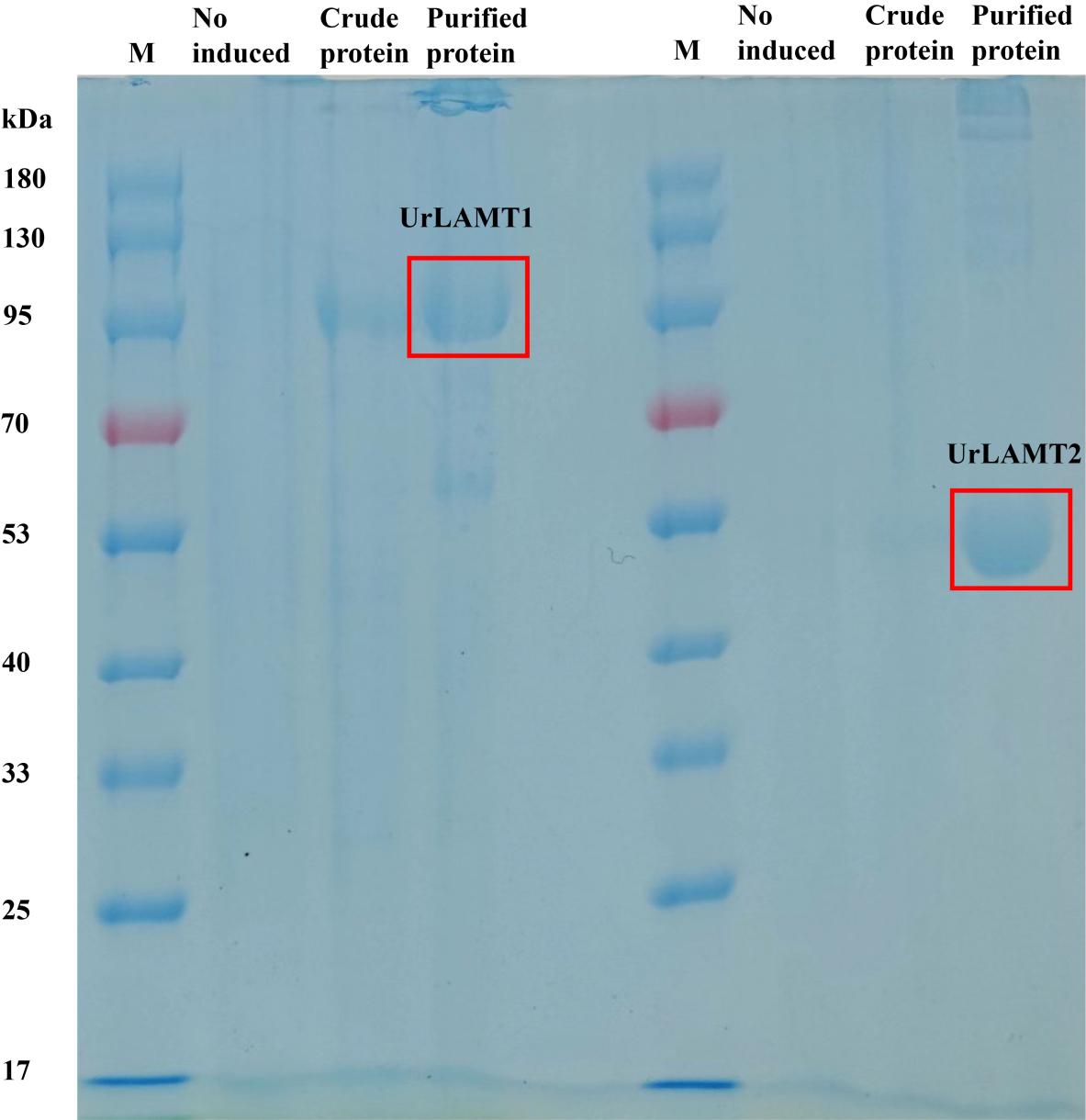


**Fig. S13.** The SDS-PAGE of uninduced, crude, and purified UrLAMT1 and UrLAMT2. The square represented the target protein.


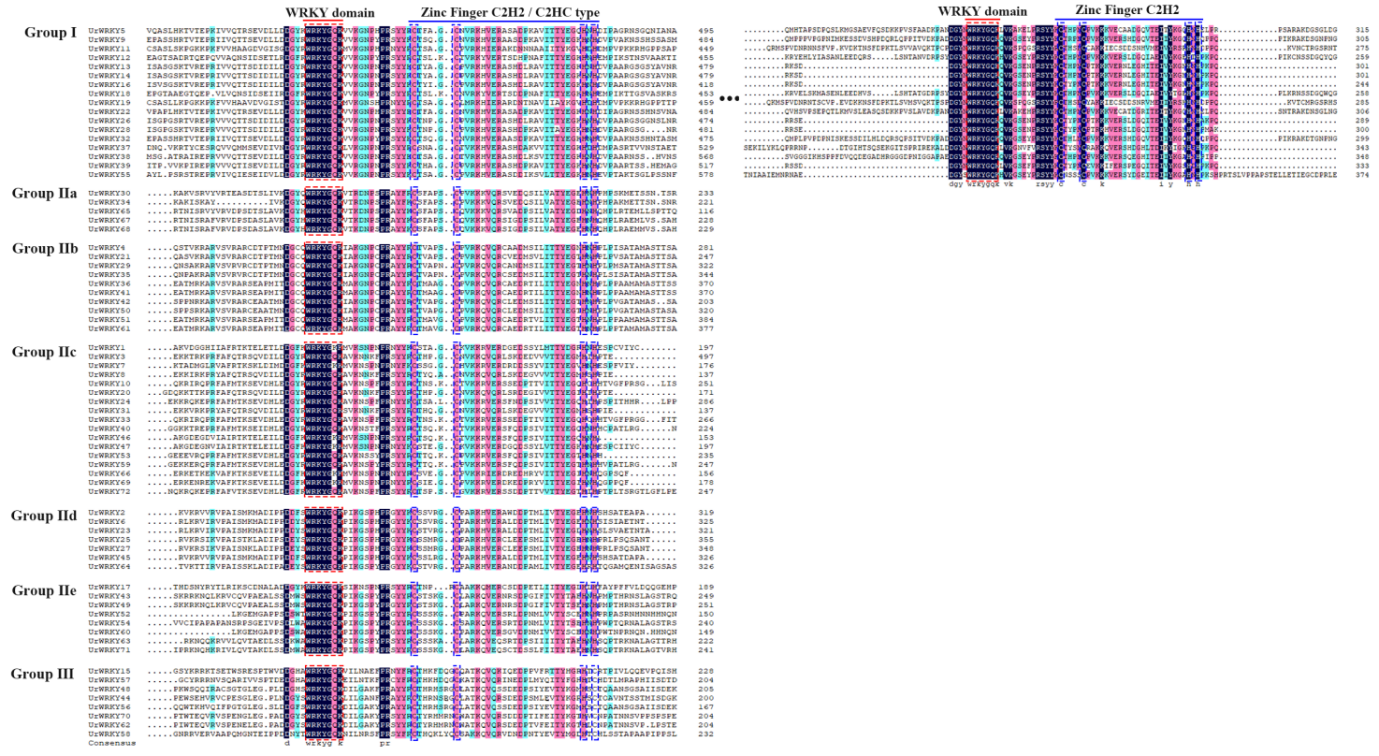


**Fig. S14.** Multiple sequences alignment of the 72 UrWRKY proteins using DNAMAN7.0 software. The highly conserved domain in each group is in dark blue. The red lines represent the WRKY domains, and the blue lines indicated the C_2_H_2_ or C_2_HC zinc finger motifs.


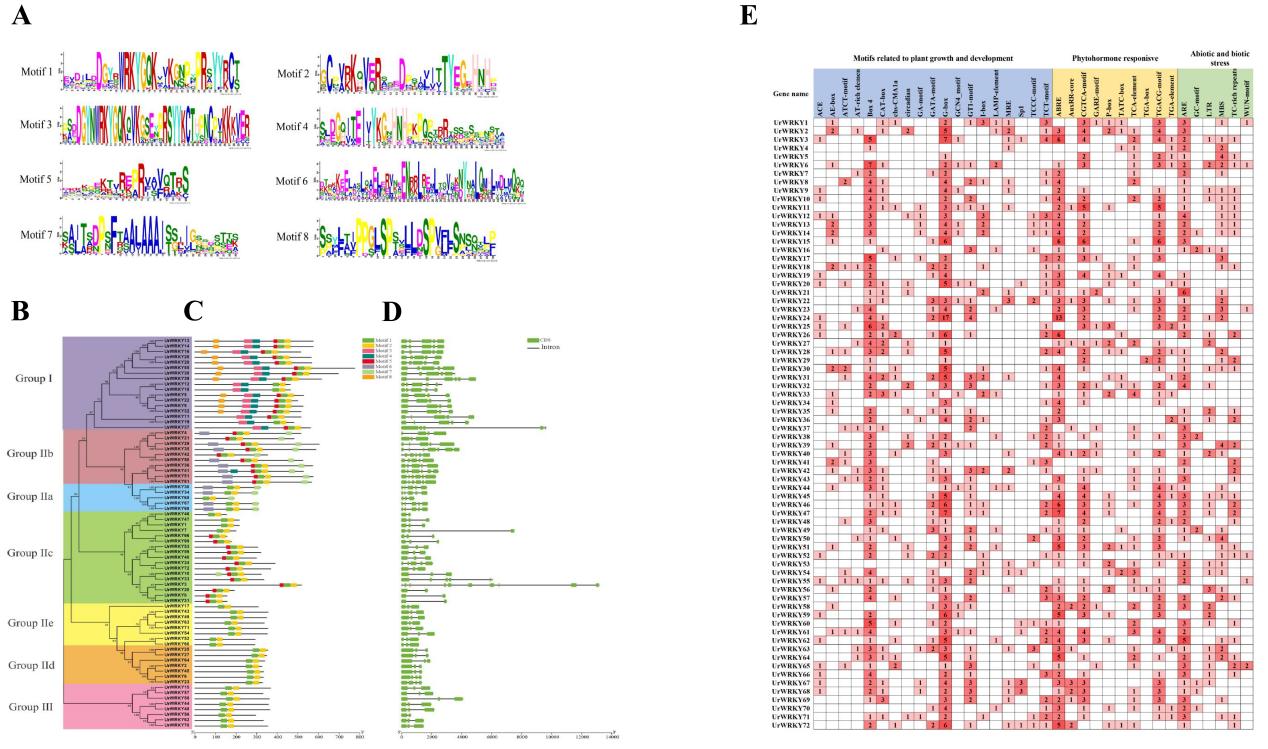


**Fig. S15.** Conserved motifs, gene structure, and *cis*-acting elements analysis of *UrWRKY* genes. **A** Sequence logos for motif 1-8. **B** The phylogenetic tree of UrWRKY protein was constructed using MEGA7.0. **C** Motif compositions of UrWRKY proteins. Eight motifs are represented by different colored boxes with numbered 1-8. **D** Exon-intron structures analysis of *UrWRKY* genes. **E** *Cis*-acting elements analysis in the *UrWRKY* promoter region were carried out. The number of *cis*-acting elements are presented in numerical form. The greater the quantity, the darker the color. Blue, yellow, green represent three categories of cis-acting elements related to plant growth and development, phytohormone responsive, and abiotic and biotic stress.


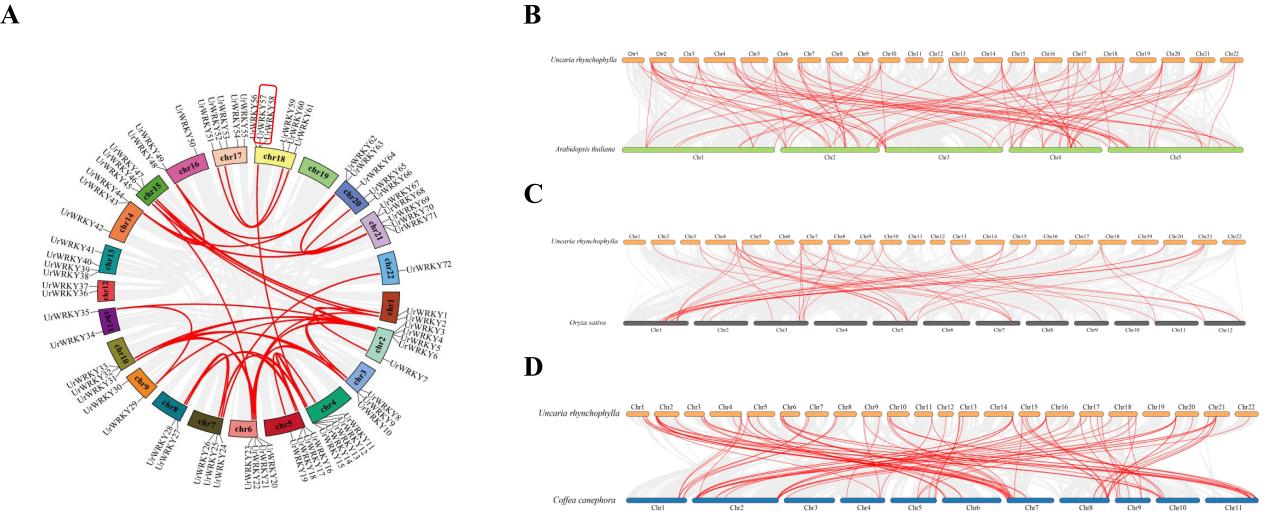


**Fig. S16.**  Collinearity analysis of the *UrWRKY* gene family in the *U. rhynchophylla* genome assembly. **A** Chromosomal distribution and syntenic relationships of *UrWRKY* genes. Chromosomal locations and syntenic relationships are depicted in a circular diagram. Gray lines represent all syntenic blocks in the *U. rhynchophylla* genome, while red lines indicate segmentally duplicated *UrWRKY* gene pairs. Tandemly duplicated genes are marked with red boxes. Chromosomes 1-22 are color-coded for clarity. **B**-**D** Comparative synteny of *WRKY* genes between *U. rhynchophylla* and three representative species: *A. thaliana*, *O. sativa*, and *C. camephora*. Collinear *UrWRKY* gene pairs are highlighted in red within gray syntenic blocks.


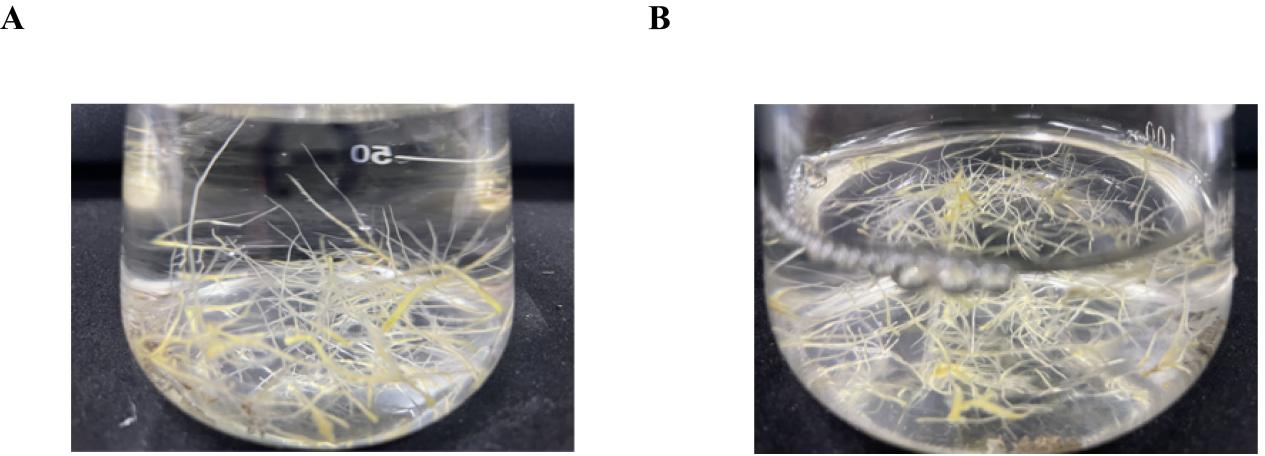


**Fig. S17.** **A** The phenotype of the *pBI121-EV* transgenic hairy roots. **B** The phenotype of the *UrWRKY37*-*OE* transgenic hairy roots.
