# Supplementary material for: The genome of the medicinal plant Uncaria rhynchophylla provides new insights into monoterpenoid indole alkaloid metabolism and its molecular regulatory mechanism
Source: Mol Hortic. 2026 Feb 3;6:9. doi: 10.1186/s43897-025-00187-7 (PMC12866418; doi:10.1186/s43897-025-00187-7)
Supplement: Supplementary file 2 — Supplementary Material 2. Supplemental material 2: Table S1. Statistics of Illumina Novaseq sequencing data. Table S2. K-mer analysis data statistics. Table S3. Statistical of the assembly results of the U. rhynchophylla genome. Table S4. The 22 chromosomes assembled of U. rhynchophylla genome based on Hi-C sequencing. Table S5. The summary of BUSCO evaluation of genome assembly. Table S6. The statistics of coding gene prediction results. Table S7. The statistics of coding gene prediction results. Table S8. The statistics of repeated sequence annotation. Table S9. Comparisons of genes and gene families among plant species we investigated. Table S10. The statistics of RIN and IRN biosynthesis genes. Table S11. The statistics of CYP450 genes in U. rhynchophylla genome. Table S12. The detailed information of WRKY transcription factor family in U. rhynchophylla. Table S13. The syntenic analysis of WRKY genes between U. rhynchophylla and A.thaliana,O.sativa, and C. camephora. [file 43897_2025_187_MOESM2_ESM.docx]

**Supplementary material 2**

**Table S1**. Statistics of Illumina Novaseq sequencing data

| Library | Length (bp) | Raw Reads | Clean Reads | Raw Base  (G) | Clean Base  (G) | Q20 (%) | Q30 (%) | GC Content (%) |
| --- | --- | --- | --- | --- | --- | --- | --- | --- |
| GT_BDSW190059509 | 150 | 325,608,548 | 325,602,572 | 45.48 | 45.48 | 90.86 | 90.86 | 36.39 |
| GT_BDSW192039481 | 150 | 162,193,306 | 162,192,094 | 22.65 | 22.65 | 90.92 | 90.92 | 36.73 |

**Table S2**. K-mer analysis data statistics

| Item | Number |
| --- | --- |
| Heterozygosity | 1.06% |
| Genome Haploid Length | 621,623,502 bp |
| Genome Repeat Length | 324,599,322 bp |
| Genome Unique Length | 297,024,180 bp |
| Model Fit | 94.30% |
| Read Error Rate | 0.019% |

**Table S3** Statistical of the assembly results of the *U. rhynchophylla* genome

| Item | Value |
| --- | --- |
| Total_length (bp) | 627,708,771 |
| Total_length_withoutN (bp) | 627,708,771 |
| Total_number | 644 |
| GC_content (%) | 35.14 |
| N50 (bp) | 1,832,948 |
| N90 (bp) | 519,766 |
| Average (bp) | 974,703.06 |
| Median (bp) | 557,683.50 |
| Min (bp) | 10,404 |
| Max (bp) | 10,172,203 |

**Table S4** The 22 chromosomes assembled of *U. rhynchophylla* genome based on Hi-C sequencing.

| Chrosome | Length (bp) | contig_number |
| --- | --- | --- |
| Chr1 | 25,664,506 | 22 |
| Chr2 | 28,151,526 | 29 |
| Chr3 | 22,917,311 | 27 |
| Chr4 | 36,304,803 | 26 |
| Chr5 | 31,602,994 | 29 |
| Chr6 | 22,490,169 | 17 |
| Chr7 | 26,761,045 | 26 |
| Chr8 | 25,824,761 | 24 |
| Chr9 | 22,798,507 | 28 |
| Chr10 | 25,236,402 | 31 |
| Chr11 | 20,951,474 | 18 |
| Chr12 | 17,164,840 | 17 |
| Chr13 | 23,092,250 | 24 |
| Chr14 | 33,466,475 | 32 |
| Chr15 | 24,076,924 | 24 |
| Chr16 | 32,902,900 | 28 |
| Chr17 | 27,325,561 | 33 |
| Chr18 | 32,507,557 | 28 |
| Chr19 | 30,841,952 | 44 |
| Chr20 | 26,791,082 | 25 |
| Chr21 | 29,140,755 | 35 |
| Chr22 | 26,970,404 | 29 |
| Chr0 | 34,781,973 | 48 |
| Total | 627,708,771 | 644 |

**Table S5** The summary of BUSCO evaluation of genome assembly

| Item | Number | Percent (%) |
| --- | --- | --- |
| Complete BUSCOs (C) | 245 | 96.1 |
| Complete and single-copy BUSCOs (S) | 172 | 67.5 |
| Complete and duplicated BUSCOs (D) | 73 | 28.6 |
| Fragmented BUSCOs (F) | 4 | 1.6 |
| Missing BUSCOs (M) | 6 | 2.4 |
| Total BUSCO groups | 255 | 100.0 |

**Table S6** The statistics of coding gene prediction results

| Type | Number |
| --- | --- |
| the total number of gene | 46,909 |
| the average of mRNA_length | 3,396.21 |
| the average of cds_length | 1,080.93 |
| the average of exon_number | 5.08 |
| the average of exon_length | 212.93 |
| the average of intron_length | 567.44 |
| the total number of exon | 238,132 |
| the total number of intron | 191,223 |
| the total intron length | 108,507,806 |

**Table S7** The statistics of coding gene prediction results

| Item | Count | Percentage |
| --- | --- | --- |
| All | 45,320 | 100% |
| Annotation | 39,447 | 87.04% |
| Uniprot | 28,353 | 62.56% |
| Pfam | 31,565 | 69.65% |
| GO | 28,003 | 61.79% |
| KEGG | 14,303 | 31.56% |
| Pathway | 8,954 | 19.76% |
| Interproscan | 36,734 | 81.05% |
| NR | 35,020 | 77.27% |

**Table S8** The statistics of repeated sequence annotation

| Item | Number | Length (bp) | Coverage (%) |
| --- | --- | --- | --- |
| SINE | 54 | 3,641 | 0.00% |
| LINE | 18,743 | 16,066,319 | 2.53% |
| LTR | 190,392 | 218,318,860 | 34.35% |
| DNA | 36,892 | 14,088,270 | 2.22% |
| Satellite | 1,036 | 249,137 | 0.04% |
| Simple_repeat | 113,789 | 5,393,467 | 0.85% |
| Low_complexity | 25,368 | 1,246,594 | 0.20% |
| Other | 3,028 | 2,084,365 | 0.33% |
| Unknown | 197,573 | 77,383,890 | 12.18% |
| Total | 586,875 | 325,361,517 | 51.19% |

| Species | Genes number | Genes in families | Unclustered genes | Family number | Unique families | Average genes per family |
| --- | --- | --- | --- | --- | --- | --- |
| *A.thaliana* | 27,562 | 22,989 | 4,573 | 14,368 | 916 | 1.6 |
| *C.roseus* | 34,363 | 28,894 | 5,469 | 15,058 | 655 | 1.92 |
| *C.arabica* | 44,759 | 41,319 | 3,440 | 17,600 | 966 | 2.35 |
| *C.sativus* | 20,040 | 18,743 | 1,297 | 14,714 | 198 | 1.27 |
| *E.californica* | 93,339 | 78,234 | 15,105 | 17,554 | 3,315 | 4.46 |
| *G.elegans* | 26,768 | 20,320 | 6,448 | 16,789 | 644 | 1.21 |
| *G.jasminoides* | 35,954 | 25,065 | 10,889 | 17,677 | 1,288 | 1.42 |
| *G.sempervirens* | 22,617 | 18,152 | 4,465 | 15,474 | 154 | 1.17 |
| *G.max* | 46,964 | 42,705 | 4,259 | 16,328 | 1,597 | 2.62 |
| *L.japonica* | 33,961 | 25,569 | 8,392 | 14,740 | 1,103 | 1.73 |
| *M.cordata* | 21,911 | 18,816 | 3,095 | 14,600 | 303 | 1.29 |
| *N.tabacum* | 61,780 | 52,986 | 8,794 | 19,573 | 1,760 | 2.71 |
| *O.sativa* | 39,049 | 26,039 | 13,010 | 14,212 | 2,227 | 1.83 |
| *P.somniferum* | 63,018 | 56,798 | 6,220 | 17,474 | 2,938 | 3.25 |
| *S.grosvenori* | 30,565 | 21,899 | 8,666 | 13,193 | 1,559 | 1.66 |
| *S.lycopersicum* | 25,613 | 22,684 | 2,929 | 16,844 | 287 | 1.35 |
| *U.rhynchophylla* | 45,320 | 33,076 | 12,244 | 18,317 | 2,020 | 1.81 |
| *V.vinifera* | 25,834 | 23,345 | 2,489 | 15,735 | 442 | 1.48 |

**Table S9** Comparisons of genes and gene families among plant species we investigated

**Table S10** The statistics of RIN and IRN biosynthesis genes

| Gene name | Enzyme Name | Number |
| --- | --- | --- |
| AACT | Acetyl-CoA: acetyl-CoA C-acetyltransferase | 2 |
| HMGS | 3-hydroxy-3-methylglutaryl-CoA synthase | 1 |
| HMGR | 3-hydroxy-3-methylglutaryl-CoA reductase | 2 |
| MK | Mevalonate kinase | 1 |
| PMK | Phosphomevalonate kinase | 2 |
| MDC | Mevalonate 5-diphosphate decarboxylase | 1 |
| DXS | 1-deoxy-D-xylulose 5-phosphate synthase 2 | 3 |
| DXR | 1-deoxy-D-xylulose-5-phosphate reductoisomerase | 1 |
| CMS | 4-(cytidine 5-diphospho)-2-C-methylerythritol synthase | 1 |
| CMK | 4-(cytidine 5-diphospho)-2-C-methylerythritolkinase | 1 |
| MECS | 2-C-methylerythritol-2,4-cyclodiphosphate synthase | 1 |
| HDS | Hydroxy methyl butenyl 4-diphosphate synthase | 1 |
| HDR | 1-hydroxy-2-methyl-butenyl 4-diphosphate reductase | 1 |
| IPPI(IPI) | Isopentenyl diphosphate isomerase | 1 |
| FPS | farnesyl pyrophosphate synthase | 2 |
| GES | Geraniol synthase | 1 |
| G10H | Geraniol 10-hydroxylase | 1 |
| 10-HGO | 10-hydroxygeraniol oxidoreductase | 2 |
| IS | Iridoid synthase | 2 |
| IO | Iridoid oxidase | 1 |
| 7-DLGT | 7-deoxyloganetic acid UDP-glucosyltransferase | 1 |
| 7-DLH | 7-deoxyloganic acid hydroxylase | 3 |
| LAMT | Loganic acid methyltransferase | 2 |
| SLS | Secologanin synthase | 6 |
| STR | Strictosidine synthase | 2 |
| SGD | Strictosidine-β-D-glucosidase | 6 |
| AS | Anthranilate synthase alpha | 3 |
| AnPRT | Anthranilate phosphoribosyl transferase | 1 |
| PAI | Phosphoribosyl anthranilate isomerase | 1 |
| IGPS | Indole-3-glycerol phosphate synthase | 3 |
| TSA/TSB | Tryptophan synthase | 1/3 |
| TDC | Tryptophan decarboxylase | 3 |

**Table S11** The statistics of *CYP450* genes in *U. rhynchophylla* genome

| Gene id | Category |
| --- | --- |
| g14729.t1 | cytochrome P450 81D1-like |
| g16422.t2 | cytochrome P450 CYP72A219-like |
| g6659.t2 | cytochrome P450 84A1-like |
| g16422.t1 | cytochrome P450 CYP72A219-like |
| novel133.t1 | cytochrome P450 71A8-like |
| g41064.t2 | cytochrome P450 704C1-like |
| g23287.t2 | cytochrome P450 CYP72A219-like |
| g25482.t1 | cytochrome P450 CYP72A219-like |
| g23288.t1 | cytochrome P450 CYP72A219-like |
| g23290.t1 | cytochrome P450 CYP72A219-like |
| g36738.t1 | cytochrome P450 71D10-like |
| g21575.t1 | cytochrome P450 94C1 |
| g31056.t1 | cytochrome P450 CYP72A219-like |
| g20704.t1 | cytochrome P450 714C2-like |
| g25482.t2 | cytochrome P450 CYP72A219-like |
| g25325.t1 | cytochrome P450 71A1-like |
| g7826.t1 | cytochrome P450 76AD1-like |
| g28409.t4 | cytochrome P450 71A1-like |
| g16434.t5 | cytochrome P450 CYP72A219-like |
| g23276.t1 | cytochrome P450 CYP72A219-like |
| g12615.t1 | cytochrome P450 81D1-like |
| g41767.t1 | cytochrome P450 71A1-like |
| g34728.t1 | cytochrome P450 714C2-like |
| g41064.t1 | cytochrome P450 704C1-like |
| g16432.t1 | cytochrome P450 CYP72A219-like |
| g6225.t1 | PREDICTED: cytochrome P450 71A1 |
| g37005.t1 | cytochrome P450 CYP736A12-like |
| novel528.t1 | cytochrome P450 71A8-like |
| g27958.t4 | cytochrome P450 71D10-like |
| g7585.t2 | cytochrome P450 71A8-like |
| g33906.t1 | cytochrome P450 85A1-like |
| g16432.t2 | cytochrome P450 CYP72A219-like isoform X1 |
| g659.t2 | cytochrome P450 98A2-like |
| g20100.t2 | cytochrome P450 81E8-like, partial |
| novel2042.t1 | cytochrome P450 84A1 |
| g16422.t6 | cytochrome P450 CYP72A219-like |
| g36491.t1 | Cytochrome P450 71A1 like |
| g37180.t2 | cytochrome P450 94B3-like |
| g33130.t3 | cytochrome P450 81F3-like |
| g27190.t1 | cytochrome P450 98A2-like |
| g4366.t3 | cytochrome P450 76AD1-like |
| g16422.t4 | cytochrome P450 CYP72A219-like |
| g16434.t6 | cytochrome P450 CYP72A219-like |
| g5084.t2 | cytochrome P450 86A22 |
| g14719.t1 | cytochrome P450 81E8-like |
| g19932.t1 | cytochrome P450 85A1-like |
| g33128.t1 | cytochrome P450 81F3-like |
| g14725.t1 | cytochrome P450 81D1-like |
| g22497.t2 | cytochrome P450 81D1-like |
| g34729.t2 | cytochrome P450 714C2-like |
| g16424.t1 | cytochrome P450 CYP72A219-like |
| g22496.t2 | cytochrome P450 81E8-like |
| g32975.t3 | NADPH--cytochrome P450 reductase-like |
| g3218.t3 | cytochrome P450 CYP749A22-like isoform X1 |
| g12904.t2 | cytochrome P450 94A1-like |
| g16434.t4 | cytochrome P450 CYP72A219-like |
| g25422.t2 | cytochrome P450 711A1-like isoform X1 |
| novel131.t1 | cytochrome P450 71A4-like |
| g31061.t2 | cytochrome P450 CYP72A219-like |
| g43001.t2 | cytochrome P450 76A1-like |
| g28189.t1 | cytochrome P450 734A1-like |
| g6222.t1 | Cytochrome P450 71A1 like |
| g14718.t1 | cytochrome P450 81D1-like |
| g37009.t1 | cytochrome P450 CYP736A12-like |
| g40409.t1 | cytochrome P450 81E8-like, partial |
| g4317.t1 | PREDICTED: cytochrome P450 87A3-like |
| g40785.t1 | cytochrome P450 90A1-like |
| g22997.t1 | cytochrome P450 86B1-like |
| g22497.t4 | cytochrome P450 81D1-like |
| g16434.t7 | cytochrome P450 CYP72A219-like |
| g16422.t3 | cytochrome P450 CYP72A219-like |
| novel132.t1 | cytochrome P450 71A3-like |
| g28664.t1 | cytochrome P450 CYP736A12-like |
| g37015.t1 | cytochrome P450 CYP736A12-like |
| g7585.t1 | cytochrome P450 71A2-like |
| g39891.t1 | cytochrome P450 87A3-like |
| g44070.t1 | cytochrome P450 98A2-like |
| g33129.t1 | cytochrome P450 81F3-like |
| g39897.t1 | cytochrome P450 87A3-like |
| g13716.t1 | cytochrome P450 704C1-like |
| g14439.t1 | cytochrome P450 77A1 |
| g31767.t1 | cytochrome P450 714A1-like |
| g18344.t1 | cytochrome P450 71A2-like |
| g31062.t1 | cytochrome P450 CYP72A219-like |
| g24833.t1 | PREDICTED: cytochrome P450 714C2-like |
| g16440.t2 | cytochrome P450 CYP72A219-like |
| g5084.t1 | cytochrome P450 86A22 |
| g24277.t1 | cytochrome P450 94C1-like |
| g25422.t1 | cytochrome P450 711A1-like isoform X1 |
| g14844.t1 | cytochrome P450 94C1 |
| g6735.t1 | cytochrome P450 90B1 |
| g16571.t1 | cytochrome P450 89A2-like |
| g42131.t1 | cytochrome P450 83B1-like |
| g8536.t1 | cytochrome P450 94C1 |
| g33130.t1 | cytochrome P450 81F3-like |
| g16434.t8 | cytochrome P450 CYP72A219-like isoform X1 |
| g34719.t1 | cytochrome P450 714C2-like |
| g23288.t2 | cytochrome P450 CYP72A219-like |
| g16442.t2 | cytochrome P450 CYP72A219-like |
| g8251.t1 | cytochrome P450 CYP736A12-like |
| g12160.t1 | cytochrome P450 86A22 |
| g3218.t2 | cytochrome P450 CYP749A22-like isoform X1 |
| g42215.t3 | NADPH--cytochrome P450 reductase |
| g37182.t1 | cytochrome P450 94B3-like |
| g31359.t1 | cytochrome P450 77A2-like |
| g23286.t2 | cytochrome P450 CYP72A219-like |
| g37983.t2 | cytochrome P450 71A1-like |
| g14717.t1 | cytochrome P450 81D1-like |
| g31063.t2 | cytochrome P450 CYP72A219-like |
| g15719.t2 | cytochrome P450 78A5-like |
| g33695.t1 | cytochrome P450 81E8-like |
| g33336.t2 | cytochrome P450 84A1 |
| g42215.t2 | NADPH--cytochrome P450 reductase |
| g14730.t1 | cytochrome P450 81E8-like |
| g25325.t2 | cytochrome P450 71A1-like |
| g22677.t1 | cytochrome P450 734A1-like |
| g35554.t1 | cytochrome P450 81E8-like |
| g31359.t2 | cytochrome P450 77A2-like |
| g11599.t4 | cytochrome P450 71A2-like |
| g2739.t1 | cytochrome P450 86A1 |
| g30231.t1 | cytochrome P450 734A1-like |
| g340.t1 | cytochrome P450 711A1-like isoform X2 |
| g11599.t3 | cytochrome P450 71A2-like |
| g31456.t1 | cytochrome P450 87A3-like isoform X2 |
| g9080.t2 | cytochrome P450 714C2-like |
| g7815.t1 | cytochrome P450 76AD1-like |
| g16442.t3 | cytochrome P450 CYP72A219-like |
| g11599.t2 | cytochrome P450 71A2-like |
| g2924.t1 | cytochrome P450 76AD1-like |
| g16421.t1 | cytochrome P450 CYP72A219-like |
| g40785.t2 | cytochrome P450 90A1-like |
| g25482.t3 | cytochrome P450 CYP72A219-like |
| g34729.t3 | cytochrome P450 714C2-like |
| g24823.t1 | PREDICTED: cytochrome P450 714C2-like |
| g28409.t3 | Cytochrome P450 71A1 |
| g27958.t3 | cytochrome P450 71D10-like |
| g35003.t1 | cytochrome P450 94A1-like |
| g23286.t1 | cytochrome P450 CYP72A219-like |
| g33693.t2 | cytochrome P450 81E8-like, partial |
| novel2829.t1 | cytochrome P450 71A1-like |
| g6660.t1 | cytochrome P450 84A1 |
| g8246.t1 | cytochrome P450 CYP736A12-like |
| g11599.t1 | cytochrome P450 71A2-like |
| g23287.t1 | cytochrome P450 CYP72A219-like |
| g42215.t1 |  |
| g14737.t1 | cytochrome P450 81D1-like |
| g4098.t1 | cytochrome P450 78A9-like |
| g6659.t1 | cytochrome P450 84A1-like |
| g40243.t1 | cytochrome P450 71A1-like |
| g35002.t1 | cytochrome P450 94A1-like |
| g33336.t1 | cytochrome P450 84A1-like |
| g23282.t1 | cytochrome P450 CYP72A219-like |
| g36567.t1 | cytochrome P450 94C1 |
| g33130.t4 | cytochrome P450 81F3-like |
| g30278.t1 | cytochrome P450 CYP82D47-like |
| g31056.t2 | cytochrome P450 CYP72A219-like |
| g41443.t1 | cytochrome P450 76AD1-like |
| g41064.t3 | cytochrome P450 704C1-like |
| g2127.t1 | cytochrome P450 78A5-like |
| g24826.t1 | PREDICTED: cytochrome P450 714C2-like |
| g33426.t1 | cytochrome P450 90B1 |
| g37729.t1 | cytochrome P450 734A1-like |
| g3218.t1 | cytochrome P450 CYP749A22-like isoform X1 |
| g31657.t1 | cytochrome P450 90A1-like |
| g23286.t3 | cytochrome P450 CYP72A219-like |
| g40451.t1 | cytochrome P450 77A2-like |
| g16439.t1 | cytochrome P450 CYP72A219-like |
| g43001.t1 | cytochrome P450 76A1-like |
| g23286.t4 | cytochrome P450 CYP72A219-like |
| g23289.t1 | cytochrome P450 CYP72A219-like |
| g37017.t1 | cytochrome P450 CYP736A12-like |
| g14701.t1 | cytochrome P450 86A1 |
| g16442.t1 | cytochrome P450 CYP72A219-like |
| g16425.t1 | cytochrome P450 CYP72A219-like |
| g12904.t3 | cytochrome P450 94A1-like |
| g5084.t3 | cytochrome P450 86A22 |
| g32249.t1 | cytochrome P450 89A2-like |
| g340.t2 | cytochrome P450 711A1-like |
| g6058.t1 | cytochrome P450 714C2-like |
| g16422.t5 | cytochrome P450 CYP72A219-like |
| g34728.t2 | cytochrome P450 714C2-like |
| g6659.t3 | cytochrome P450 84A1 |
| g9080.t1 | cytochrome P450 714C2-like |
| g40927.t1 | cytochrome P450 714A1-like |
| g31055.t1 | cytochrome P450 CYP72A219-like isoform X1 |
| g41766.t1 | cytochrome P450 71A1-like |
| g34729.t1 | cytochrome P450 714C2-like |
| g31060.t1 | cytochrome P450 CYP72A219-like |
| g36483.t1 | Cytochrome P450 71A1 like |
| g7584.t1 | cytochrome P450 71A1-like |
| g16439.t2 | cytochrome P450 CYP72A219-like |
| g31061.t1 | cytochrome P450 CYP72A219-like |
| g37180.t1 | cytochrome P450 94B3-like |
| g18343.t1 | cytochrome P450 71A2-like |
| novel131.t2 | cytochrome P450 71A3-like |
| g653.t2 | cytochrome P450 94A1-like |
| g37012.t1 | cytochrome P450 CYP736A12-like |
| g36486.t1 | cytochrome P450 71A1-like |
| g6661.t1 | cytochrome P450 84A1 |
| g27191.t3 | cytochrome P450 98A2-like |
| g2559.t1 | cytochrome P450 CYP749A22-like |
| g15719.t1 | cytochrome P450 78A5-like |
| g31057.t1 | cytochrome P450 CYP72A219-like |
| g37983.t1 | cytochrome P450 71A1-like |
| g22497.t3 | cytochrome P450 81D1-like |
| g4248.t1 | cytochrome P450 86A8-like |
| g24830.t1 | PREDICTED: cytochrome P450 714C2-like |
| g24943.t1 | NADPH--cytochrome P450 reductase |
| g33130.t2 | cytochrome P450 81F3-like |
| g16440.t1 | cytochrome P450 CYP72A219-like |
| g31063.t3 | cytochrome P450 CYP72A219-like |
| g23279.t1 | cytochrome P450 CYP72A219-like |
| g30277.t1 | cytochrome P450 CYP82D47-like |
| g24943.t2 | NADPH--cytochrome P450 reductase |
| g14739.t1 | cytochrome P450 81D1-like |
| g22497.t5 | cytochrome P450 81D1-like |
| g16423.t1 | cytochrome P450 CYP72A219-like |
| g12615.t3 | cytochrome P450 81D1-like |
| g20100.t1 | cytochrome P450 81E8-like |

| **Table S12** The detailed information of WRKY transcription factor family in *U. rhynchophylla* | | | | | | | | |
| --- | --- | --- | --- | --- | --- | --- | --- | --- |
| Gene Name | Gene ID | Gene  Start | Gene  End | Amino acid  Length (aa) | Molecular  weight | Theoretical  pI | Instability  index | Subcellular  localization |
| UrWRKY1 | g32763.t1 | 19353280 | 19354842 | 216 | 24235.91 | 5.78 | 51.84 | Nucleus |
| UrWRKY2 | g33165.t1 | 22578579 | 22579907 | 326 | 35433.06 | 9.59 | 55.55 | Nucleus |
| UrWRKY3 | g23511.t1 | 1676276 | 1689433 | 518 | 58218.84 | 9.34 | 36.76 | Chloroplast. |
| UrWRKY4 | g19820.t1 | 2432188 | 2435156 | 515 | 56440.82 | 6.08 | 54.83 | Nucleus |
| UrWRKY5 | g19877.t1 | 2786108 | 2789309 | 528 | 57060.64 | 8.41 | 52.63 | Nucleus |
| UrWRKY6 | g20088.t1 | 4124636 | 4125956 | 332 | 37083.3 | 9.42 | 46.45 | Nucleus |
| UrWRKY7 | g11658.t1 | 23638770 | 23646270 | 199 | 22805.42 | 6.44 | 65.26 | Nucleus |
| UrWRKY8 | g2098.t1 | 19645860 | 19648769 | 156 | 17722.88 | 9.54 | 30.99 | Nucleus |
| UrWRKY9 | g2484.t1 | 22454452 | 22457787 | 525 | 56990.87 | 6.55 | 61.74 | Nucleus |
| UrWRKY10 | g2506.t1 | 22592760 | 22596092 | 323 | 35516.21 | 5.11 | 58.57 | Nucleus |
| UrWRKY11 | g25102.t1 | 26052805 | 26057636 | 516 | 55858.38 | 6.26 | 50.54 | Nucleus |
| UrWRKY12 | g24083.t1 | 34191809 | 34194496 | 462 | 51314.83 | 9.08 | 40.28 | Nucleus |
| UrWRKY13 | g23854.t1 | 35687245 | 35690072 | 575 | 63762.71 | 8.62 | 53.96 | Nucleus |
| UrWRKY14 | g17971.t1 | 35707100 | 35709927 | 575 | 63793.73 | 8.62 | 53.96 | Nucleus |
| UrWRKY15 | g18015.t1 | 36052100 | 36053989 | 368 | 40600.92 | 5.62 | 44.52 | Nucleus |
| UrWRKY16 | g43444.t1 | 510256 | 513030 | 514 | 56770.64 | 8.73 | 54.13 | Nucleus |
| UrWRKY17 | g43265.t1 | 1559877 | 1561034 | 308 | 34510.19 | 5.31 | 55.11 | Nucleus |
| UrWRKY18 | g43166.t1 | 2084201 | 2086591 | 463 | 51343.89 | 9.03 | 43.91 | Nucleus |
| UrWRKY19 | g43952.t1 | 12111208 | 12115675 | 480 | 52391.12 | 5.83 | 56.21 | Nucleus |
| UrWRKY20 | g34252.t1 | 331291 | 33302 | 192 | 21462.31 | 9.47 | 49.18 | Nucleus |
| UrWRKY21 | g34036.t1 | 1591010 | 1592780 | 481 | 52603.7 | 6.73 | 53.7 | Nucleus |
| UrWRKY22 | g33983.t1 | 1953741 | 1957015 | 500 | 54013.07 | 8.49 | 40.43 | Nucleus |
| UrWRKY23 | g33711.t1 | 3693665 | 3694993 | 328 | 35754.73 | 9.55 | 46.98 | Nucleus |
| UrWRKY24 | g35300.t1 | 316237 | 318293 | 389 | 43371.84 | 8.83 | 71.5 | Nucleus |
| UrWRKY25 | g35012.t1 | 2121170 | 2122915 | 355 | 39973.12 | 9.63 | 61.22 | Nucleus |
| UrWRKY26 | g34672.t1 | 4269466 | 4272128 | 564 | 61924.88 | 6.58 | 64.49 | Nucleus |
| UrWRKY27 | g9307.t1 | 1905662 | 1907448 | 348 | 38944.08 | 9.52 | 60.95 | Nucleus |
| UrWRKY28 | g9023.t1 | 3611025 | 3613525 | 567 | 62441.39 | 6.34 | 66.49 | Nucleus |
| UrWRKY29 | g36380.t1 | 2260062 | 2263561 | 604 | 65577.73 | 7.54 | 50.96 | Nucleus |
| UrWRKY30 | g37620.t1 | 19717878 | 19719537 | 318 | 35390.73 | 8.11 | 52.01 | Nucleus |
| UrWRKY31 | g15743.t1 | 541011 | 544024 | 156 | 17585.74 | 9.44 | 42.28 | Nucleus |
| UrWRKY32 | g15392.t1 | 3050568 | 3053985 | 516 | 56281.22 | 7.24 | 64.55 | Nucleus |
| UrWRKY33 | g15376.t1 | 3158299 | 3164355 | 335 | 37104.87 | 5.79 | 48.27 | Nucleus |
| UrWRKY34 | g28089.t1 | 2935718 | 2937415 | 306 | 33991.25 | 8.74 | 43.18 | Nucleus |
| UrWRKY35 | g21166.t1 | 19615221 | 19619072 | 588 | 63147.93 | 6.49 | 48.06 | Nucleus |
| UrWRKY36 | g3884.t1 | 8856405 | 8858832 | 572 | 61471.9 | 6.44 | 39.81 | Nucleus |
| UrWRKY37 | g2990.t1 | 12096749 | 12106385 | 562 | 62216 | 6.48 | 57.19 | Nucleus |
| UrWRKY38 | g19212.t1 | 740686 | 744241 | 696 | 75293.11 | 6.15 | 55.63 | Nucleus |
| UrWRKY39 | g19311.t1 | 1354817 | 1359752 | 615 | 66519.79 | 6.42 | 53.7 | Nucleus |
| UrWRKY40 | g16515.t1 | 4917048 | 4918997 | 299 | 33265.56 | 8.42 | 68.69 | Nucleus |
| UrWRKY41 | g42072.t1 | 15837610 | 15840053 | 527 | 56696.63 | 6.22 | 43.12 | Nucleus |
| UrWRKY42 | g1319.t1 | 4057249 | 4059111 | 354 | 38533.6 | 9.18 | 44.92 | Nucleus |
| UrWRKY43 | g16301.t1 | 31947808 | 31949268 | 356 | 39336.48 | 6.33 | 71.57 | Nucleus |
| UrWRKY44 | g16253.t1 | 32298673 | 32300640 | 357 | 39929.74 | 5.11 | 52.24 | Nucleus |
| UrWRKY45 | g22536.t1 | 3682732 | 3684084 | 333 | 36281.91 | 9.57 | 57.03 | Nucleus |
| UrWRKY46 | g22146.t1 | 7172380 | 7172979 | 153 | 17230.04 | 4.96 | 53.32 | Nucleus |
| UrWRKY47 | g39446.t1 | 9253719 | 9255553 | 216 | 24390.04 | 5.5 | 55 | Nucleus |
| UrWRKY48 | g4163.t1 | 708482 | 710657 | 364 | 40976.86 | 5.64 | 52.89 | Nucleus |
| UrWRKY49 | g4207.t1 | 1031742 | 1033277 | 348 | 38111.26 | 6.25 | 71.29 | Nucleus |
| UrWRKY50 | g13419.t1 | 27353559 | 27355691 | 524 | 57033.23 | 5.96 | 56.06 | Nucleus |
| UrWRKY51 | g14379.t1 | 1215497 | 1217769 | 575 | 62314.28 | 6.48 | 44.86 | Nucleus |
| UrWRKY52 | g35540.t1 | 7818278 | 7819402 | 293 | 33081.19 | 5.26 | 78.3 | Nucleus |
| UrWRKY53 | g6491.t1 | 12149576 | 12151369 | 305 | 34348.47 | 6.36 | 65.98 | Nucleus |
| UrWRKY54 | g9952.t1 | 20294189 | 20296383 | 352 | 38130.49 | 8.5 | 60.34 | Nucleus |
| UrWRKY55 | g10775.t1 | 25332839 | 25336340 | 776 | 85114.99 | 6.18 | 52.13 | Nucleus |
| UrWRKY56 | g41239.t1 | 1164683 | 1165298 | 167 | 18675.03 | 6.66 | 48.32 | Nucleus |
| UrWRKY57 | g40903.t1 | 3286242 | 3288329 | 330 | 37140.7 | 6.21 | 55.98 | Nucleus |
| UrWRKY58 | g40902.t1 | 3290459 | 3294536 | 361 | 38602.15 | 7.72 | 51.34 | Nucleus |
| UrWRKY59 | g41619.t1 | 22187723 | 22189307 | 320 | 36244.65 | 6.87 | 64.02 | Nucleus |
| UrWRKY60 | g9712.t1 | 25587090 | 25588234 | 291 | 32766.07 | 5.26 | 65.72 | Nucleus |
| UrWRKY61 | g7180.t1 | 30962578 | 30964854 | 566 | 61114.35 | 6.53 | 45.6 | Nucleus |
| UrWRKY62 | g5188.t1 | 858854 | 860311 | 332 | 37416.47 | 5.45 | 53.2 | Nucleus |
| UrWRKY63 | g5121.t1 | 1284888 | 1286112 | 337 | 37732.86 | 6.01 | 59.94 | Nucleus |
| UrWRKY64 | g21042.t1 | 10867579 | 10869477 | 340 | 36408.19 | 9.46 | 41.68 | Nucleus |
| UrWRKY65 | g1969.t1 | 20235816 | 20236653 | 191 | 20854.15 | 8.75 | 52.68 | Nucleus |
| UrWRKY66 | g315.t1 | 25407719 | 25409894 | 156 | 17974.71 | 6.31 | 45.97 | Nucleus |
| UrWRKY67 | g1935.t1 | 4886210 | 4887942 | 309 | 34347.26 | 5.95 | 43.98 | Nucleus |
| UrWRKY68 | g16165.t1 | 5172003 | 5173732 | 310 | 34523.49 | 6.1 | 42.36 | Nucleus |
| UrWRKY69 | g25449.t1 | 11111914 | 11114389 | 178 | 20454.17 | 6.07 | 44.95 | Nucleus |
| UrWRKY70 | g12066.t1 | 13554116 | 13555569 | 355 | 39863.83 | 5.17 | 60.64 | Nucleus |
| UrWRKY71 | g12124.t1 | 13943726 | 13945155 | 355 | 39451.97 | 5.9 | 56.67 | Nucleus |
| UrWRKY72 | g17513.t1 | 16625860 | 16627443 | 369 | 40281.91 | 5.43 | 68.96 | Nucleus |

**Table S13** The syntenic analysis of *WRKY* genes between *U. rhynchophylla* and *A.thaliana*, *O.sativa*, and *C. camephora*

| Gene_ID | Gene_name | Ur_Chrom | Gene_ID | Chrom | Gene_name | Species | Type |
| --- | --- | --- | --- | --- | --- | --- | --- |
| g33165.t1 | *UrWRKY2* | chr1 | AT2G23320.1.TAIR10 | Chr2 | *AtWRKY15* | *Arabidopsis thaliana* | IId |
| g32763.t1 | *UrWRKY1* | chr1 | AT5G64810.1.TAIR10 | Chr5 | *AtWRKY51* | *Arabidopsis thaliana* | IIc |
| g19820.t1 | *UrWRKY4* | chr2 | AT1G69810.1.TAIR10 | Chr1 | *AtWRKY36* | *Arabidopsis thaliana* | IIb |
| g19877.t1 | *UrWRKY5* | chr2 | AT1G13960.1.TAIR10 | Chr1 | *AtWRKY4* | *Arabidopsis thaliana* | I |
| g11658.t1 | *UrWRKY7* | chr2 | AT2G21900.1.TAIR10 | Chr2 | *AtWRKY59* | *Arabidopsis thaliana* | IIc |
| g19877.t1 | *UrWRKY5* | chr2 | AT2G03340.1.TAIR10 | Chr2 | *AtWRKY3* | *Arabidopsis thaliana* | I |
| g20088.t1 | *UrWRKY6* | chr2 | AT2G23320.1.TAIR10 | Chr2 | *AtWRKY15* | *Arabidopsis thaliana* | IIb |
| g23511.t1 | *UrWRKY3* | chr2 | AT3G01970.1.TAIR10 | Chr3 | *AtWRKY45* | *Arabidopsis thaliana* | IIc |
| g19877.t1 | *UrWRKY5* | chr2 | AT3G01080.1.TAIR10 | Chr3 | *AtWRKY58* | *Arabidopsis thaliana* | I |
| g19820.t1 | *UrWRKY4* | chr2 | AT5G15130.1.TAIR10 | Chr5 | *AtWRKY72* | *Arabidopsis thaliana* | IIb |
| g11658.t1 | *UrWRKY7* | chr2 | AT5G64810.1.TAIR10 | Chr5 | *AtWRKY51* | *Arabidopsis thaliana* | IIc |
| g23511.t1 | *UrWRKY3* | chr2 | AT5G13080.1.TAIR10 | Chr5 | *AtWRKY75* | *Arabidopsis thaliana* | IIc |
| g2506.t1 | *UrWRKY10* | chr3 | AT1G69310.1.TAIR10 | Chr1 | *AtWRKY57* | *Arabidopsis thaliana* | IIc |
| g2484.t1 | *UrWRKY9* | chr3 | AT1G13960.1.TAIR10 | Chr1 | *AtWRKY4* | *Arabidopsis thaliana* | I |
| g2484.t1 | *UrWRKY9* | chr3 | AT2G03340.1.TAIR10 | Chr2 | *AtWRKY3* | *Arabidopsis thaliana* | I |
| g2484.t1 | *UrWRKY9* | chr3 | AT3G01080.1.TAIR10 | Chr3 | *AtWRKY58* | *Arabidopsis thaliana* | I |
| g2098.t1 | *UrWRKY8* | chr3 | AT5G13080.1.TAIR10 | Chr5 | *AtWRKY75* | *Arabidopsis thaliana* | IIc |
| g24083.t1 | *UrWRKY12* | chr4 | AT2G37260.1.TAIR10 | Chr2 | *AtWRKY44* | *Arabidopsis thaliana* | I |
| g23854.t1 | *UrWRKY13* | chr4 | AT2G30250.1.TAIR10 | Chr2 | *AtWRKY25* | *Arabidopsis thaliana* | I |
| g23854.t1 | *UrWRKY13* | chr4 | AT2G38470.1.TAIR10 | Chr2 | *AtWRKY33* | *Arabidopsis thaliana* | I |
| g18015.t1 | *UrWRKY15* | chr4 | AT3G56400.1.TAIR10 | Chr3 | *AtWRKY70* | *Arabidopsis thaliana* | III |
| g25102.t1 | *UrWRKY11* | chr4 | AT4G30935.1.TAIR10 | Chr4 | *AtWRKY32* | *Arabidopsis thaliana* | I |
| g18015.t1 | *UrWRKY15* | chr4 | AT5G01900.1.TAIR10 | Chr5 | *AtWRKY62* | *Arabidopsis thaliana* | III |
| g43166.t1 | *UrWRKY18* | chr5 | AT2G37260.1.TAIR10 | Chr2 | *AtWRKY44* | *Arabidopsis thaliana* | I |
| g43952.t1 | *UrWRKY19* | chr5 | AT4G30935.1.TAIR10 | Chr4 | *AtWRKY32* | *Arabidopsis thaliana* | I |
| g33983.t1 | *UrWRKY22* | chr6 | AT1G13960.1.TAIR10 | Chr1 | *AtWRKY4* | *Arabidopsis thaliana* | I |
| g33983.t1 | *UrWRKY22* | chr6 | AT2G03340.1.TAIR10 | Chr2 | *AtWRKY3* | *Arabidopsis thaliana* | I |
| g33711.t1 | *UrWRKY23* | chr6 | AT2G23320.1.TAIR10 | Chr2 | *AtWRKY15* | *Arabidopsis thaliana* | IId |
| g33983.t1 | *UrWRKY22* | chr6 | AT3G01080.1.TAIR10 | Chr3 | *AtWRKY58* | *Arabidopsis thaliana* | I |
| g34252.t1 | *UrWRKY20* | chr6 | AT3G01970.1.TAIR10 | Chr3 | *AtWRKY45* | *Arabidopsis thaliana* | IIc |
| g34252.t1 | *UrWRKY20* | chr6 | AT5G13080.1.TAIR10 | Chr5 | *AtWRKY75* | *Arabidopsis thaliana* | IIc |
| g35300.t1 | *UrWRKY24* | chr7 | AT2G47260.1.TAIR10 | Chr2 | *AtWRKY23* | *Arabidopsis thaliana* | IIc |
| g35012.t1 | *UrWRKY25* | chr7 | AT2G30590.1.TAIR10 | Chr2 | *AtWRKY21* | *Arabidopsis thaliana* | IId |
| g34672.t1 | *UrWRKY26* | chr7 | AT2G30250.1.TAIR10 | Chr2 | *AtWRKY25* | *Arabidopsis thaliana* | I |
| g35300.t1 | *UrWRKY24* | chr7 | AT3G62340.1.TAIR10 | Chr3 | *AtWRKY68* | *Arabidopsis thaliana* | IIc |
| g34672.t1 | *UrWRKY26* | chr7 | AT5G07100.1.TAIR10 | Chr5 | *AtWRKY26* | *Arabidopsis thaliana* | I |
| g35300.t1 | *UrWRKY24* | chr7 | AT5G49520.1.TAIR10 | Chr5 | *AtWRKY48* | *Arabidopsis thaliana* | IIc |
| g9307.t1 | *UrWRKY27* | chr8 | AT2G30590.1.TAIR10 | Chr2 | *AtWRKY21* | *Arabidopsis thaliana* | IId |
| g9023.t1 | *UrWRKY28* | chr8 | AT2G30250.1.TAIR10 | Chr2 | *AtWRKY25* | *Arabidopsis thaliana* | I |
| g9023.t1 | *UrWRKY28* | chr8 | AT5G07100.1.TAIR10 | Chr5 | *AtWRKY26* | *Arabidopsis thaliana* | I |
| g37620.t1 | *UrWRKY30* | chr9 | AT1G80840.1.TAIR10 | Chr1 | *AtWRKY40* | *Arabidopsis thaliana* | IIa |
| g37620.t1 | *UrWRKY30* | chr9 | AT2G25000.1.TAIR10 | Chr2 | *AtWRKY60* | *Arabidopsis thaliana* | IIa |
| g15376.t1 | *UrWRKY33* | chr10 | AT1G69310.1.TAIR10 | Chr1 | *AtWRKY57* | *Arabidopsis thaliana* | IIc |
| g15392.t1 | *UrWRKY32* | chr10 | AT1G13960.1.TAIR10 | Chr1 | *AtWRKY4* | *Arabidopsis thaliana* | I |
| g15392.t1 | *UrWRKY32* | chr10 | AT2G03340.1.TAIR10 | Chr2 | *AtWRKY3* | *Arabidopsis thaliana* | I |
| g15392.t1 | *UrWRKY32* | chr10 | AT3G01080.1.TAIR10 | Chr3 | *AtWRKY58* | *Arabidopsis thaliana* | I |
| g15743.t1 | *UrWRKY31* | chr10 | AT5G13080.1.TAIR10 | Chr5 | *AtWRKY75* | *Arabidopsis thaliana* | IIc |
| g2990.t1 | *UrWRKY37* | chr12 | AT2G04880.1.TAIR10 | Chr2 | *AtWRKY1* | *Arabidopsis thaliana* | I |
| g19311.t1 | *UrWRKY39* | chr13 | AT4G26640.2.TAIR10 | Chr4 | *AtWRKY20* | *Arabidopsis thaliana* | I |
| g19212.t1 | *UrWRKY38* | chr13 | AT4G26440.1.TAIR10 | Chr4 | *AtWRKY34* | *Arabidopsis thaliana* | I |
| g19212.t1 | *UrWRKY38* | chr13 | AT5G56270.1.TAIR10 | Chr5 | *AtWRKY2* | *Arabidopsis thaliana* | I |
| g16253.t1 | *UrWRKY44* | chr14 | AT2G46400.1.TAIR10 | Chr2 | *AtWRKY46* | *Arabidopsis thaliana* | III |
| g16301.t1 | *UrWRKY43* | chr14 | AT4G23550.1.TAIR10 | Chr4 | *AtWRKY29* | *Arabidopsis thaliana* | IIe |
| g16253.t1 | *UrWRKY44* | chr14 | AT4G23810.1.TAIR10 | Chr4 | *AtWRKY53* | *Arabidopsis thaliana* | III |
| g16301.t1 | *UrWRKY43* | chr14 | AT4G01250.1.TAIR10 | Chr4 | *AtWRKY22* | *Arabidopsis thaliana* | IIe |
| g16253.t1 | *UrWRKY44* | chr14 | AT4G11070.1.TAIR10 | Chr4 | *AtWRKY41* | *Arabidopsis thaliana* | III |
| g22536.t1 | *UrWRKY45* | chr15 | AT2G23320.1.TAIR10 | Chr2 | *AtWRKY15* | *Arabidopsis thaliana* | IIb |
| g4163.t1 | *UrWRKY48* | chr16 | AT2G46400.1.TAIR10 | Chr2 | *AtWRKY46* | *Arabidopsis thaliana* | III |
| g4207.t1 | *UrWRKY49* | chr16 | AT4G01250.1.TAIR10 | Chr4 | *AtWRKY22* | *Arabidopsis thaliana* | IIe |
| g4163.t1 | *UrWRKY48* | chr16 | AT4G11070.1.TAIR10 | Chr4 | *AtWRKY41* | *Arabidopsis thaliana* | III |
| g4207.t1 | *UrWRKY49* | chr16 | AT4G23550.1.TAIR10 | Chr4 | *AtWRKY29* | *Arabidopsis thaliana* | IIe |
| g4163.t1 | *UrWRKY48* | chr16 | AT4G23810.1.TAIR10 | Chr4 | *AtWRKY53* | *Arabidopsis thaliana* | III |
| g35540.t1 | *UrWRKY52* | chr17 | AT1G29280.1.TAIR10 | Chr1 | *AtWRKY65* | *Arabidopsis thaliana* | IIe |
| g14379.t1 | *UrWRKY51* | chr17 | AT1G62300.1.TAIR10 | Chr1 | *AtWRKY6* | *Arabidopsis thaliana* | IIb |
| g9952.t1 | *UrWRKY54* | chr17 | AT1G30650.1.TAIR10 | Chr1 | *AtWRKY14* | *Arabidopsis thaliana* | IIe |
| g14379.t1 | *UrWRKY51* | chr17 | AT4G04450.1.TAIR10 | Chr4 | *AtWRKY42* | *Arabidopsis thaliana* | IIb |
| g6491.t1 | *UrWRKY53* | chr17 | AT4G18170.1.TAIR10 | Chr4 | *AtWRKY28* | *Arabidopsis thaliana* | IIc |
| g14379.t1 | *UrWRKY51* | chr17 | AT4G22070.1.TAIR10 | Chr4 | *AtWRKY31* | *Arabidopsis thaliana* | IIb |
| g6491.t1 | *UrWRKY53* | chr17 | AT5G46350.1.TAIR10 | Chr5 | *AtWRKY8* | *Arabidopsis thaliana* | IIc |
| g7180.t1 | *UrWRKY61* | chr18 | AT1G62300.1.TAIR10 | Chr1 | *AtWRKY6* | *Arabidopsis thaliana* | IIb |
| g40903.t1 | *UrWRKY57* | chr18 | AT2G40740.1.TAIR10 | Chr2 | *AtWRKY55* | *Arabidopsis thaliana* | III |
| g40903.t1 | *UrWRKY57* | chr18 | AT3G56400.1.TAIR10 | Chr3 | *AtWRKY70* | *Arabidopsis thaliana* | III |
| g7180.t1 | *UrWRKY61* | chr18 | AT4G22070.1.TAIR10 | Chr4 | *AtWRKY31* | *Arabidopsis thaliana* | IIb |
| g7180.t1 | *UrWRKY61* | chr18 | AT4G04450.1.TAIR10 | Chr4 | *AtWRKY42* | *Arabidopsis thaliana* | IIb |
| g41619.t1 | *UrWRKY59* | chr18 | AT4G18170.1.TAIR10 | Chr4 | *AtWRKY28* | *Arabidopsis thaliana* | IIc |
| g41619.t1 | *UrWRKY59* | chr18 | AT5G46350.1.TAIR10 | Chr5 | *AtWRKY8* | *Arabidopsis thaliana* | IIc |
| g40902.t1 | *UrWRKY58* | chr18 | AT5G01900.1.TAIR10 | Chr5 | *AtWRKY62* | *Arabidopsis thaliana* | III |
| g5188.t1 | *UrWRKY62* | chr20 | AT2G46400.1.TAIR10 | Chr2 | *AtWRKY46* | *Arabidopsis thaliana* | III |
| g5188.t1 | *UrWRKY62* | chr20 | AT4G11070.1.TAIR10 | Chr4 | *AtWRKY41* | *Arabidopsis thaliana* | III |
| g5121.t1 | *UrWRKY63* | chr20 | AT4G01250.1.TAIR10 | Chr4 | *AtWRKY22* | *Arabidopsis thaliana* | IIe |
| g5121.t1 | *UrWRKY63* | chr20 | AT4G23550.1.TAIR10 | Chr4 | *AtWRKY29* | *Arabidopsis thaliana* | IIe |
| g5188.t1 | *UrWRKY62* | chr20 | AT4G23810.1.TAIR10 | Chr4 | *AtWRKY53* | *Arabidopsis thaliana* | III |
| g5188.t1 | *UrWRKY62* | chr20 | AT5G24110.1.TAIR10 | Chr5 | *AtWRKY30* | *Arabidopsis thaliana* | III |
| g12066.t1 | *UrWRKY70* | chr21 | AT2G46400.1.TAIR10 | Chr2 | *AtWRKY46* | *Arabidopsis thaliana* | III |
| g12066.t1 | *UrWRKY70* | chr21 | AT4G11070.1.TAIR10 | Chr4 | *AtWRKY41* | *Arabidopsis thaliana* | III |
| g12124.t1 | *UrWRKY71* | chr21 | AT4G01250.1.TAIR10 | Chr4 | *AtWRKY22* | *Arabidopsis thaliana* | IIe |
| g12124.t1 | *UrWRKY71* | chr21 | AT4G23550.1.TAIR10 | Chr4 | *AtWRKY29* | *Arabidopsis thaliana* | IIe |
| g12066.t1 | *UrWRKY70* | chr21 | AT4G23810.1.TAIR10 | Chr4 | *AtWRKY53* | *Arabidopsis thaliana* | III |
| g25449.t1 | *UrWRKY69* | chr21 | AT5G26170.1.TAIR10 | Chr5 | *AtWRKY50* | *Arabidopsis thaliana* | IIc |
| g12066.t1 | *UrWRKY70* | chr21 | AT5G24110.1.TAIR10 | Chr5 | *AtWRKY30* | *Arabidopsis thaliana* | III |
| g17513.t1 | *UrWRKY72* | chr22 | AT2G47260.1.TAIR10 | Chr2 | *AtWRKY23* | *Arabidopsis thaliana* | IIc |
| g17513.t1 | *UrWRKY72* | chr22 | AT3G62340.1.TAIR10 | Chr3 | *AtWRKY68* | *Arabidopsis thaliana* | IIc |
| g17513.t1 | *UrWRKY72* | chr22 | AT5G49520.1.TAIR10 | Chr5 | *AtWRKY48* | *Arabidopsis thaliana* | IIc |
| g19877.t1 | *UrWRKY5* | chr2 | PAC:24113117 | Chr7 | - | *Oryza sativa* | I |
| g2506.t1 | *UrWRKY10* | chr3 | PAC:24125535 | Chr3 | - | *Oryza sativa* | IIc |
| g2484.t1 | *UrWRKY9* | chr3 | PAC:24113117 | Chr7 | - | *Oryza sativa* | I |
| g23854.t1 | *UrWRKY13* | chr4 | PAC:24121503 | Chr1 | - | *Oryza sativa* | I |
| g18015.t1 | *UrWRKY15* | chr4 | PAC:24159224 | Chr11 | - | *Oryza sativa* | III |
| g18015.t1 | *UrWRKY15* | chr4 | PAC:24145659 | Chr12 | - | *Oryza sativa* | III |
| g18015.t1 | *UrWRKY15* | chr4 | PAC:24127298 | Chr3 | - | *Oryza sativa* | III |
| g23854.t1 | *UrWRKY13* | chr4 | PAC:24154429 | Chr5 | - | *Oryza sativa* | I |
| g23854.t1 | *UrWRKY13* | chr4 | PAC:24151842 | Chr5 | - | *Oryza sativa* | I |
| g25102.t1 | *UrWRKY11* | chr4 | PAC:24101198 | Chr8 | - | *Oryza sativa* | I |
| g43444.t1 | *UrWRKY16* | chr5 | PAC:24121503 | Chr1 | - | *Oryza sativa* | I |
| g43444.t1 | *UrWRKY16* | chr5 | PAC:24151842 | Chr5 | - | *Oryza sativa* | I |
| g43444.t1 | *UrWRKY16* | chr5 | PAC:24154429 | Chr5 | - | *Oryza sativa* | I |
| g33983.t1 | *UrWRKY22* | chr6 | PAC:24113117 | Chr7 | - | *Oryza sativa* | I |
| g34672.t1 | *UrWRKY26* | chr7 | PAC:24121503 | Chr1 | - | *Oryza sativa* | I |
| g35012.t1 | *UrWRKY25* | chr7 | PAC:24148383 | Chr12 | - | *Oryza sativa* | IId |
| g35012.t1 | *UrWRKY25* | chr7 | PAC:24128365 | Chr3 | - | *Oryza sativa* | IId |
| g35012.t1 | *UrWRKY25* | chr7 | PAC:24122325 | Chr3 | - | *Oryza sativa* | IId |
| g34672.t1 | *UrWRKY26* | chr7 | PAC:24154429 | Chr5 | - | *Oryza sativa* | I |
| g9023.t1 | *UrWRKY28* | chr8 | PAC:24121503 | Chr1 | - | *Oryza sativa* | I |
| g9307.t1 | *UrWRKY27* | chr8 | PAC:24148383 | Chr12 | - | *Oryza sativa* | IId |
| g9307.t1 | *UrWRKY27* | chr8 | PAC:24128365 | Chr3 | - | *Oryza sativa* | IId |
| g9307.t1 | *UrWRKY27* | chr8 | PAC:24122325 | Chr3 | - | *Oryza sativa* | IId |
| g9023.t1 | *UrWRKY28* | chr8 | PAC:24154429 | Chr5 | - | *Oryza sativa* | I |
| g37620.t1 | *UrWRKY30* | chr9 | PAC:24129555 | Chr2 | - | *Oryza sativa* | IIa |
| g37620.t1 | *UrWRKY30* | chr9 | PAC:24141799 | Chr6 | - | *Oryza sativa* | IIa |
| g15376.t1 | *UrWRKY33* | chr10 | PAC:24125535 | Chr3 | - | *Oryza sativa* | IIc |
| g15392.t1 | *UrWRKY32* | chr10 | PAC:24113117 | Chr7 | - | *Oryza sativa* | I |
| g19212.t1 | *UrWRKY38* | chr13 | PAC:24098688 | Chr8 | - | *Oryza sativa* | I |
| g19212.t1 | *UrWRKY38* | chr13 | PAC:24138167 | Chr9 | - | *Oryza sativa* | I |
| g16253.t1 | *UrWRKY44* | chr14 | PAC:24118091 | Chr1 | - | *Oryza sativa* | III |
| g16301.t1 | *UrWRKY43* | chr14 | PAC:24133330 | Chr2 | - | *Oryza sativa* | IIe |
| g16301.t1 | *UrWRKY43* | chr14 | PAC:24154354 | Chr5 | - | *Oryza sativa* | IIe |
| g39446.t1 | *UrWRKY47* | chr15 | PAC:24116605 | Chr1 | - | *Oryza sativa* | IIc |
| g4163.t1 | *UrWRKY48* | chr16 | PAC:24118091 | Chr1 | - | *Oryza sativa* | III |
| g4207.t1 | *UrWRKY49* | chr16 | PAC:24117685 | Chr1 | - | *Oryza sativa* | IIe |
| g35540.t1 | *UrWRKY52* | chr17 | PAC:24118401 | Chr1 | - | *Oryza sativa* | IIe |
| g40903.t1 | *UrWRKY57* | chr18 | PAC:24159224 | Chr11 | - | *Oryza sativa* | III |
| g40903.t1 | *UrWRKY57* | chr18 | PAC:24145659 | Chr12 | - | *Oryza sativa* | III |
| g40903.t1 | *UrWRKY57* | chr18 | PAC:24153219 | Chr5 | - | *Oryza sativa* | III |
| g5188.t1 | *UrWRKY62* | chr20 | PAC:24118091 | Chr1 | - | *Oryza sativa* | III |
| g5121.t1 | *UrWRKY63* | chr20 | PAC:24116390 | Chr1 | - | *Oryza sativa* | IIe |
| g12124.t1 | *UrWRKY71* | chr21 | PAC:24117685 | Chr1 | - | *Oryza sativa* | IIe |
| g25449.t1 | *UrWRKY69* | chr21 | PAC:24121525 | Chr1 | - | *Oryza sativa* | IIc |
| g12066.t1 | *UrWRKY70* | chr21 | PAC:24118091 | Chr1 | - | *Oryza sativa* | III |
| g12124.t1 | *UrWRKY71* | chr21 | PAC:24154354 | Chr5 | - | *Oryza sativa* | IIe |
| g33165.t1 | *UrWRKY2* | chr1 | transcript:CDP01295 | Chr10 | - | Coffea canephora | IId |
| g32763.t1 | *UrWRKY1* | chr1 | transcript:CDP01136 | Chr10 | - | Coffea canephora | IIc |
| g33165.t1 | *UrWRKY2* | chr1 | transcript:CDP02529 | Chr7 | - | Coffea canephora | IId |
| g32763.t1 | *UrWRKY1* | chr1 | transcript:CDP12885 | Chr7 | - | Coffea canephora | IIc |
| g20088.t1 | *UrWRKY6* | chr2 | transcript:CDP01295 | Chr10 | - | Coffea canephora | IId |
| g23511.t1 | *UrWRKY3* | chr2 | transcript:CDP13366 | Chr11 | - | Coffea canephora | IIc |
| g19877.t1 | *UrWRKY5* | chr2 | transcript:CDP00386 | Chr11 | - | Coffea canephora | I |
| g19820.t1 | *UrWRKY4* | chr2 | transcript:CDP14843 | Chr4 | - | Coffea canephora | IIb |
| g19820.t1 | *UrWRKY4* | chr2 | transcript:CDP02207 | Chr7 | - | Coffea canephora | IIb |
| g19877.t1 | *UrWRKY5* | chr2 | transcript:CDP02268 | Chr7 | - | Coffea canephora | I |
| g20088.t1 | *UrWRKY6* | chr2 | transcript:CDP02529 | Chr7 | - | Coffea canephora | IId |
| g23511.t1 | *UrWRKY3* | chr2 | transcript:CDP16710 | Chr7 | - | Coffea canephora | IIc |
| g11658.t1 | *UrWRKY7* | chr2 | transcript:CDP12885 | Chr7 | - | Coffea canephora | IIc |
| g2506.t1 | *UrWRKY10* | chr3 | transcript:CDP00413 | Chr11 | - | Coffea canephora | IIc |
| g2484.t1 | *UrWRKY9* | chr3 | transcript:CDP00386 | Chr11 | - | Coffea canephora | I |
| g2098.t1 | *UrWRKY8* | chr3 | transcript:CDP13366 | Chr11 | - | Coffea canephora | IIc |
| g2484.t1 | *UrWRKY9* | chr3 | transcript:CDP02268 | Chr7 | - | Coffea canephora | I |
| g2098.t1 | *UrWRKY8* | chr3 | transcript:CDP16710 | Chr7 | - | Coffea canephora | IIc |
| g23854.t1 | *UrWRKY13* | chr4 | transcript:CDP04081 | Chr1 | - | Coffea canephora | I |
| g18015.t1 | *UrWRKY15* | chr4 | transcript:CDP13896 | Chr2 | - | Coffea canephora | III |
| g24083.t1 | *UrWRKY12* | chr4 | transcript:CDP06696 | Chr6 | - | Coffea canephora | I |
| g25102.t1 | *UrWRKY11* | chr4 | transcript:CDP10231 | Chr6 | - | Coffea canephora | I |
| g43444.t1 | *UrWRKY16* | chr5 | transcript:CDP04081 | Chr1 | - | Coffea canephora | I |
| g43265.t1 | *UrWRKY17* | chr5 | transcript:CDP06598 | Chr6 | - | Coffea canephora | IIe |
| g43952.t1 | *UrWRKY19* | chr5 | transcript:CDP10231 | Chr6 | - | Coffea canephora | I |
| g43166.t1 | *UrWRKY18* | chr5 | transcript:CDP06696 | Chr6 | - | Coffea canephora | I |
| g33711.t1 | *UrWRKY23* | chr6 | transcript:CDP01295 | Chr10 | - | Coffea canephora | IId |
| g33983.t1 | *UrWRKY22* | chr6 | transcript:CDP00386 | Chr11 | - | Coffea canephora | I |
| g34252.t1 | *UrWRKY20* | chr6 | transcript:CDP13366 | Chr11 | - | Coffea canephora | IIc |
| g33983.t1 | *UrWRKY22* | chr6 | transcript:CDP02268 | Chr7 | - | Coffea canephora | I |
| g33711.t1 | *UrWRKY23* | chr6 | transcript:CDP02529 | Chr7 | - | Coffea canephora | IId |
| g34252.t1 | *UrWRKY20* | chr6 | transcript:CDP16710 | Chr7 | - | Coffea canephora | IIc |
| g34672.t1 | *UrWRKY26* | chr7 | transcript:CDP04081 | Chr1 | - | Coffea canephora | I |
| g35012.t1 | *UrWRKY25* | chr7 | transcript:CDP03703 | Chr1 | - | Coffea canephora | IId |
| g9023.t1 | *UrWRKY28* | chr8 | transcript:CDP04081 | Chr1 | - | Coffea canephora | I |
| g9307.t1 | *UrWRKY27* | chr8 | transcript:CDP03703 | Chr1 | - | Coffea canephora | IId |
| g37620.t1 | *UrWRKY30* | chr9 | transcript:CDO98227 | Chr4 | - | Coffea canephora | IIa |
| g36380.t1 | *UrWRKY29* | chr9 | transcript:CDP14843 | Chr4 | - | Coffea canephora | IIb |
| g36380.t1 | *UrWRKY29* | chr9 | transcript:CDP02207 | Chr7 | - | Coffea canephora | IIb |
| g15376.t1 | *UrWRKY33* | chr10 | transcript:CDP00413 | Chr11 | - | Coffea canephora | IIc |
| g15392.t1 | *UrWRKY32* | chr10 | transcript:CDP00386 | Chr11 | - | Coffea canephora | I |
| g15743.t1 | *UrWRKY31* | chr10 | transcript:CDP13366 | Chr11 | - | Coffea canephora | IIc |
| g15392.t1 | *UrWRKY32* | chr10 | transcript:CDP02268 | Chr7 | - | Coffea canephora | I |
| g15743.t1 | *UrWRKY31* | chr10 | transcript:CDP16710 | Chr7 | - | Coffea canephora | IIc |
| g21166.t1 | *UrWRKY35* | chr11 | transcript:CDP14843 | Chr4 | - | Coffea canephora | IIb |
| g21166.t1 | *UrWRKY35* | chr11 | transcript:CDP02207 | Chr7 | - | Coffea canephora | IIb |
| g3884.t1 | *UrWRKY36* | chr12 | transcript:CDP10754 | Chr5 | - | Coffea canephora | IIb |
| g16515.t1 | *UrWRKY40* | chr13 | transcript:CDP06161 | Chr5 | - | Coffea canephora | IIc |
| g19311.t1 | *UrWRKY39* | chr13 | transcript:CDP13745 | Chr5 | - | Coffea canephora | I |
| g19212.t1 | *UrWRKY38* | chr13 | transcript:CDP13632 | Chr5 | - | Coffea canephora | I |
| g16301.t1 | *UrWRKY43* | chr14 | transcript:CDP08759 | Chr2 | - | Coffea canephora | IIe |
| g16253.t1 | *UrWRKY44* | chr14 | transcript:CDP08702 | Chr2 | - | Coffea canephora | III |
| g16253.t1 | *UrWRKY44* | chr14 | transcript:CDP05092 | Chr2 | - | Coffea canephora | III |
| g16301.t1 | *UrWRKY43* | chr14 | transcript:CDP05130 | Chr2 | - | Coffea canephora | IIe |
| g16301.t1 | *UrWRKY43* | chr14 | transcript:CDP12077 | Chr8 | - | Coffea canephora | IIe |
| g16253.t1 | *UrWRKY44* | chr14 | transcript:CDP12141 | Chr8 | - | Coffea canephora | III |
| g22536.t1 | *UrWRKY45* | chr15 | transcript:CDP01295 | Chr10 | - | Coffea canephora | IId |
| g22146.t1 | *UrWRKY46* | chr15 | transcript:CDP01136 | Chr10 | - | Coffea canephora | IIc |
| g39446.t1 | *UrWRKY47* | chr15 | transcript:CDP01136 | Chr10 | - | Coffea canephora | IIc |
| g22536.t1 | *UrWRKY45* | chr15 | transcript:CDP02529 | Chr7 | - | Coffea canephora | IId |
| g4163.t1 | *UrWRKY48* | chr16 | transcript:CDP05092 | Chr2 | - | Coffea canephora | III |
| g4207.t1 | *UrWRKY49* | chr16 | transcript:CDP05130 | Chr2 | - | Coffea canephora | IIe |
| g13419.t1 | *UrWRKY50* | chr16 | transcript:CDO97515 | Chr2 | - | Coffea canephora | IIb |
| g4207.t1 | *UrWRKY49* | chr16 | transcript:CDP08759 | Chr2 | - | Coffea canephora | IIe |
| g4163.t1 | *UrWRKY48* | chr16 | transcript:CDP08702 | Chr2 | - | Coffea canephora | III |
| g4207.t1 | *UrWRKY49* | chr16 | transcript:CDP12077 | Chr8 | - | Coffea canephora | IIe |
| g4163.t1 | *UrWRKY48* | chr16 | transcript:CDP12141 | Chr8 | - | Coffea canephora | III |
| g35540.t1 | *UrWRKY52* | chr17 | transcript:CDO96851 | Chr2 | - | Coffea canephora | IIe |
| g14379.t1 | *UrWRKY51* | chr17 | transcript:CDP10754 | Chr5 | - | Coffea canephora | IIb |
| g14379.t1 | *UrWRKY51* | chr17 | transcript:CDP04425 | Chr9 | - | Coffea canephora | IIb |
| g10775.t1 | *UrWRKY55* | chr17 | transcript:CDP04204 | Chr9 | - | Coffea canephora | I |
| g9952.t1 | *UrWRKY54* | chr17 | transcript:CDP12602 | Chr9 | - | Coffea canephora | IIe |
| g6491.t1 | *UrWRKY53* | chr17 | transcript:CDP06312 | Chr9 | - | Coffea canephora | IIc |
| g40903.t1 | *UrWRKY57* | chr18 | transcript:CDP13896 | Chr2 | - | Coffea canephora | III |
| g9712.t1 | *UrWRKY60* | chr18 | transcript:CDO96851 | Chr2 | - | Coffea canephora | IIe |
| g7180.t1 | *UrWRKY61* | chr18 | transcript:CDP10754 | Chr5 | - | Coffea canephora | IIb |
| g41619.t1 | *UrWRKY59* | chr18 | transcript:CDP06312 | Chr9 | - | Coffea canephora | IIc |
| g7180.t1 | *UrWRKY61* | chr18 | transcript:CDP04425 | Chr9 | - | Coffea canephora | IIb |
| g5188.t1 | *UrWRKY62* | chr20 | transcript:CDP05092 | Chr2 | - | Coffea canephora | III |
| g5121.t1 | *UrWRKY63* | chr20 | transcript:CDP05130 | Chr2 | - | Coffea canephora | IIe |
| g5121.t1 | *UrWRKY63* | chr20 | transcript:CDP08759 | Chr2 | - | Coffea canephora | IIe |
| g5188.t1 | *UrWRKY62* | chr20 | transcript:CDP08702 | Chr2 | - | Coffea canephora | III |
| g5121.t1 | *UrWRKY63* | chr20 | transcript:CDP12077 | Chr8 | - | Coffea canephora | IIe |
| g5188.t1 | *UrWRKY62* | chr20 | transcript:CDP12141 | Chr8 | - | Coffea canephora | III |
| g1969.t1 | *UrWRKY65* | chr20 | transcript:CDP17893 | Chr8 | - | Coffea canephora | IIa |
| g12066.t1 | *UrWRKY70* | chr21 | transcript:CDP05092 | Chr2 | - | Coffea canephora | III |
| g12124.t1 | *UrWRKY71* | chr21 | transcript:CDP05130 | Chr2 | - | Coffea canephora | IIe |
| g12124.t1 | *UrWRKY71* | chr21 | transcript:CDP08759 | Chr2 | - | Coffea canephora | IIe |
| g12066.t1 | *UrWRKY70* | chr21 | transcript:CDP08702 | Chr2 | - | Coffea canephora | III |
| g25449.t1 | *UrWRKY69* | chr21 | transcript:CDP08305 | Chr8 | - | Coffea canephora | IIc |
| g12124.t1 | *UrWRKY71* | chr21 | transcript:CDP12077 | Chr8 | - | Coffea canephora | IIe |
| g12066.t1 | *UrWRKY70* | chr21 | transcript:CDP12141 | Chr8 | - | Coffea canephora | III |
| g16165.t1 | *UrWRKY68* | chr21 | transcript:CDP17893 | Chr8 | - | Coffea canephora | IIa |
| g17513.t1 | *UrWRKY72* | chr22 | transcript:CDO98748 | Chr3 | - | Coffea canephora | IIc |
